# Theoretical Investigation of Carbon Dioxide Adsorption on Li^+^-Decorated Nanoflakes

**DOI:** 10.3390/molecules26247688

**Published:** 2021-12-20

**Authors:** Igor K. Petrushenko, Nikolay A. Ivanov, Konstantin B. Petrushenko

**Affiliations:** 1 Irkutsk National Research Technical University, 83 Lermontov St., 664074 Irkutsk, Russia; ivnik@istu.edu; 2 AE Favorsky Irkutsk Institute of Chemistry, Siberian Branch of the Russian Academy of Sciences, 1 Favorsky St., 664033 Irkutsk, Russia; ko_petr@irioch.irk.ru

**Keywords:** carbon dioxide, DFT, coronene, graphene, SAPT0

## Abstract

Recently, the capture of carbon dioxide, the primary greenhouse gas, has attracted particular interest from researchers worldwide. In the present work, several theoretical methods have been used to study adsorption of CO_2_ molecules on Li^+^-decorated coronene (Li^+^@coronene). It has been established that Li^+^ can be strongly anchored on coronene, and then a physical adsorption of CO_2_ will occur in the vicinity of this cation. Moreover, such a decoration has substantially improved interaction energy (E_int_) between CO_2_ molecules and the adsorbent. One to twelve CO_2_ molecules per one Li^+^ have been considered, and their E_int_ values are in the range from −5.55 to −16.87 kcal/mol. Symmetry-adapted perturbation theory (SAPT0) calculations have shown that, depending on the quantity of adsorbed CO_2_ molecules, different energy components act as the main reason for attraction. AIMD simulations allow estimating gravimetric densities (GD, wt.%) at various temperatures, and the maximal GDs have been calculated to be 9.3, 6.0, and 4.9% at T = 77, 300, and 400 K, respectively. Besides this, AIMD calculations validate stability of Li^+^@coronene complexes during simulation time at the maximum CO_2_ loading. Bader’s atoms-in-molecules (QTAIM) and independent gradient model (IGM) techniques have been implemented to unveil the features of interactions between CO_2_ and Li^+^@coronene. These methods have proved that there exists a non-covalent bonding between the cation center and CO_2_. We suppose that findings, derived in this theoretical work, may also benefit the design of novel nanosystems for gas storage and delivery.

## 1. Introduction

Carbon dioxide (CO_2_) is a greenhouse gas that has severe environmental and health effects. An important source of atmospheric CO_2_, which is responsible for climate change, is fossil fuel consumption. The permanent increase of the concentration of carbon dioxide in the atmosphere is a menace to the safety of living species, and it renders a major problem to the environment. Numerous steps have been proposed to overcome this problem, such as the use of renewable energy, a decrease of deforestation, and the decarbonization of buildings and infrastructure. These pathways need sophisticated equipment and large assets; therefore, the idea of direct capture and further conversion of CO_2_ becomes very appealing.

In recent times, research efforts have been focused on the enhancement of current CO_2_ capture methods to alleviate anthropogenic greenhouse gas emissions. There exist a number of carbon dioxide uptake techniques, which depend on such parameters of the gas to be adsorbed including the flow, concentration, temperature, and pressure. In that regard, the well-established technology is the chemical absorption in liquid amines. This methodology, though, holds numerous drawbacks, such as low CO_2_ capacity, equipment corrosion, amine degradation by SO_2_, NO_2_, and O_2_ existed in the flue gases, low energy efficiency owing to the high temperatures utilized during absorbent regeneration, and, finally, large size of the equipment [1].

For the more efficient cyclic usage, the capture techniques based on physical adsorbents are seemingly preferred. A large variety of solid-state media have been systematically investigated for CO_2_ adsorption, e.g., zeolites, aluminophosphates, pristine and doped activated carbons, carbon nanotubes, graphene-based materials, metal organic frameworks (MOFs), and different nanomaterials [2,3,4,5,6,7,8,9,10,11,12,13,14].

Carbon-based adsorbents (e.g., activated carbon, graphene, carbon nanotubes, etc.) have been widely used for CO_2_ uptake owing to their low cost, widespread availability, and high thermal stability. Physical adsorption in this case is driven by London dispersion forces, and, generally speaking, these forces are weak. Consequently, the applications are limited to a high gas pressure or low temperatures. Nowadays, researchers focus on two ways of CO_2_ storage capacity, namely, fabricating a pore structure or modification of surface to increase alkalinity. These methods are principally applicable to graphene. A decade ago, graphene attracted considerable attention owing to its unique structure and impressive mechanical, thermal, and electrical properties [15,16,17]. It was also used as an adsorbent for gas storage [18,19]. However, pristine graphene does not seem to be used as a powerful adsorbent because its electronic structure is responsible for establishing London dispersion forces with molecules accommodated at its surface [19]. More specifically, graphene exhibits a comparatively good adsorption strength for CO_2_, that is stronger than those for the main constituent of natural gas (CH_4_) and the main atmosphere gas (N_2_) [20,21]. At the same time, pristine graphene is a one-atom-thick two-dimensional material, and this structural feature renders it to be a feasible, but low-capacity, gas adsorbent.

The weight percent (wt.%) ratio for the gas uptake can be sufficiently increased by constructing three-dimensional (3D) graphene-based architectures [22,23]. Another technique for the increase in the gas capture is the graphene decoration with atoms or cations of alkali, alkali-earth, and transition metals [24,25,26,27,28]. It is essential to note the successful implementation of the last two methods leads to construction of Li-decorated pillared graphene frameworks with H_2_ uptake of 20.68 wt.% [29]. These techniques are also feasible for the increase in CO_2_ adsorption. Thus, for example, porous graphene frameworks have been shown to provide a substantial enhancement of both H_2_ and CO_2_ adsorption capacity. The recent studies on CO_2_ interactions with Al-decorated, Ca-decorated, Se-decorated, Pt-doped, Ni-, and Co-doped graphene have been reported [25,30,31,32,33,34]. Thus, there are reasons to believe that such a method can enhance CO_2_ adsorption, and decorated graphenes may be considered as candidates for CO_2_ storage. 

In this work, we focus on studying CO_2_ adsorption on the Li^+^-decorated coronene (Li^+^@coronene) model, which can be considered as a small graphene nanocluster. The data obtained from the density functional theory (DFT) method are complemented with energy calculations by using the symmetry-adopted perturbation theory (SAPT0) method [35], which allows obtaining the constituents of interaction energy. The independent gradient model (IGM) method [36,37], Bader’s theory of atoms and molecules (QTAIM) [38] as well as ab initio molecular dynamics (AIMD) computations help us to predict the behavior of CO_2_ molecules in the vicinity of Li^+^@coronene and its adsorption properties at different temperatures. In this regard, this theoretical investigation goes beyond the pristine graphene studies, and broadens our understanding of the mechanisms of a CO_2_ uptake using decorated graphene.

## 2. Results and Discussions

### 2.1. CO_2_ Adsorption on Li^+^@coronene Complexes

**Figure 1:**
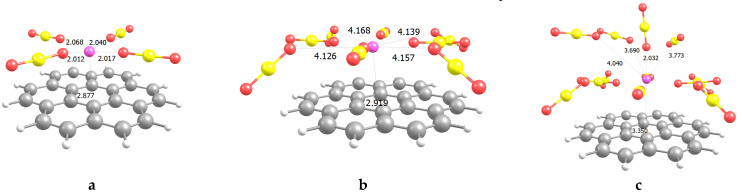
Four (**a**), eight (**b**) and twelve (**c**) CO_2_ molecules adsorbed on Li^+^@coronene. The distances are in Å.

To explicitly study carbon dioxide adsorption on Li^+^@coronene complexes, we add CO_2_ molecules above the surface (in the vicinity of Li^+^) in the step-by-step mode. Optimized geometries (BLYP-D3/def2-SVP) of several complexes are presented in Figure 1. We divide adsorbed CO_2_ into three layers, and each of them contains four CO_2_ molecules (twelve molecules in total). The behavior of the CO_2_ molecules in these layers is different. First, we check the distances between the Li cation and the substrate after optimization when four, eight, and twelve molecules are considered. The distance is constantly increasing, and its values are calculated to be 2.877, 2.919, and 3.350 Å in the case of four, eight, and twelve adsorbed CO_2_ molecules, respectively (Figure 1). The obtained values are significantly larger than that for the single Li^+^ adsorbed at coronene (1.874 Å [39]). Hence, we should further examine the behavior of such complexes in detail. 

For the first layer, we can easily determine that all the CO_2_ molecules lie in one plane, which is parallel to the plane of the adsorbent. The distances between the O atom of the CO_2_ molecule and Li^+^ are in the range of 2.012–2.068 Å (Figure 1). It has been established elsewhere that the optimized distances (*d*) between CO_2_ and a pentagraphene nanosheet are in the range of 3.064–3.212 Å [40]. Very recently, the *d* value between the CO_2_ molecule and the graphene quantum dot (GQD) has been assessed to be 3.615 Å, and E_int_ for CO_2_ adsorption on GQD has been calculated to be −0.39 kcal/mol [24]. This distance is typical for physisorption, but such a low E_int_ value is not generally sufficient for CO_2_ storage. The transition metal (TM) adatoms doping increases the E_int_ values for CO_2_@TM@GQD adsorption [24]. Thus, for example, Co decoration helps one to reach E_int_ of ~−17.30 kcal/mol (*d* = 2.300 Å), Cr decoration increases E_int_ up to ~−27.67 kcal/mol (*d* = 1.562 Å), whereas Ti-decorated GQD demonstrates very high E_int_ values of ~−46 kcal/mol (*d* = 1.633 Å) [24]. However, in the last case, the CO_2_ molecule undergoes dissociation, and it seems that a chemical bond between the adsorbate and the adsorbent occurs. It should be noted that the values discussed above concern only a single CO_2_ molecule adsorbed. Another work, studying CO_2_ adsorption on pristine and defected graphene, yields the following values: E_int_ of −5.37 kcal/mol and *d* of 2.983 Å [41]. The results of that paper also support our outcomes on very marginal, although favorable, interactions between CO_2_ and pristine graphene [41]. Cabrera-Sanfelix [42] and Liu and Wilcox [43] reported the E_int_ magnitudes of ~−3.14 kcal/mol (*d* = 3.47 Å) and −4.84 kcal/mol (*d* = 3.45 Å) for CO_2_ adsorption on defected graphene, respectively. At the same time, experimental studies demonstrated E_int_ of −0.55 (for adsorption on SWCNTs) [44], −4.1 [45], and from −4 to −9 kcal/mol [46], respectively. The results presented above witness that the typical distances between the graphene both pristine and defected are slightly larger than 3 Å, whereas atomic/cation decoration increases the E_int_ magnitudes, but decreases the *d* values. In terms of both distances and energy (Table 1) the values obtained herein are intermediate ones compared with literature data. 

In the present case, the molecules of the second layer acquire the tilted configuration; however, we link this behavior with adsorption at the edge of our model. In general, the *d* values of 4.126–4.168 Å are significantly larger than those for typical physisorption. On the contrary, the CO_2_ molecules representing the third layer show the distances of intermediate magnitudes compared with the previous cases (2.032–4.040 Å) (Figure 1). It witnesses in favor of considering these molecules as adsorbed ones. 

We next turn to comparison of E_int_ and different energy constituents for CO_2_ adsorption (Table 1). The ideal adsorption energy window for CO_2_ storage is from −9.22 to −18.45 kcal/mol (from −0.4 to −0.8 eV) [25].

The first four CO_2_ molecules attached are of especial interest as they show the highest E_int_ values. Their magnitudes are in the range from −16.87 to −11.26 kcal/mol. These values correspond well the optimal energy range for a CO_2_ uptake. They are characterized by high E_el_ and E_ind_ magnitudes, whereas E_disp_ is slightly lower. The non-zero quadrupole moment of the CO_2_ molecule promotes large E_el_ magnitudes, whereas Li^+^ shifts the electronic density of the CO_2_ molecules, and, thereby, enhances the E_ind_ term. At the same time, the small quantity of the neighbor molecules accounts for the small dispersion interactions; the similar situation was observed earlier for hydrogen adsorption in hollow pores [47] or Li^+^-decorated nanostructures [48,49,50]. A close analysis was carried out for the next four molecules adsorbed. One may easily note the significant decrease in E_int_ values compared with the previous case. Besides this, the contribution of E_ind_ to the total attractive interactions is rather small here, and it gives only one tenth of all attractive interactions. The significance of London dispersion is much higher, and the contributions of E_disp_ and E_el_ terms are nearly equal (Table 1). 

Finally, for the last four CO_2_ molecules, the trend slightly varies, and one can observe the intermediate E_int_ values, which are significantly lower compared with the first four molecules. However, they are high enough to be superior compared with the second four molecules. Indeed, the E_disp_ term diminishes as the small quantity of CO_2_ is located above the Li cation. At the same time, E_ind_ is much higher as the positions of the adsorbed molecules are closer to Li^+^ (Figure 1, Table 1). To sum up, the first four molecules are described by the highest E_int_, the second four molecules exhibit the smallest E_int_, and the E_int_ values for the last four CO_2_ molecules fall between them. We suppose that 1–4 CO_2_ molecules should play a major role in determining the CO_2_ uptake value for Li^+^@coronene, but we should keep in mind 9–12 CO_2_ molecules and thoroughly study the behavior of the systems in detail further. 

Now we compare the obtained E_int_ energies with those obtained elsewhere. Lu et al. studied CO_2_ adsorption on pentagraphene, and the E_int_ energies were in the range from −3.44 to −4.82 kcal/mol. Darvishnejad and Reisi-Vanani investigated CO_2_ adsorption on pristine and Sr-decorated graphynes (Gy). It was established that E_int_ values for the first adsorbed CO_2_ molecule are −5.81 and −9.80 kcal/mol for Gy and Sr-decorated Gy, respectively [51]. According to Ref. [25], the obtained E_int_ values for 1–4 CO_2_ molecules fall inside the optimal range for CO_2_ capture. Besides, E_int_ of some of the 9–12 CO_2_ molecules also fall into this energy window. Moreover, E_int_ values obtained herein are generally higher than those obtained elsewhere.

To visualize the interactions between CO_2_ molecules and Li^+^@coronene, we implement here the independent gradient model (IGM) method [36,37]. Lefevbre et al. implemented this method as a straightforward tool to study inter- and intramolecular interactions. The new descriptors which are defined in the framework of this method allow obtaining a measure of electron sharing (δg), and separately describe interactions inside each molecular fragment (δg^intra^) or between fragments (δg^inter^). According to the definition, the blue color regions indicate strong binding (e.g., hydrogen bonds), and the green color ones represent weak interactions (e.g., van der Waals interactions).

**Figure 2:**
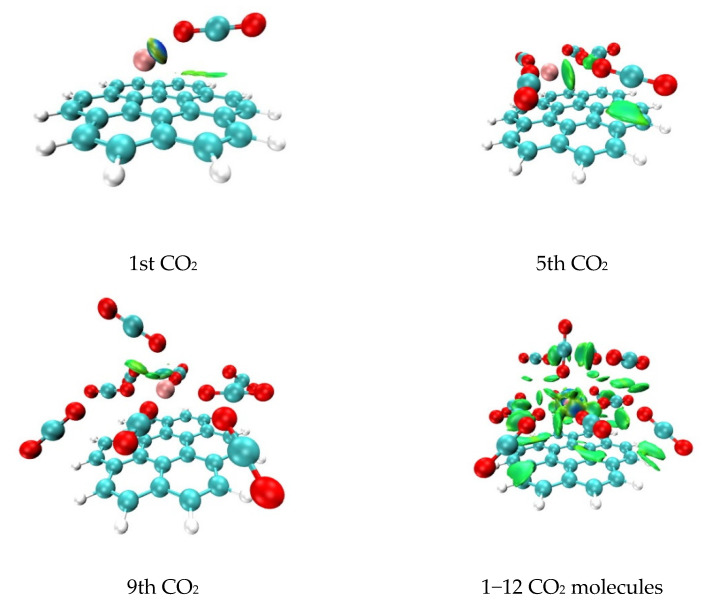
Independent gradient model (IGM) analysis of CO_2_ adsorption (isovalue = 0.01). Atomic color code: carbon—blue-green, hydrogen—white, oxygen—red, lithium cation—pale red. Color code: green-colored lobes denote weak non-covalent interactions, blue lobes denote strong attractive interactions.

As shown in Figure 2, for the first CO_2_ molecule adsorbed on Li^+^@coronene, there exist a green-blue lobe in the vicinity of Li^+^, which denotes a strong attraction and a purely green lobe (between the CO_2_ molecule and the coronene surface), which denotes weak van der Waals interactions. We then inspect the case of the fifth adsorbed CO_2_ molecule. The scene changes significantly, and we can observe only three green lobes and no blue-green ones. It accounts for the increased significance of London dispersion and the decrease contribution of induction interactions into attraction. The interaction of the 9th molecule with the system is characterized by the small quantity of green lobes, which denotes generally lower dispersion contribution, and the very small blue-green isosurface. These observations are in line with the results of energy decomposition analysis by the SAPT0 method (Table 1). These cases treat the adsorbed system as two fragments, one of which is the CO_2_ molecule (first, fifth, or ninth) and another one is the rest molecules and the Li^+^@coronene substrate. The fourth studied system is the one including twelve adsorbed CO_2_ molecules. Here, we treat the system as thirteen isolated parts (one of them is Li^+^@coronene and another twelve are studied CO_2_ molecules). The results are presented in Figure 2 (bottom, right). It approves our previous outcomes that for one to four adsorbed CO_2_ molecules, E_ind_ plays an important role (a blue-green isosurface in the vicinity of Li^+^). On the contrary, a large number of green lobes validate our conclusions on the important role of E_disp_ in the case of five to eight adsorbed CO_2_ molecules. Finally, the smaller density of green lobes for nine to twelve CO_2_ molecules indicates the decrease of the E_disp_ contribution into total attraction interactions and the simultaneous increase of E_el_ and E_ind_ ones (cf. Table 1). 

### 2.2. Analysis of Non-Covalent Interactions between CO_2_ Molecules and the Li^+^@coronene Complex by Quantum Theory of Atoms in Molecules (QTAIM)

To obtain profound information on the nature of interactions between CO_2_ molecules and the studied adsorbent, we involve here the Bader’s quantum theory of atoms in molecules (QTAIM). We obtain such quantities as: electron density (ρ(r)) at the (3, −1) bond critical point (BCP), its Laplacian (∇^2^ρ(r)), the electronic energy density (H(r)), the kinetic energy density, G(r), and the potential energy density, V(r). These values are summarized in Table 2.

First, it is worth noticing that no BCPs between five to eight CO_2_ molecules and the Li cation are observed. It witnesses that smaller E_int_ values (Table 1) for these molecules are mainly due to E_disp_ interaction between CO_2_ molecules, and the interactions between the cation center and these molecules are feeble ones. The values of electron density (ρ(r)) at the BCPs provide valuable information on the strength of bonding. The magnitudes of ρ(r) are in line with E_int_ values obtained at the SAPT0 level of theory. As is expected, the first adsorbed molecules are described by the significant magnitudes of E_int_. The positive (negative) values of ∇^2^ρ(r) at the BCPs indicate a non-covalent (covalent) bond existence [52]. Table 2 shows that physisorption is the sole type of adsorption here. Besides this, we inspect the H(r) values, and the positive sign of all the values exhibits the non-covalent interactions [53]. The last criterion we inspect is the magnitude of the following ratio: −G(r)/V(r). As it is larger than unity, the interactions can be classified as the non-covalent ones.

### 2.3. AIMD Simulations of CO_2_ Adsorption on Li^+^@coronene

For the sake of completeness, we carry out the ab initio molecular dynamics (AIMD) simulations to inspect the thermal stability of adsorbed Li cations as well as CO_2_ molecules. Additionally, the data obtained is utilized for CO_2_ gravimetric density calculations (GD, wt.%) at different temperatures. In this work, we use three temperatures (*T*): 77 K (liquid nitrogen *T*), 300 K (room *T*), and 400 K (elevated *T* for a flue gas). 

First, we study the system consists of four CO_2_ molecules adsorbed on Li^+^@coronene (Figure S1, Supplementary Materials). For the lowest studied *T* = 77 K, and at this moment of time (*t*) = 500 fs, we can observe only small reorientations of carbon dioxide molecules adsorbed at Li^+^. All the CO_2_ molecules can be considered as adsorbed at *t* = 500 and 1000 fs. Only minute structural changes of Li^+^@coronene can be observed in this case. However, the significant displacement of Li^+^ from the origin is worth mentioning. Earlier it was established that the edge six-membered cycles of PAHs can accommodate Li cations more favorably than the central ones [54]. Besides, it appears that potential barriers for Li^+^ migration on the surface of PAHs are rather small. At room *T* = 300 K, no significant changes can be noted. Even at the elevated *T* = 400 K and *t* = 1000 fs, the CO_2_ molecules all acquire tilted configuration, but the distancing from Li^+^ is feeble. 

Second, we take into consideration eight CO_2_ molecules adsorbed (Figure S2, Supplementary Materials). For *T* = 77 K, the small shift of Li^+^ and adsorbed CO_2_ molecules from the initial position (at *t* = 500 and 1000 fs) can be noted. However, even at *t* = 1000 fs, all the molecules are at nearly the same distances from Li^+^ compared with the initial moment of time. For *T* = 300 K, it can be easily observed that the four molecules, which comprise the second layer, are well spaced (at *t* = 500 and 1000 fs). For *T* = 400 K, a sufficient displacement of Li^+^ occurs, and only first four CO_2_ molecules can be considered as adsorbed; the rest ones are at very large distances from Li^+^.

Finally, we study adsorption behavior of twelve CO_2_ molecules (Figure S3, Supplementary Materials). For *T* = 77 K, we also note only small deviations of both Li^+^ and CO_2_ molecules from the initial positions. For *T* = 300 K and *t* = 1000 fs, the adsorbed system can be characterized by the moderate distancing of some CO_2_ molecules, which constitute the second layer, from Li^+^. Moreover, for *T* = 400 K and *t* = 1000 fs, not only the above-mentioned molecules, but also molecules from the third layer become well spaced from the Li cation. In the last case, only four CO_2_ molecules can be considered as adsorbed. 

**Figure 3:**
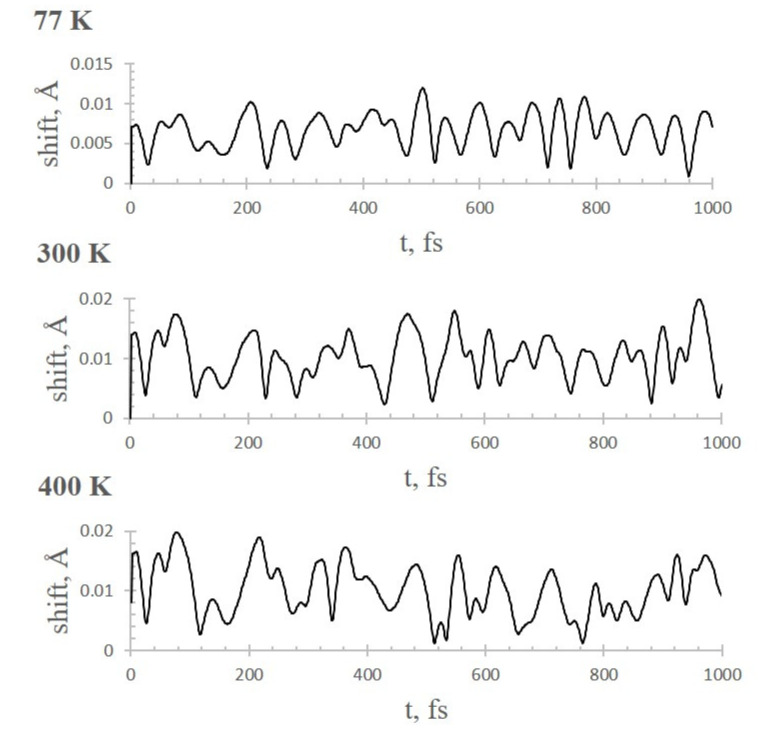
Time evolution of Li^+^ shifts between each pair of consecutive steps at three studied temperatures.

We also identify the thermostability of the Li^+^@coronene complex itself with 12 adsorbed CO_2_ molecules for the liquid nitrogen temperature (77 K) and for the general operating mode (300 and 400 K). We inspect the distances between all pairs of the consecutive steps (e.g., the initial position of the Li cation is fixed as the origin and its position after 1.0 fs of equilibration is the position at the second step) during the simulation time, and then calculate the shifts between each pair of steps. As it can be easily seen in Figure 3, the Li^+^-coronene distance is still stable during the whole simulation time. The Li^+^ shifts fluctuate around the mean values of ~0.005, 0.010, and 0.010 Å for 77, 300 and 400 K, respectively. Additionally, the results obtained show the very similar patterns for *T* = 300 and 400 K. The displacements of Li^+^ (at the end of simulation (i.e., *t* = 1.0 ps)) are measured to be ~1.45, 3.25, and 4.00 Å for 77, 300, and 400 K, respectively. The large traveling distance of Li^+^ for the elevated temperatures require careful monitoring of the complexes (Figure S2 and S3, Supplementary Materials). As it was established above, we observe the large distancing of several CO_2_ molecules, but the Li^+^@coronene complex as a whole can be considered stable for CO_2_ adsorption.

Using the present AIMD data and the results of DFT calculations derived above, we now assess the CO_2_ uptake by the Li^+^@coronene system. The CO_2_ gravimetric density (*GD*, wt.%) may be estimated as:

(1) $$GD=\frac{M_{nCO_{2}}}{M_{nCO_{2}}+M_{complex}}\times 100\%$$

where, $M_{nCO_{2}}$ is the mass of *n* adsorbed CO_2_ molecules, and $M_{complex}$ is the mass of the complex.

We calculate several GDs for the Li^+^@coronene complex. The first GD value is calculated using our DFT and SAPT0 results, and it includes all twelve molecules adsorbed. As some of the molecules are weakly linked with the cation center, we adopt this value as the highest limit of CO_2_ uptake, and it is calculated to be 7.3 (13.35) wt.% for one-side (double-side) adsorption. Furthermore, the QTAIM computations derived above approve our outcomes on the weakly bound five to eight CO_2_ molecules. We, hence, exclude them from the further considerations. AIMD simulations can help one to reveal the molecules both adsorbed and released as well as quantify their number. Table 3 shows that the increase in *T* leads to the substantial decrease in GDs.

Now, it is worth comparing the GD values tabulated above with those on carbonaceous adsorbents obtained elsewhere. It was established that the typical CO_2_ adsorption capacity of activated carbons is 5 wt.% [55,56]. Zhao et al. demonstrated adsorption capacity of 4.65 wt.% at 303 K and 1 bar for CO_2_/N_2_ mixture for ethylenediamine (EDA)-intercalated graphene oxide [57]. Surface-microporous 3D graphene has been shown to provide high CO_2_ capacity of ~10%. The KOH activation further increases its capacity up to ~13.8% [22]. Different mesoporous molecular sieves exhibited 3.3–13.3% carbon dioxide uptake [1]. At the same time, a theoretical assessment of CO_2_ capacity of pristine and Sr-decorated graphyne shows very high values, namely, 18.63 and 31.68 wt.%, respectively [51]. Thiruvenkatachari et al. synthesized honeycomb-shaped carbon fiber adsorbents for CO_2_ capture. The average CO_2_ adsorption uptake was shown to be 11.9 wt.% for a gas mixture [58]. The comparison clearly shows that the proposed complex exhibits considerable CO_2_ physisorption, and, hence, we believe that the studied system owing to its simplicity can help the researchers to design efficient new systems for CO_2_ capture. This work, presumably, will be useful for waste gases treatment to decrease the negative impact from industrial plants and vehicles.

## 3. Computational Methods

### 3.1. Computational Details

Geometries of all structures studied herein have been optimized using the BLYP [59,60] density functional in conjunction with the def2-SVP [61] basis set. Grimme’s dispersion correction (D3) [62] has been employed for the accurate treatment of van der Waals interactions. The BLYP-D3 method has been shown to provide reliable results on different graphene nanoflake complexes [63,64,65,66,67]. Frequency calculations have been performed to validate equilibrium geometries.

The interaction energy (E_int_) between the adsorbent and the adsorbate molecules has been calculated for all the studied complexes using zero*th*-order symmetry-adapted perturbation theory (SAPT0) [35] with the jun-cc-pVDZ basis set [68,69]. This method helps one to decompose E_int_ into the meaningful components: exchange (E_ex_), electrostatic (E_el_), dispersion (E_disp_), and induction (E_ind_) ones. We involve the PSI4 code (v.1.2) [70] to perform SAPT0 calculations, whereas the Orca 4.2.1 [71] program has been used for geometry optimizations. It is worth noticing that a more negative E_int_ value denotes a stronger interaction. Additionally, the SAPT0 interaction energies are free from the so-called basis set superposition errors (BSSE). The SAPT0 description is given in Supplementary Materials in more detail. The procedure of obtaining energy constituents was the following: first, the geometries of Li^+^@coronene along with the adsorbed CO_2_ molecules were optimized at the BLYP/def2-SVP level of theory. Then, the SAPT0 method was used to obtain the magnitudes of aforementioned energy terms. 

The initial structures for ab initio molecular dynamics (AIMD) simulations have been optimized using the BLYP/def2-SVP method. AIMD computations have been also done by the Orca 4.2.1 program suite. All AIMD simulations used a time step of one femtosecond, a canonical constant volume and constant temperature NVT ensemble using a Berendsen thermostat [72] and a velocity Verlet algorithm for integrating the equation of motion. The time period of 1000 femtoseconds (fs) has been used for the full AIMD computation cycle. The time step has been chosen to be 1 fs. 

### 3.2. Validation of Methodology 

First, it should be noted here that the combination of methods (BLYP/def2-SVP) suits well for the study of adsorption phenomena. Besides, the SAPT0/jun-cc-pVDZ method is shown to provide very reliable results for non-covalent interactions [73]. 

Second, to further validate the methodology used, we compare the obtained E_int_ value for Li^+^ adsorption on coronene (−40.52 kcal/mol) with those for the relevant systems. The result of Gal et al. (at the B3LYP/6-311G(3df,2p) level of theory) is of ~−44.46 kcal/mol for Li^+^@coronene interactions. E_int_ calculated for Li^+^ adsorption on triphenylene central ring is of −44.4 kcal/mol [74]. As studies on Li^+^ interactions with PAHs are rather scarce, we compare the obtained values with those on Li^+^@benzene interactions (a benzene molecule can be adopted as a central ring of coronene). The experimentally observed E_int_ for Li^+^@benzene adsorption are as follows: −38.3 [75] and −38.5 ± 3.2 kcal/mol [76].

Our previous E_int_ result on Li^+^ adsorption on benzene (MP2/SAPT2) has shown to be −36.92 kcal/mol [77]. The E_int_ values for Li^+^ adsorbed on curved PAHs were also given as an instance. Thus, Li^+^ adsorption on concave and convex sites of corannulene (C_20_H_10_) and sumanene (C_21_H_12_) yields −44.57, −44.87 kcal/mol [78] and −40.48, −37.97 kcal/mol [79], respectively (these values are for adsorption on the central rings of the sorbents). At the same time, adsorption on the central hexagonal ring of circumtrindene (C_36_H_12_) exhibits slightly higher values of −46.83 and −46.18 kcal/mol for the concave and convex site, respectively [80]. Hence, it seems evident that the obtained result is in a reasonably good accordance with the available literature data. The difference can be explained by the various computational methods involved.

## 4. Conclusions

To summarize, the Li^+^@coronene complex was theoretically studied as a new platform for a carbon dioxide capture. DFT and SAPT0 calculations of the 1–12 CO_2_ molecules adsorbed at Li^+^@coronene exhibit larger E_int_ values compared with those for pristine graphene, however, some of them do not satisfy the optimal energy range for CO_2_ storage. At the same time, the AIMD results show that the cation center of the Li^+^@coronene complex can favorably accommodate numerous CO_2_ molecules that leads to the maximal gravimetric density (GD) of 5.0 (9.3) wt.% for one-side (double-side) adsorption. 

The SAPT0 analysis also reveals that the different ratio of the interaction energy terms affects CO_2_ molecules attached. The first four CO_2_ molecules are mainly attracted by electrostatic forces, whereas dispersion and induction energy terms are of less importance. For the next four CO_2_ molecules adsorbed, electrostatic and dispersion terms are equally important, and only one tenth of the whole attraction is due to dispersion. These molecules are characterized by the lowest E_int_ values. For nine to twelve CO_2_ molecules, large influence of electrostatic interactions can be observed, they are followed by the dispersion and induction ones. The IGM analysis visually complements the outcomes of SAPT0 calculations, whereas QTAIM calculations approve the existence of weak non-covalent interactions between the CO_2_ molecules and Li^+^@coronene. AIMD simulations reveal stability of the Li^+^@coronene complexes. At *T* of 77 K, we note that all the molecules are at the anchoring cation during the whole process of simulation. However, for CO_2_ adsorption at *T* = 300 K and especially at *T* = 400 K, the considerable distancing of the molecules is observed; it leads to the decrease of GDs. Thus, involving a set of theoretical methods that reveal the nature of CO_2_/adsorbent interactions, our paper establishes that the Li^+^@coronene cluster may be useful as a novel medium for the carbon dioxide storage.

## Figures and Tables

**Table 1:** SAPT0/jun-cc-pVDZ energies (electrostatic (E_el_), exchange (E_ex_), induction (E_ind_), dispersion (E_disp_), and interaction (E_int_)) for CO_2_ adsorption. The percentage contributions into attractive interactions are given in parentheses. All energies are in kcal/mol.

No. CO_2_	E_el_	E_ex_	E_ind_	E_disp_	E_int_
1	−14.34 (50)	11.98	−8.99 (31)	−5.52 (19)	−16.87
2	−13.83 (47)	13.06	−8.48 (29)	−7.32 (25)	−16.57
3	−13.4 (49)	12.79	−7.11 (26)	−6.57 (24)	−14.29
4	−11.08 (45)	13.15	−5.44 (23)	−7.89 (32)	−11.26
5	−6.44 (44)	9.03	−1.58 (10)	−6.75 (46)	−5.73
6	−7.52 (46)	9.36	−1.74 (11)	−6.95 (43)	−6.85
7	−7.02 (46)	9.04	−1.62 (11)	−6.65 (43)	−6.24
8	−5.96 (44)	7.9	−1.37 (10)	−6.13 (46)	−5.55
9	−6.88 (48)	7.29	−3.29 (22)	−4.28 (30)	−7.16
10	−8.65 (47)	9.92	−3.71 (20)	−5.96 (33)	−8.94
11	−10.11 (48)	11.47	−4.24 (20)	−6.85 (32)	−9.73
12	−5.94 (48)	6.31	−1.48 (12)	−5.03 (40)	−6.14

**Table 2:** Selected QTAIM topological parameters (a.u.) for the systems studied.

Complex	ρ(r)	∇^2^ρ(r)	H(r)	V(r)	G(r)
1st CO_2_	0.0279	0.2117	0.0121	−0.0287	0.0408
2nd CO_2_	0.0271	0.2084	0.0121	−0.0279	0.0400
3rd CO_2_	0.0246	0.1913	0.0113	−0.0252	0.0365
4th CO_2_	0.0160	0.1145	0.0065	−0.0156	0.0221
9th CO_2_	0.0144	0.0969	0.0050	−0.0143	0.0193
10th CO_2_	0.0068	0.0238	0.0005	−0.0049	0.0054
11th CO_2_	0.0094	0.0415	0.0019	−0.0067	0.0085
12th CO_2_	0.0077	0.0332	0.0016	−0.0052	0.0067

**Table 3:** Carbon dioxide GDs (wt.%) obtained from DFT and AIMD calculations.

Li^+^@coronene			
GD, DFT	GD, AIMD		
-	77 K	300 K	400 K
5.0 (9.3) ^1^	5.0 (9.3)	3.2 (6.0)	2.6 (4.9)

^1^ Values in parentheses denote GDs upon double-side adsorption.

## Supplementary Materials

Figure S1: AIMD simulations snap shots of four adsorbed CO_2_ molecules on Li^+^@coronene complexes at T = 77 K (A), 300 K (B), 400 K (C). Atomic color code: carbon—yellow, oxygen—red, hydrogen—light-blue, lithium cation—magenta. Figure S2. AIMD simulations snap shots of eight adsorbed CO_2_ molecules on Li+@coronene complexes at T = 77 K (A), 300 K (B), 400 K (C). Atomic color code: carbon—yellow, oxygen—red, hydrogen—light-blue, lithium cation—magenta. Figure S3. AIMD simulations snap shots of twelve adsorbed CO_2_ molecules on Li+@coronene complexes at T = 77 K (A), 300 K (B), 400 K (C). Atomic color code: carbon—yellow, oxygen—red, hydrogen—light-blue, lithium cation—magenta.

## Sample Availability

Samples of the compounds are not available from the authors.
