# Supplementary material for: Theoretical Investigation of Carbon Dioxide Adsorption on Li+-Decorated Nanoflakes
Source: Molecules. 2021 Dec 20;26(24):7688. doi: 10.3390/molecules26247688 (PMC8706083; doi:10.3390/molecules26247688)
Supplement: Supplementary file 1 [file molecules-26-07688-s001.zip › molecules-1497306-supplementary.pdf]

molecules

MDPI

Supplementary Materials

Theoretical Investigation of Carbon Dioxide Adsorption on Li⁺-Decorated Nanoflakes

Igor K. Petrushenko [1,*], Nikolay A. Ivanov [1] and Konstantin B. Petrushenko [2]

[1] Irkutsk National Research Technical University, 83 Lermontov st., 664074 Irkutsk, Russia; ivnik@istu.edu
[2] AE Favorsky Irkutsk Institute of Chemistry, Siberian Branch of the Russian Academy of Sciences, 1 Favorsky st., 664033 Irkutsk, Russia; ko_petr@irioch.irk.ru
* Correspondence: igor.petrushenko@istu.edu

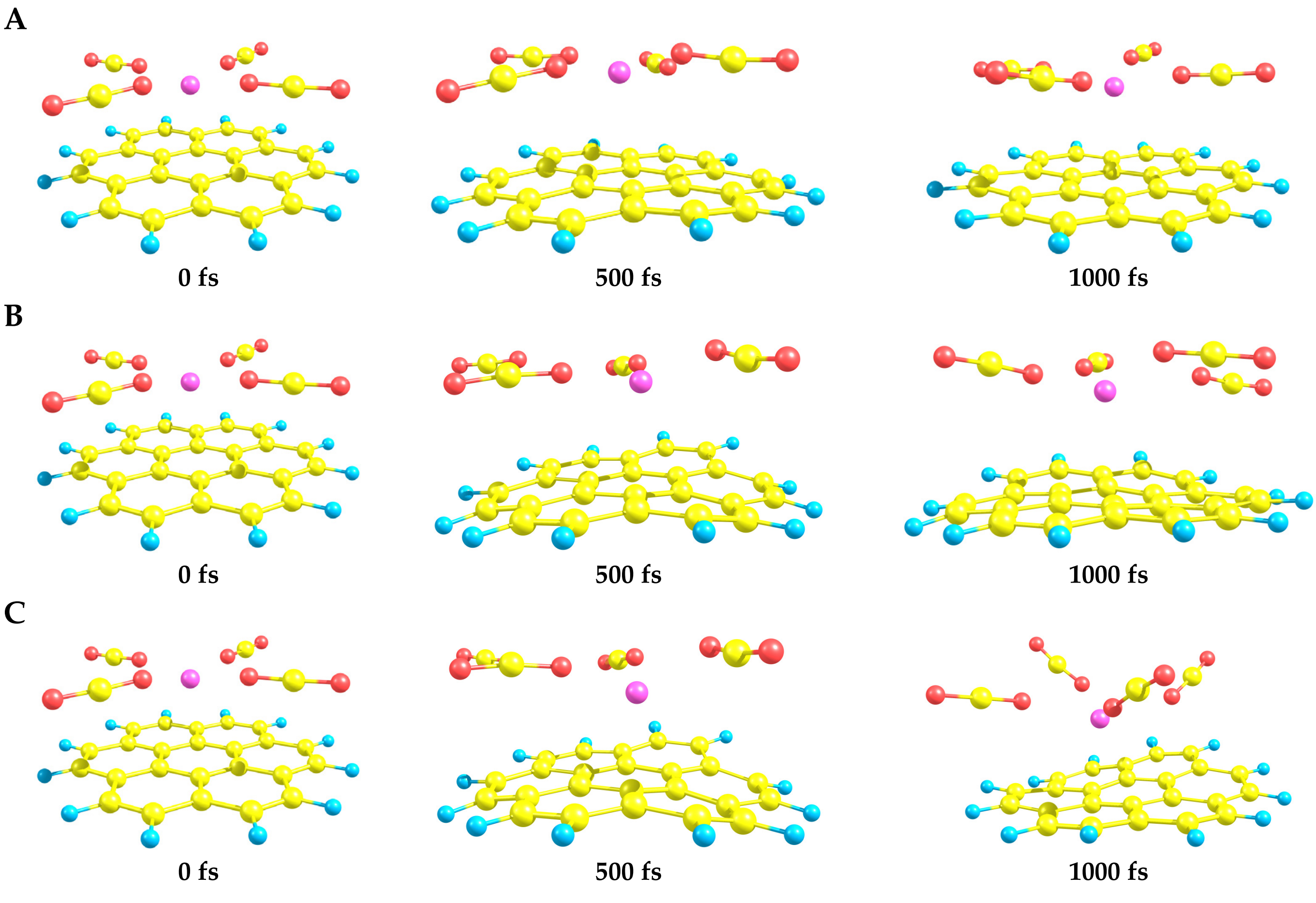

Figure S1. AIMD simulations snap shots of four adsorbed CO_2 molecules on Li^+@coronene complexes at T = 77 K (**A**), 300 K (**B**), 400 K (**C**). Atomic color code: carbon—yellow, oxygen—red, hydrogen—light-blue, lithium cation—magenta.

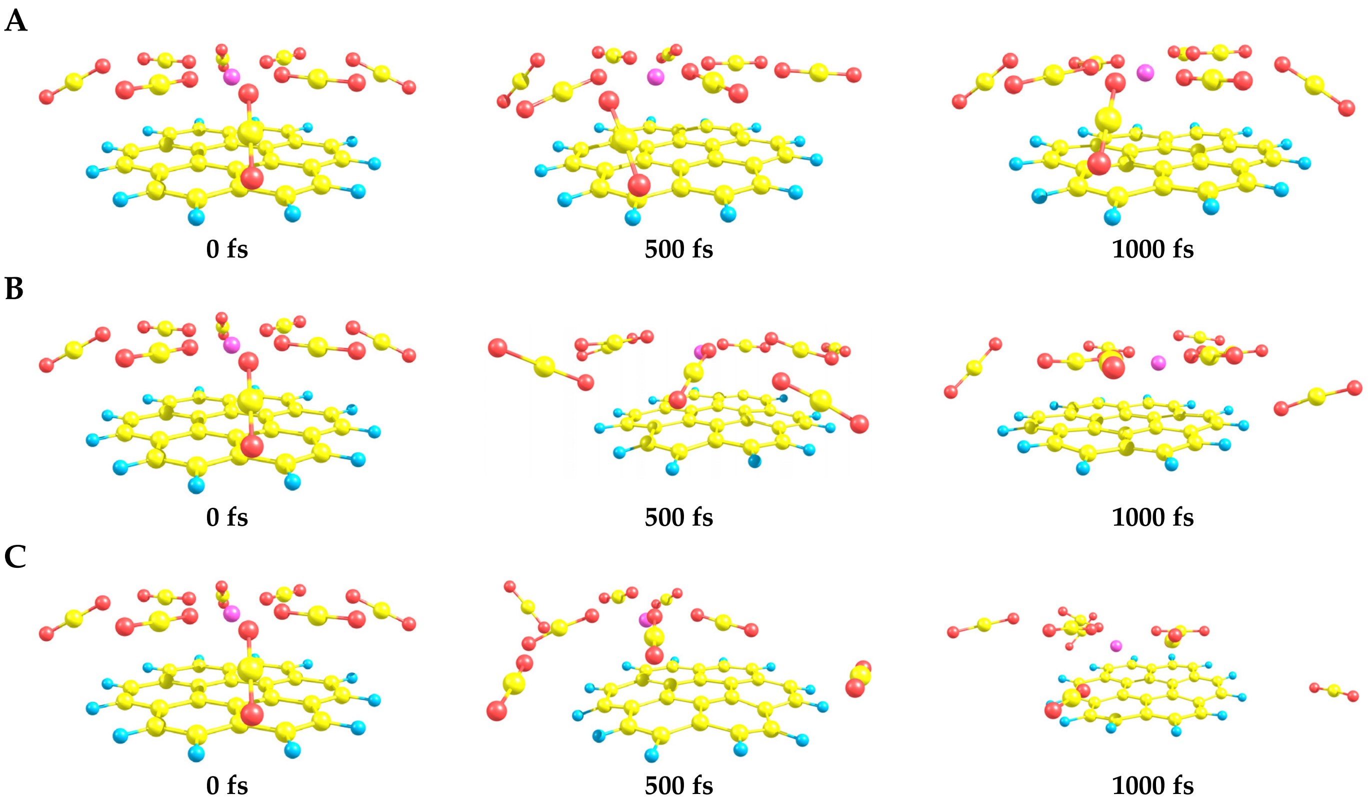

Figure S2. AIMD simulations snap shots of eight adsorbed CO_2 molecules on Li^+@coronene complexes at T = 77 K (**A**), 300 K (**B**), 400 K (**C**). Atomic color code: carbon—yellow, oxygen—red, hydrogen—light-blue, lithium cation—magenta.

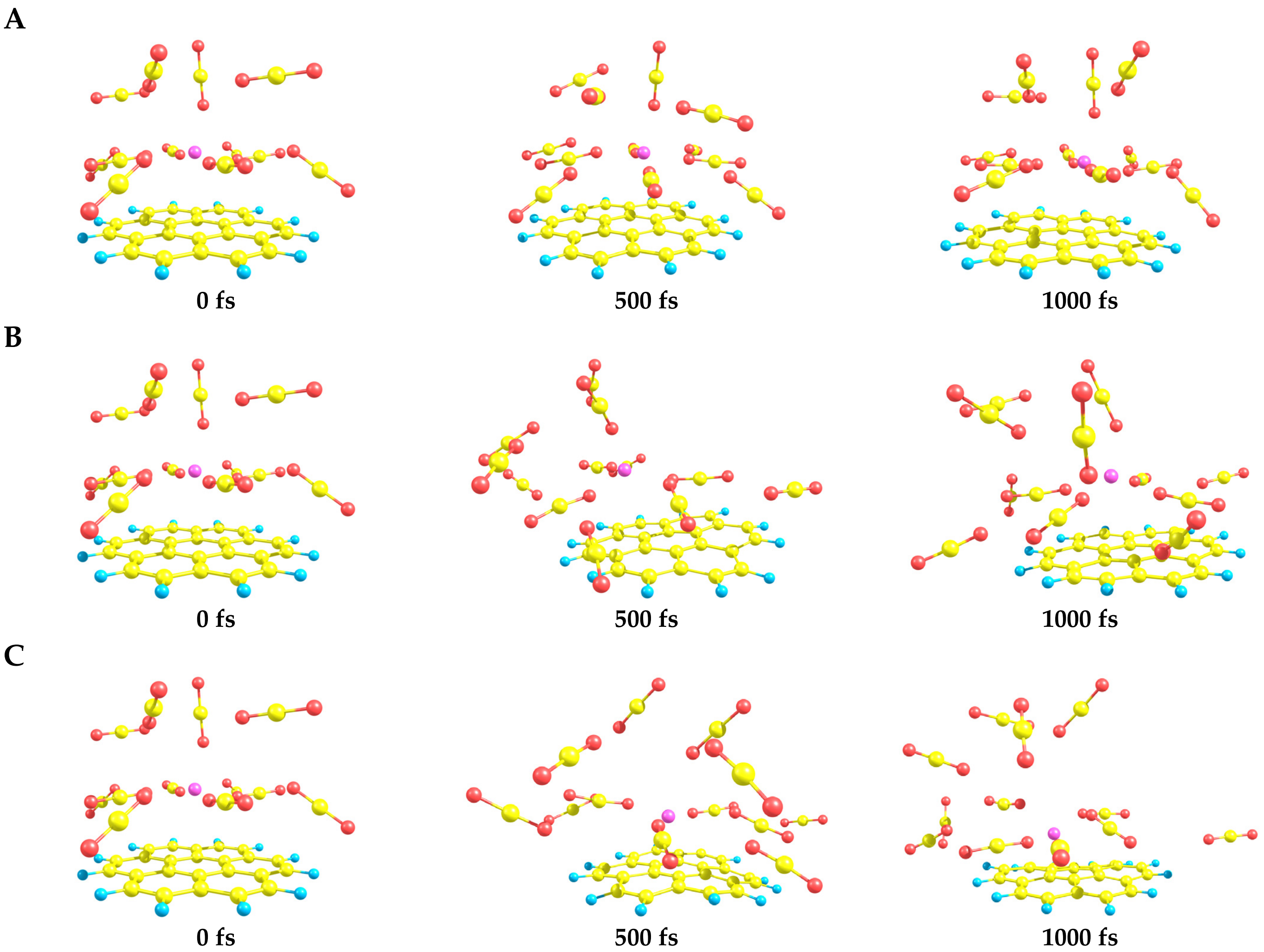

Figure S3. AIMD simulations snap shots of twelve adsorbed CO_2 molecules on Li^+@coronene complexes at T = 77 K (**A**), 300 K (**B**), 400 K (**C**). Atomic color code: carbon—yellow, oxygen—red, hydrogen—light-blue, lithium cation—magenta.

Independent Gradient Model (IGM)

This straightforward approach "provides chemists with a visual understanding of interactions present in chemical systems". The new descriptors, which are defined in the framework of this method, allow obtaining a measure of electron sharing (δg), and separately describe interactions inside each fragment (δg^{intra}) or between fragments (δg^{inter}).

$$\delta g = |\nabla\rho^{\mathrm{IGM}}| - |\nabla\rho|. \tag{S1}$$

$$\delta g^{inter} = |\nabla\rho^{\mathrm{IGM,\ inter}}| - |\nabla\rho|. \tag{S2}$$

$$\delta g^{intra} = |\nabla\rho^{\mathrm{IGM}}| - |\nabla\rho^{\mathrm{IGM,\ inter}}|. \tag{S3}$$

$$\delta g = \delta g^{inter} + \delta g^{intra}. \tag{S4}$$

where δg^{intra} and δg^{inter} are the intramolecular and intermolecular contributions; $\nabla\rho$ is the electron density gradient, $\nabla\varrho^{IGM}$ is the gradient vector of density in independent gradient model; $\nabla\varrho^{IGM,\ inter}$ is the gradient vector, where the interaction between molecules canceled while intramolecular part has been divided into two additions, which corresponds to intramolecular interaction in separate molecules.

Symmetry-Adapted Perturbation Theory (SAPT)

Symmetry-adapted perturbation theory (SAPT) is a method for direct calculations of the non-covalent interaction between two molecules. In other words, the interaction energy is determined without computing the total energy of the monomers or dimer. Thus, the result obtained is free from the basis set superposition error (BSSE). In addition, SAPT provides a decomposition of the interaction energy into meaningful components: *i.e.*, electrostatic, exchange, induction (polarization) and dispersion terms. In SAPT, the Hamiltonian of the dimer is partitioned into contributions from each monomer and the interaction.

$$\boldsymbol{H = F_A + W_A + F_B + W_B + V} \tag{S5}$$

the Hamiltonian (H) is written as a sum of the usual monomer Fock operators (F), the fluctuation potential of each monomer (W), and the interaction potential (V). Fock operators are treated as the zeroth-order Hamiltonian and the interaction energy is evaluated through a perturbative expansion of V, W_A and W_B. Through first-order in V, electrostatic and exchange interactions are included; induction and dispersion first appear at second-order in V.

Several truncations of the closed−shell SAPT expansion are available in PSI4. The simplest truncation of SAPT is denoted SAPT0 and defined in the following equation:

$$\boldsymbol{E_{SAPT0} = E^{(10)}_{elst} + E^{(10)}_{exch} + E^{(20)}_{ind,resp} + E^{(20)}_{exch-ind,resp} + E^{(20)}_{disp} + E^{(20)}_{exch-disp} + \delta^{(2)}_{HF}} \tag{S6}$$

In this notation, $\boldsymbol{E^{(vw)}}$ defines the order in V and in WA + WB; the subscript (resp) indicates that orbital relaxation effects are included.

The $\boldsymbol{\delta^{(2)}_{HF}}$ term takes into account the higher-order induction effects, and it is included in the definition of SAPT terms.

Normally, the adsorption energy terms are calculated as following:

$$E_{ad} = E_{el} + E_{ex} + E_{ind} + E_{disp}, \tag{S7}$$

Where $E_{el} = E^{(10)}_{elst}$, $E_{ex} = E^{(10)}_{exch}$,

$$E_{ind} = E^{(20)}_{ind,resp} + E^{(20)}_{exch-ind,resp} + \delta^{(2)}_{HF}, \tag{S8}$$

$$E_{disp} = E^{(20)}_{disp} + E^{(20)}_{exch-disp} \tag{S9}$$

For the SAPT2 method, we can write down the following equation:

$$\boldsymbol{E_{SAPT2} = E_{SAPT0} + E^{(12)}_{elst,resp} + E^{(11)}_{exch} + E^{(12)}_{exch} + E^{t(22)}_{ind} + E^{t(22)}_{exch-ind}}, \tag{S10}$$

where some second order corrections in energy have been added with amendments to the energy terms mentioned above.

Cartesian Coordinates of Studied Structures

**Li⁺@coronene**			
C	−2.615410255	0.146156238	−2.429314242
C	−2.618361312	1.104650754	−1.365180291
C	−2.633859033	0.625402817	−0.010247911
C	−2.633703683	−0.794269759	0.254447452
C	−2.618074658	−1.728389816	−0.837819959
C	−2.614852532	−1.216827611	−2.175682569
C	−2.630743237	1.564271615	1.087676548
C	−2.634758769	−1.274578413	1.615887922
C	−2.635961013	−0.335719513	2.713827354
C	−2.631770840	1.083970868	2.449126993
C	−2.622504104	−0.814998151	4.068796939
C	−2.621539691	−2.229279794	4.294875563
C	−2.620529598	−3.130488784	3.241753991
C	−2.620766312	−2.687964470	1.879556298
C	−2.616302559	−3.591055129	0.767696782
C	−2.614764030	−3.129492832	−0.539665109
H	−2.612032261	−3.844453829	−1.374619734
H	−2.614871502	−4.672010810	0.968686158
H	−2.613503863	0.512804387	−3.465514989
H	−2.612535434	−1.930738196	−3.011469453
H	−2.621672053	−2.595363043	5.331214956
H	−2.620020753	−4.211220329	3.443333475
C	−2.613255344	2.518923255	−1.591254250
C	−2.610233513	3.420143301	−0.538145505
C	−2.612534937	2.977616217	0.824046306
H	−2.611743284	2.885005581	−2.627593704
H	−2.606403248	4.500865570	−0.739737478
C	−2.606038346	3.880677695	1.935923252
C	−2.606534211	3.419125100	3.243290049
C	−2.614011824	2.018037155	3.541436390
H	−2.601296920	4.961625986	1.734939788
H	−2.602155441	4.134077231	4.078244302
C	−2.617421656	0.143485616	5.132931926
H	−2.617099659	−0.223162591	6.169134000
C	−2.612890249	1.506462770	4.879299613
H	−2.608948997	2.220361692	5.715090420
Li	−0.847040741	0.142593835	1.354549615
**1CO₂@Li⁺@coronene**			
C	−2.193817120	−1.201492351	−2.807036778
C	−2.481997591	−0.326600788	−1.708474189
C	−2.484721537	−0.868120650	−0.376318039
C	−2.177558543	−2.264660720	−0.169190944
C	−1.877296506	−3.113558570	−1.293811329
C	−1.905518786	−2.542929120	−2.608899524
C	−2.762037212	−0.014896508	0.752832159
C	−2.122567069	−2.795412410	1.174428022

C	−2.402307073	−1.938705542	2.300360994
C	−2.722626904	−0.550999635	2.087870714
C	−2.348408628	−2.469801798	3.633162145
C	−2.018673974	−3.853687600	3.808836925
C	−1.745294422	−4.676300831	2.727227253
C	−1.785617125	−4.178031676	1.384650064
C	−1.509037532	−4.999031085	0.242019052
C	−1.552427794	−4.488216056	−1.046207449
H	−1.333490738	−5.139365651	−1.904268965
H	−1.256414715	−6.056331748	0.405333658
H	−2.202926510	−0.785994529	−3.824662323
H	−1.687412439	−3.192724204	−3.468112856
H	−1.984298571	−4.260089128	4.829719905
H	−1.493571449	−5.733863876	2.889804863
C	−2.763288987	1.067925167	−1.881114704
C	−3.030884880	1.888466250	−0.796642251
C	−3.035745351	1.379465252	0.544380124
H	−2.768302129	1.481900632	−2.899522878
H	−3.249380116	2.954148706	−0.955374178
C	−3.294611527	2.207230255	1.686368062
C	−3.271801830	1.687451341	2.970963214
C	−2.980638495	0.304236483	3.212237472
H	−3.516709849	3.271590499	1.525292695
H	−3.477693966	2.339237983	3.831854746
C	−2.628734181	−1.594644742	4.733108160
H	−2.598740485	−2.004630907	5.752447790
C	−2.932451759	−0.257760519	4.529973400
H	−3.143156615	0.395434995	5.388515588
Li	−0.254303965	−1.385313859	0.456845040
O	0.473028598	0.350633212	0.675310653
C	0.312102573	1.527841271	0.726400841
O	0.180673181	2.680841782	0.778433031

$2CO_2@Li^+@coronene$			
C	−1.972051583	−2.167174677	−2.856727906
C	−2.080434902	−1.177158526	−1.826494693
C	−2.197224572	−1.615715050	−0.458691499
C	−2.216202738	−3.023487385	−0.148951441
C	−2.102770362	−3.988413150	−1.206521254
C	−1.980200764	−3.520799328	−2.556394516
C	−2.295271951	−0.642088162	0.606567776
C	−2.346596460	−3.457766166	1.215969772
C	−2.445931626	−2.489644241	2.275253412
C	−2.414044914	−1.083361865	1.973959224
C	−2.564796607	−2.926328438	3.638116075
C	−2.596706453	−4.333247598	3.909829521
C	−2.500442369	−5.265826782	2.889890646
C	−2.365698850	−4.860981720	1.521443035
C	−2.249814979	−5.801154398	0.444954100

C	−2.121632962	−5.382086253	−0.869895278
H	−2.037167228	−6.121516498	−1.678967055
H	−2.268094056	−6.875016453	0.680761955
H	−1.888443684	−1.833669011	−3.901051776
H	−1.898117261	−4.262105204	−3.364094597
H	−2.696775409	−4.665300659	4.952730967
H	−2.523831268	−6.340236106	3.121209261
C	−2.082142562	0.232577619	−2.097219428
C	−2.172520406	1.167186045	−1.074932037
C	−2.272530792	0.766181390	0.299905773
H	−2.015366253	0.566932339	−3.142514774
H	−2.177298438	2.241071874	−1.311499715
C	−2.358722864	1.703978706	1.380219074
C	−2.465267125	1.277684877	2.695287807
C	−2.496893041	−0.115862486	3.031667886
H	−2.348550604	2.778666186	1.147485730
H	−2.534978086	2.016001846	3.506967323
C	−2.647637286	−1.937308729	4.672884361
H	−2.744189739	−2.274887304	5.714666066
C	−2.613627081	−0.582672810	4.382277218
H	−2.682232591	0.157299310	5.192268157
Li	−0.011130140	−1.119618157	0.264856936
C	1.012494030	0.885804864	−1.663030074
O	1.039206050	1.702592588	−2.491195990
O	1.028361481	0.053844240	−0.816171794
O	0.769521547	−2.348465134	1.484426392
C	0.712309337	−3.224208833	2.283872929
O	0.686053290	−4.081246310	3.069445773
	**$3CO_2$@Li^+@coronene**		
C	−2.019623783	−2.217608405	−2.876765661
C	−2.111122702	−1.231271776	−1.840798811
C	−2.224574278	−1.671146613	−0.477320872
C	−2.263278073	−3.075466976	−0.172375793
C	−2.175768343	−4.040538602	−1.232476322
C	−2.047877810	−3.571956640	−2.581608116
C	−2.301040257	−0.700949964	0.587799012
C	−2.387850207	−3.512989072	1.191920518
C	−2.460331401	−2.547962131	2.255179593
C	−2.427705541	−1.142261259	1.955766102
C	−2.570270781	−2.986113922	3.618404788
C	−2.608973644	−4.393257573	3.888109300
C	−2.545045342	−5.323450310	2.863163225
C	−2.432875751	−4.916254611	1.493378787
C	−2.355294550	−5.855703777	0.413518184
C	−2.226994613	−5.434333292	−0.900309863
H	−2.165820545	−6.172555772	−1.712662597
H	−2.394010405	−6.929396682	0.646758761
H	−1.933704339	−1.880476835	−3.919796750
H	−1.982574747	−4.312387362	−3.391761123

H	−2.699019403	−4.727272927	4.931477823
H	−2.583222615	−6.398262256	3.091124692
C	−2.098696341	0.178123782	−2.111346628
C	−2.166984842	1.112378192	−1.087987170
C	−2.268690224	0.708366509	0.284230677
H	−2.035210565	0.512173501	−3.156582269
H	−2.163387487	2.186949203	−1.321589140
C	−2.363609620	1.645460542	1.365400433
C	−2.492626725	1.220370285	2.679633564
C	−2.518169485	−0.175295797	3.015754160
H	−2.354168587	2.720276589	1.132476672
H	−2.581568154	1.958374624	3.489826711
C	−2.644973666	−1.999292799	4.655419867
H	−2.732913103	−2.338307399	5.697584842
C	−2.623437081	−0.643290965	4.366940143
H	−2.693100890	0.095219878	5.178235770
Li	0.214141774	−0.838351296	0.874483392
C	0.952065510	0.188350730	−1.904365891
O	1.014922181	0.541099304	−3.012618692
O	0.940079376	−0.174201519	−0.775960768
O	0.784393566	−2.663822618	1.063734499
C	0.781516923	−3.849387448	1.052394580
O	0.813852477	−5.012694210	1.040065600
O	0.699119516	0.193955787	2.420365888
C	0.494466736	0.744484055	3.450858900
O	0.329736732	1.295023669	4.463422923
	**$4CO_2$@Li^+@coronene**		
C	−2.122342022	−1.238055754	−2.757363194
C	−2.388516976	−0.364855377	−1.652413283
C	−2.413823363	−0.917552714	−0.325604857
C	−2.155645083	−2.320175690	−0.124480275
C	−1.871530029	−3.166251390	−1.251172098
C	−1.878015652	−2.590087953	−2.564649277
C	−2.687339960	−0.069119552	0.804640874
C	−2.171759415	−2.870574282	1.204610719
C	−2.445817975	−2.021541238	2.331371923
C	−2.704127967	−0.621700621	2.131269669
C	−2.439929497	−2.567627046	3.659373185
C	−2.163299063	−3.963860773	3.828875655
C	−1.901967781	−4.783785543	2.742570393
C	−1.896366892	−4.265838627	1.405432361
C	−1.604010645	−5.083091091	0.263236477
C	−1.591184388	−4.552663099	−1.018057643
H	−1.373148469	−5.198615959	−1.880792647
H	−1.389167564	−6.149763000	0.420368971
H	−2.127116630	−0.817410053	−3.773209644
H	−1.682935237	−3.243236620	−3.426734780
H	−2.166480358	−4.381824276	4.845822812

H	−1.690982913	−5.851582147	2.896631832
C	−2.643900076	1.035383144	−1.819180352
C	−2.909709583	1.852922791	−0.731163948
C	−2.931478338	1.332481024	0.605610014
H	−2.639212725	1.454625941	−2.835692739
H	−3.111264846	2.922936891	−0.882877937
C	−3.178065997	2.159102242	1.750799667
C	−3.188597687	1.627224925	3.030693894
C	−2.955968449	0.230807584	3.259071957
H	−3.365993552	3.231072046	1.595501730
H	−3.385911275	2.276518957	3.895646956
C	−2.708308141	−1.695100497	4.764510465
H	−2.713383227	−2.117503259	5.779359340
C	−2.959066716	−0.345972319	4.571656406
H	−3.161484382	0.306368554	5.432687295
Li	0.366763189	−0.919021260	0.612313674
C	1.120203104	−2.072751193	−2.197031953
O	1.284969718	−2.739889731	−3.139685555
O	0.999015157	−1.387537867	−1.239674632
O	1.037326747	−2.779945654	1.215001582
C	1.176757197	−3.900059106	1.570054850
O	1.352300364	−4.998125432	1.921915872
O	0.361927783	1.029578075	0.091353190
C	0.094319400	2.180225938	0.172841509
O	−0.133819517	3.320801352	0.255881870
O	0.436356185	−0.339858083	2.567295185
C	0.340504071	−0.060305107	3.713765164
O	0.267227523	0.223402223	4.841703187

**$5CO_2@Li^+@$coronene**			
C	−2.049649699	−2.131256989	−2.775560279
C	−2.157430089	−1.117836529	−1.767951026
C	−2.250866207	−1.520269437	−0.391677202
C	−2.222138439	−2.914767538	−0.044255825
C	−2.100886530	−3.907208396	−1.074928610
C	−2.031867235	−3.476728250	−2.441777590
C	−2.381827424	−0.523975336	0.641188216
C	−2.318634211	−3.315131920	1.333126092
C	−2.469094224	−2.324014287	2.363631403
C	−2.506234463	−0.928276119	2.019843558
C	−2.591313993	−2.726330365	3.736303442
C	−2.518552349	−4.122209184	4.054355489
C	−2.355345708	−5.076729256	3.062916136
C	−2.265609405	−4.707652938	1.680282547
C	−2.124067592	−5.674425046	0.631231276
C	−2.049947563	−5.289944806	−0.698358825
H	−1.948131276	−6.048567574	−1.487261681
H	−2.079053549	−6.739520214	0.900038412
H	−1.990728754	−1.822381604	−3.829009593

H	−1.959946199	−4.237500516	−3.231519356
H	−2.599073230	−4.427425840	5.107009624
H	−2.305636021	−6.142645563	3.327340301
C	−2.176953709	0.283122756	−2.078762324
C	−2.284623678	1.242936005	−1.083875881
C	−2.399852859	0.874202722	0.296930519
H	−2.109381411	0.588503779	−3.132725848
H	−2.305617655	2.310052949	−1.347532378
C	−2.556771552	1.838986273	1.346048658
C	−2.699530748	1.448786587	2.669638507
C	−2.672893333	0.063962184	3.046828833
H	−2.584045418	2.905510263	1.079719950
H	−2.840519882	2.205035626	3.455223937
C	−2.778335386	−1.715558876	4.736977665
H	−2.895050068	−2.028295111	5.784022890
C	−2.811343529	−0.369771297	4.406385673
H	−2.955815300	0.387742920	5.189789444
Li	0.305751902	−0.672885525	1.132803175
C	0.909584650	−0.327791501	−1.813019088
O	0.985418239	−0.444277205	−2.969916594
O	0.865970326	−0.188360753	−0.638442548
O	0.798858295	−2.480057919	1.506658859
C	0.901728101	−3.652351259	1.395092442
O	1.024686299	−4.807638868	1.304628684
O	0.569346689	0.720860480	2.405207676
C	0.315818598	1.159645077	3.476974502
O	0.098781346	1.613931530	4.526898055
O	1.187321918	−3.175943147	−1.378232595
C	1.005418249	−4.096656749	−2.097435855
O	0.835875410	−5.014314371	−2.803363206
O	−0.073707993	−2.822963488	6.179203575
C	0.244106477	−2.188171234	5.247419093
O	0.577955550	−1.556601154	4.306943160
	**$6CO_2$@Li^+@coronene**		
C	−2.029363724	−2.017055358	−2.768778700
C	−2.159174590	−1.015728336	−1.751354218
C	−2.245239578	−1.431686811	−0.378380858
C	−2.196702756	−2.828537981	−0.046085710
C	−2.054954066	−3.808591447	−1.085885301
C	−1.979643027	−3.364345154	−2.447272262
C	−2.393539128	−0.449461317	0.663817980
C	−2.299164962	−3.245088439	1.325401862
C	−2.463074615	−2.266822936	2.364924569
C	−2.511439547	−0.869481088	2.036234245
C	−2.587853811	−2.686945520	3.732407688
C	−2.524430300	−4.087130251	4.033237954
C	−2.351974244	−5.028726959	3.031508677
C	−2.243410971	−4.641168024	1.656594082

C	−2.080597957	−5.594559065	0.599849760
C	−1.995109902	−5.194961134	−0.724581016
H	−1.878082948	−5.943648133	−1.520447918
H	−2.033655234	−6.662607709	0.856764304
H	−1.972094220	−1.697647374	−3.818919195
H	−1.883526520	−4.116340326	−3.243118703
H	−2.617158529	−4.405836578	5.080645345
H	−2.306672180	−6.098138318	3.282258644
C	−2.212521325	0.386075968	−2.050720725
C	−2.349097826	1.334455123	−1.048393501
C	−2.448039895	0.951645281	0.332000063
H	−2.156632046	0.701667984	−3.102761740
H	−2.413647982	2.401232743	−1.307475174
C	−2.624195220	1.903081696	1.393037100
C	−2.745333659	1.495159442	2.713032002
C	−2.693088086	0.107558342	3.074241439
H	−2.690478032	2.971035854	1.139836681
H	−2.899781484	2.240774939	3.506754686
C	−2.771948469	−1.689871358	4.745996605
H	−2.877236650	−2.015289608	5.790447842
C	−2.822712265	−0.341504790	4.429448871
H	−2.969972423	0.405192198	5.222459131
Li	0.442071569	0.124450350	0.801602823
C	0.983204469	−0.645757744	−2.190573361
O	1.018110290	−0.997599694	−3.302390830
O	0.970123502	−0.259295191	−1.074165712
O	0.708756615	−1.753013164	1.310609509
C	0.825323910	−2.906579462	1.535553250
O	0.953546617	−4.043180841	1.763434141
O	0.528641061	0.699159171	2.719990201
C	0.342345376	0.746613393	3.886755615
O	0.173027721	0.825723122	5.038141685
O	1.279895830	−3.309879557	−1.242714489
C	1.063986986	−4.275593893	−1.888046096
O	0.855749652	−5.232745491	−2.529498796
O	−0.107593008	−3.171367175	6.202931288
C	0.279462567	−2.639735621	5.234059656
O	0.676678612	−2.102015525	4.260062751
O	0.676179173	2.164881876	0.320030316
C	0.130757765	3.189111180	0.094492557
O	−0.402980531	4.202971407	−0.131875150

	**$7CO_2$@Li^+@coronene**		
C	−2.084120421	−1.243998811	−2.713531866
C	−2.330023903	−0.380159134	−1.596736866
C	−2.359759152	−0.949505604	−0.276573274
C	−2.134985411	−2.359759265	−0.095474712
C	−1.881556966	−3.197617256	−1.234360404
C	−1.873278405	−2.604058529	−2.539225611

C	−2.624733467	−0.112224207	0.863915070
C	−2.174305076	−2.929980725	1.224004480
C	−2.444572768	−2.093689353	2.360744250
C	−2.667864162	−0.685766204	2.181467748
C	−2.495774102	−2.665686604	3.676552161
C	−2.270794974	−4.074390351	3.824809882
C	−1.995635146	−4.878099581	2.730092853
C	−1.943076960	−4.335861175	1.404307521
C	−1.673019782	−5.144448581	0.251096506
C	−1.650046817	−4.595761342	−1.022243461
H	−1.467686762	−5.239005360	−1.894899743
H	−1.492485158	−6.220174203	0.389685756
H	−2.079446187	−0.810133390	−3.723848250
H	−1.696246343	−3.249081001	−3.411420773
H	−2.321499347	−4.513330227	4.831011431
H	−1.820219719	−5.954770646	2.867135475
C	−2.571022159	1.024485047	−1.745496065
C	−2.831572768	1.830499956	−0.647125565
C	−2.858792894	1.293124871	0.682140096
H	−2.563747684	1.457385650	−2.756331438
H	−3.027461909	2.903073469	−0.787035947
C	−3.115794114	2.103151569	1.836146106
C	−3.148149873	1.552100844	3.108195403
C	−2.933666155	0.150170987	3.318479065
H	−3.301185706	3.178017513	1.697836568
H	−3.352870403	2.191743874	3.978430000
C	−2.768475072	−1.807843904	4.792345539
H	−2.811099400	−2.248641988	5.797971660
C	−2.977611682	−0.449027532	4.619584118
H	−3.184639319	0.191865066	5.488013320
Li	0.459361036	−0.790247549	0.568360210
C	1.129814409	−1.988482323	−2.271280124
O	1.291968910	−2.571563595	−3.269050205
O	0.993884741	−1.374693211	−1.270652715
O	1.017961568	−2.577284362	1.278491255
C	1.156389459	−3.667035722	1.709143518
O	1.307040763	−4.743309731	2.135281829
O	0.121415953	2.857654242	2.689394045
C	−0.611368053	3.680046079	3.115903489
O	−1.338573347	4.495980690	3.537403939
O	1.741542817	−4.517029940	−0.938172962
C	1.380626054	−5.614224985	−1.184961571
O	1.018963899	−6.701864815	−1.427381912
O	−0.091080204	−3.374365469	6.313322904
C	0.365492042	−2.953317398	5.320273136
O	0.825615092	−2.524681481	4.320631893
O	0.451352086	1.127291904	−0.074412506
C	0.192997673	2.276870151	−0.174633470
O	−0.033402794	3.415552208	−0.292336982

O	0.469683005	−0.114043521	2.453604292
C	0.311137130	0.182688041	3.585588845
O	0.165030226	0.479823212	4.704318887
	**$8CO_2@Li^+@$coronene**		
C	−2.069468578	−1.200575927	−2.757258369
C	−2.318875127	−0.343111040	−1.635983951
C	−2.348445307	−0.919303994	−0.319642011
C	−2.123739770	−2.329787868	−0.146078304
C	−1.864279618	−3.161142654	−1.288517046
C	−1.849534816	−2.559951524	−2.589070576
C	−2.611681034	−0.087985809	0.825014537
C	−2.161539020	−2.906967242	1.170315400
C	−2.429482532	−2.076716530	2.312521063
C	−2.653776540	−0.667902031	2.139749340
C	−2.472091452	−2.654649739	3.626256185
C	−2.246514062	−4.063889775	3.766412956
C	−1.978901693	−4.862970464	2.666195628
C	−1.929125519	−4.313910508	1.343047114
C	−1.659856360	−5.116506368	0.185903004
C	−1.633360419	−4.560314690	−1.084018589
H	−1.445822891	−5.197953190	−1.959813963
H	−1.480615435	−6.193104059	0.318768623
H	−2.069084817	−0.764298695	−3.766199937
H	−1.663712638	−3.198419470	−3.464338420
H	−2.294502939	−4.508162848	4.770425081
H	−1.804728322	−5.940564610	2.797184681
C	−2.556804055	1.063479366	−1.776374737
C	−2.803544694	1.865799457	−0.672380530
C	−2.836067478	1.320172878	0.652593950
H	−2.557430448	1.502360675	−2.784390984
H	−2.989363799	2.940992192	−0.806285465
C	−3.092267240	2.124328031	1.810808834
C	−3.127704323	1.566238588	3.079619501
C	−2.913961014	0.163279115	3.281874636
H	−3.274057490	3.200545144	1.677524521
H	−3.332270865	2.200722945	3.953630833
C	−2.741868938	−1.801695240	4.746589632
H	−2.784325858	−2.246496656	5.750469480
C	−2.953083210	−0.442132019	4.580232390
H	−3.158141556	0.194630278	5.452164153
Li	0.481872699	−0.872056861	0.816501921
C	1.169034312	−1.867407797	−2.097013275
O	1.377292728	−2.265124466	−3.174659915
O	0.976234620	−1.454469722	−1.008960528
O	1.061304070	−2.647343718	1.529210127
C	1.177257930	−3.766445759	1.883029595
O	1.308392383	−4.872066723	2.234982246
O	0.134059691	2.816150058	2.702866768

C	−0.582186869	3.642910440	3.148641247
O	−1.292810483	4.463068674	3.590154750
O	1.736309988	−4.471053701	−0.869455421
C	1.402902937	−5.580484700	−1.099865558
O	1.068778888	−6.680583069	−1.325724217
O	−0.230439282	−3.486194358	6.457184656
C	0.305111300	−3.047792715	5.511689780
O	0.841746818	−2.603244560	4.558498811
O	0.489801101	1.018438209	0.184263036
C	0.314232433	2.142933339	−0.129953832
O	0.159692712	3.256656287	−0.442993976
O	0.454044688	−0.203443778	2.693472312
C	0.306144904	0.153727276	3.808566445
O	0.170825840	0.511255966	4.911230675
O	−0.553689030	1.387444669	−4.715548398
C	0.139996941	1.190282848	−3.790847117
O	0.825768856	0.983908097	−2.852911875
	**$9CO_2@Li^+@coronene$**		
C	−2.167324026	−1.222394043	−2.809706601
C	−2.385205134	−0.366778264	−1.680760099
C	−2.381364039	−0.944126365	−0.365374110
C	−2.165239877	−2.355111352	−0.200196163
C	−1.941583831	−3.186429222	−1.349652587
C	−1.951590926	−2.583177925	−2.649273706
C	−2.607338023	−0.113180330	0.785449636
C	−2.176805827	−2.934565652	1.114006254
C	−2.400776759	−2.103743834	2.264101683
C	−2.616042476	−0.693241618	2.099768413
C	−2.413700083	−2.683357377	3.577657687
C	−2.196191326	−4.094310962	3.710983021
C	−1.972376688	−4.894752544	2.601775037
C	−1.956283454	−4.344002291	1.278461080
C	−1.723419306	−5.147562031	0.114238129
C	−1.719807849	−4.588489237	−1.154596114
H	−1.557286416	−5.225325348	−2.035971832
H	−1.552622603	−6.226247912	0.241727865
H	−2.188281428	−0.784076791	−3.817554711
H	−1.790385058	−3.222010002	−3.529217468
H	−2.217683992	−4.539575397	4.715612286
H	−1.804845985	−5.974273812	2.726155315
C	−2.626789509	1.039708246	−1.814449513
C	−2.845490600	1.841249671	−0.704289648
C	−2.836928165	1.294914131	0.620563818
H	−2.656681898	1.478560354	−2.822087792
H	−3.036745303	2.916293970	−0.831321575
C	−3.055852553	2.099808765	1.785841275
C	−3.057164897	1.541570122	3.055133989
C	−2.845909592	0.137050907	3.249128998

H	−3.240383970	3.176125770	1.657320045
H	−3.236175328	2.175947535	3.934873855
C	−2.652125945	−1.831362160	4.705884882
H	−2.675515718	−2.278475786	5.709312429
C	−2.860588458	−0.470681149	4.546958954
H	−3.043149778	0.165554997	5.424219122
Li	0.809689910	−0.786376550	0.794639105
C	1.070027022	−1.870447772	−2.151005875
O	1.246409165	−2.240287849	−3.244274592
O	0.909480601	−1.494775442	−1.044092970
O	0.905946386	−2.658363830	1.561889428
C	1.083316616	−3.777437420	1.889809615
O	1.273953348	−4.881555606	2.219656477
O	0.202812861	2.918519168	2.619368396
C	−0.556117873	3.716385207	3.047425818
O	−1.307576297	4.508353638	3.472320790
O	1.677818658	−4.446495569	−0.879496857
C	1.338327967	−5.544748080	−1.151448945
O	1.003106002	−6.634855369	−1.419995672
O	−0.164560117	−3.466103899	6.416767771
C	0.379972428	−2.980772160	5.499823700
O	0.927442960	−2.491068534	4.575008246
O	0.450805289	1.056001197	0.141218654
C	0.230969979	2.159395225	−0.212316079
O	0.042196313	3.258217221	−0.560086780
O	0.526550367	−0.107515347	2.746754957
C	0.293539152	0.288303352	3.833622611
O	0.090592364	0.685301673	4.913021140
O	−0.640218147	1.350594637	−4.750757298
C	0.055191433	1.165093448	−3.825192781
O	0.743933632	0.971462819	−2.886531708
O	2.901834685	−0.514542935	1.077500372
C	3.556823418	−0.327790247	2.045708636
O	4.199677552	−0.142690054	3.003078058
	**$10CO_2@Li^+@coronene$**		
C	−2.255718711	−1.175784947	−2.796023351
C	−2.463700879	−0.322207384	−1.663521413
C	−2.399286459	−0.893571183	−0.346696770
C	−2.128369864	−2.295622216	−0.183822071
C	−1.911435674	−3.123664562	−1.337260444
C	−1.987679054	−2.527453383	−2.638165160
C	−2.621080896	−0.066919493	0.807932579
C	−2.085117442	−2.870795582	1.131871721
C	−2.317347306	−2.046217526	2.284929305
C	−2.581253224	−0.643780121	2.123031098
C	−2.289599854	−2.624413536	3.598654895
C	−2.022930372	−4.027030635	3.728502777
C	−1.784330217	−4.820076029	2.617076445

C	−1.810604842	−4.271296228	1.293449106
C	−1.576566130	−5.068866325	0.125447757
C	−1.630548733	−4.515251906	−1.144648293
H	−1.469973864	−5.149015186	−2.028614111
H	−1.364105686	−6.140354636	0.250415857
H	−2.331010442	−0.744162826	−3.804147256
H	−1.836876377	−3.165227746	−3.520717764
H	−2.014538722	−4.473012779	4.733058192
H	−1.576302699	−5.892558417	2.740379014
C	−2.756109606	1.074780384	−1.794015129
C	−2.964211140	1.873269546	−0.679082399
C	−2.896489586	1.333313641	0.646417456
H	−2.832635212	1.509201280	−2.801081098
H	−3.191811522	2.941428246	−0.803334958
C	−3.103994358	2.135653928	1.815518753
C	−3.056591245	1.580866973	3.085512882
C	−2.806425401	0.182320544	3.275946991
H	−3.320153645	3.206489202	1.690802047
H	−3.225671301	2.213061185	3.968637063
C	−2.529497030	−1.777580958	4.730491061
H	−2.520896569	−2.223777496	5.734750649
C	−2.774534455	−0.422732810	4.574738482
H	−2.953172429	0.210325722	5.455173324
Li	0.839798716	−0.746788307	0.620005453
C	1.035926435	−1.869217953	−2.310198705
O	1.176383382	−2.310386013	−3.381905773
O	0.913953079	−1.419004892	−1.226181720
O	1.073283999	−2.541785036	1.400516376
C	1.281786126	−3.635914959	1.791091436
O	1.481034150	−4.718334037	2.178163404
O	0.203377902	3.017478392	2.305433835
C	−0.514363102	3.761419333	2.877576150
O	−1.224814529	4.499710910	3.445203355
O	1.780968437	−4.371525322	−0.968728361
C	1.442352302	−5.471108084	−1.236113293
O	1.106240842	−6.561945609	−1.500893780
O	0.058526959	−3.386606929	6.367493406
C	0.507713397	−2.844238117	5.431954238
O	0.962080077	−2.297428256	4.488842093
O	0.369626945	1.158418170	−0.090055021
C	0.103506718	2.233530021	−0.497011534
O	−0.123410260	3.305165645	−0.902976157
O	0.509638931	−0.010185402	2.501882654
C	0.356490151	0.427776451	3.586004087
O	0.216543031	0.870288835	4.657418729
O	−0.924526784	1.420590915	−4.840437291
C	−0.152245327	1.191407482	−3.988723797
O	0.614382716	0.956209079	−3.122486891
O	3.720690601	−2.550352111	3.126270080

C	3.557100391	−1.425906692	3.424122839
O	3.386912852	−0.294389586	3.704042010
O	2.872153976	−0.070462188	0.713774399
C	3.263288866	1.018466954	0.968938830
O	3.643016740	2.095305183	1.209773826

	**$11CO_2$@Li^+@coronene**		
C	−2.269911107	−1.197337168	−2.795375819
C	−2.488150727	−0.337675905	−1.669637661
C	−2.413437791	−0.897006274	−0.348335723
C	−2.122397446	−2.293688668	−0.174853130
C	−1.897242434	−3.128256359	−1.321682551
C	−1.982860668	−2.543800373	−2.627200726
C	−2.641426110	−0.063728625	0.800456437
C	−2.070608824	−2.857840157	1.145556884
C	−2.312983984	−2.027644518	2.292544416
C	−2.589823500	−0.628870396	2.120080222
C	−2.284199528	−2.596767594	3.609935036
C	−2.008958945	−3.996937379	3.750147524
C	−1.758435898	−4.794698508	2.645015889
C	−1.783872602	−4.254549752	1.317743613
C	−1.543007873	−5.058999682	0.155836497
C	−1.602026874	−4.515509607	−1.118518984
H	−1.439288030	−5.155324478	−1.997777138
H	−1.322106902	−6.127569820	0.289473130
H	−2.350491995	−0.774758459	−3.806790560
H	−1.823774279	−3.186028866	−3.505027477
H	−2.001882012	−4.436192697	4.757569144
H	−1.541759996	−5.864540565	2.775664777
C	−2.796604396	1.054926308	−1.810598069
C	−3.010850323	1.860028324	−0.701587723
C	−2.932292358	1.331897365	0.628267248
H	−2.878056305	1.480355318	−2.821262492
H	−3.251004795	2.924444835	−0.834015805
C	−3.137162116	2.143027425	1.791782899
C	−3.077203969	1.600029706	3.066243855
C	−2.816287602	0.204721276	3.267507371
H	−3.361857404	3.211128686	1.659005557
H	−3.245716215	2.238540767	3.944995859
C	−2.528333108	−1.743214221	4.735690969
H	−2.517721645	−2.182583986	5.743067260
C	−2.778033055	−0.390265717	4.570713381
H	−2.958820739	0.248205595	5.446845186
Li	0.838694871	−0.654500947	0.575648835
C	1.041195930	−1.813546580	−2.339774937
O	1.160017192	−2.190757964	−3.437166346
O	0.927599274	−1.436364683	−1.226522731
O	1.177428194	−2.481198157	1.413980919
C	1.323794900	−3.611586599	1.721196961

O	1.468939801	−4.726813357	2.033026844
O	0.206960103	3.033865370	2.266305182
C	−0.518365171	3.779712883	2.826676454
O	−1.236387335	4.518774425	3.383455014
O	1.814523859	−4.372962204	−1.057712849
C	1.461765253	−5.485559914	−1.241064447
O	1.112011847	−6.587956523	−1.423895592
O	0.096994846	−3.380484942	6.350941229
C	0.512415984	−2.814807595	5.413524366
O	0.930212499	−2.243449869	4.468273642
O	0.264467011	1.158477136	−0.152857069
C	0.030029066	2.245074611	−0.546314597
O	−0.170576584	3.327882350	−0.936709303
O	0.502068258	0.037456824	2.463701635
C	0.372343329	0.448303992	3.561124373
O	0.257986819	0.868636312	4.644671446
O	−0.944122123	1.323960902	−4.899944860
C	−0.177564954	1.156644449	−4.028811315
O	0.582665280	0.983174272	−3.142732317
O	3.731097980	−2.970217500	3.634811772
C	3.635839356	−1.807022530	3.517496364
O	3.529417116	−0.638587142	3.399906322
O	2.824589526	0.099200725	0.600355547
C	3.320746386	1.037760992	1.126349154
O	3.802547801	1.974632909	1.626926533
O	3.925781520	−2.068397555	−1.623684217
C	4.038901127	−2.443790382	−0.515897101
O	4.142753986	−2.807446560	0.598935930

	**$12CO_2@Li^+@$coronene**		
C	−2.404170679	−1.260915477	−2.925834888
C	−2.652750143	−0.379482467	−1.823320377
C	−2.543804943	−0.895115892	−0.487201946
C	−2.180131921	−2.267923675	−0.275162372
C	−1.937414898	−3.128407126	−1.398906142
C	−2.062833697	−2.589259419	−2.720392690
C	−2.789173124	−0.035533231	0.636827870
C	−2.046749875	−2.777997361	1.060777371
C	−2.278800270	−1.914723227	2.185511318
C	−2.651258503	−0.543499007	1.972738986
C	−2.143527101	−2.423827292	3.521488660
C	−1.787340554	−3.800491454	3.700891790
C	−1.561103592	−4.633232310	2.616327002
C	−1.678560869	−4.150278968	1.271856453
C	−1.440575190	−4.985940898	0.131776983
C	−1.566276650	−4.491137856	−1.157404299
H	−1.394666745	−5.152286210	−2.019155754
H	−1.160133800	−6.035910676	0.295545908
H	−2.497120545	−0.867670485	−3.947778142

H	−1.878790765	−3.249069095	−3.580213430
H	−1.713063192	−4.198577890	4.722872625
H	−1.290127535	−5.686361160	2.777014544
C	−3.016062397	0.995730345	−2.004232323
C	−3.260536050	1.823096615	−0.918971295
C	−3.152648778	1.337562673	0.425232197
H	−3.114718300	1.386513434	−3.026923028
H	−3.543255556	2.872953238	−1.080290757
C	−3.374265267	2.177377547	1.565392881
C	−3.242342414	1.685750720	2.855035211
C	−2.878443216	0.321239727	3.096644819
H	−3.655982846	3.227198424	1.401861620
H	−3.427932514	2.343767937	3.716012035
C	−2.380735778	−1.538551187	4.623245312
H	−2.290792604	−1.930351738	5.646073820
C	−2.733085509	−0.213115554	4.417894965
H	−2.913325951	0.447649428	5.277678753
Li	0.841030606	−0.632223460	1.003041384
C	0.953469132	−1.762712888	−1.980941572
O	1.107014741	−2.006671216	−3.113893460
O	0.816510372	−1.507085321	−0.838790631
O	1.226056348	−2.541980576	1.766823250
C	1.424147802	−3.704639032	1.797835048
O	1.622522676	−4.855355540	1.836415109
O	−0.038279300	2.912827804	2.736103959
C	−0.811043395	3.771534548	2.982140766
O	−1.574433737	4.626779406	3.224386324
O	1.902489254	−4.317509445	−1.145841390
C	1.541868501	−5.428476177	−1.322693961
O	1.184987552	−6.529521327	−1.499170972
O	−0.059351462	−3.217303559	6.730037616
C	0.542808993	−2.798984230	5.815363808
O	1.137614099	−2.377588369	4.887770167
O	0.035585142	1.040213026	0.233065144
C	−0.152235886	2.163081953	−0.071756972
O	−0.332369921	3.276170601	−0.379130752
O	0.428390962	−0.089720329	2.867381652
C	0.409473719	0.306073597	3.977189306
O	0.400176100	0.700763648	5.076616427
O	−0.758263885	1.271160781	−4.631469826
C	−0.071364895	1.159984941	−3.689803908
O	0.615822266	1.045501441	−2.735286997
O	3.820123491	−3.141142249	3.574694135
C	3.712539886	−1.971902847	3.581312956
O	3.599096843	−0.799040889	3.572913293
O	2.675540186	0.237020962	0.917208825
C	3.852796004	0.324982370	0.861659426
O	5.012921341	0.434538747	0.805282188
O	4.868797473	−3.266098877	−1.621311102

C	4.416988557	−2.892082130	−0.607624451
O	3.960463915	−2.513396977	0.413555000
O	3.535639037	−0.346745834	−1.993330615
C	3.508891333	0.085856378	−3.092790273
O	3.470420835	0.514987872	−4.181287668
O	1.453064000	5.553011000	2.310000000
O	3.511672000	3.301534000	−2.357570000
O	4.747454000	−1.320673000	2.551780000
O	5.571283000	1.472618000	−2.202134000
O	3.685930000	−3.913454000	2.549466000
O	1.456607000	−4.504595000	−2.536882000
C	2.009133000	4.967242000	1.171074000
C	1.309577000	5.122599000	−0.046078000
C	1.859289000	4.524824000	−1.191829000
H	1.343811000	4.613619000	−2.157908000
C	3.079165000	3.813539000	−1.164818000
C	3.798469000	3.704431000	0.056302000
C	3.230083000	4.277780000	1.214036000
H	3.777491000	4.206927000	2.168282000
C	5.166943000	3.033739000	0.136853000
H	5.798125000	3.376180000	−0.707339000
H	5.665659000	3.385244000	1.060637000
C	5.149747000	1.509073000	0.153427000
C	5.364365000	0.760398000	−1.022646000
C	5.382508000	−0.644403000	−0.997120000
H	5.568113000	−1.201940000	−1.928001000
C	5.177369000	−1.362179000	0.195375000
C	4.937736000	−0.612646000	1.371442000
C	4.928220000	0.791773000	1.346297000
H	4.752298000	1.351135000	2.278997000
C	5.191759000	−2.883435000	0.211149000
H	5.796273000	−3.230589000	−0.649216000
H	5.702853000	−3.238260000	1.127570000
C	3.814020000	−3.537830000	0.125621000
C	3.185890000	−3.699024000	−1.123937000
H	3.656525000	−3.303931000	−2.035625000
C	1.963638000	−4.386429000	−1.268872000
C	1.360674000	−4.972670000	−0.121180000
C	1.963181000	−4.764617000	1.133895000
H	1.507971000	−5.187830000	2.040448000

C	3.162763000	−4.041461000	1.282804000
C	0.101073000	−5.829298000	−0.226438000
H	0.113678000	−6.382533000	−1.185900000
H	0.130684000	−6.586070000	0.581079000
H	5.594631000	0.851578000	−2.955734000
H	4.161714000	−3.053550000	2.605247000
H	4.325062000	2.749993000	−2.262424000
H	1.997435000	5.327133000	3.087910000
H	4.492080000	−0.706444000	3.265959000
H	0.492733000	−4.711355000	−2.511267000
O	−1.304424000	−4.833483000	−2.493660000
O	−3.464479000	−3.884676000	2.559873000
O	−5.396234000	1.370783000	−2.196897000
O	−4.729921000	−1.428552000	2.577373000
O	−3.665102000	3.571191000	−2.389412000
O	−1.276661000	5.021533000	2.445164000
C	−1.881430000	−4.570519000	−1.250111000
C	−1.208498000	−5.057835000	−0.105936000
C	−1.778021000	−4.784774000	1.148199000
H	−1.279189000	−5.129011000	2.064382000
C	−2.989445000	−4.072924000	1.290240000
C	−3.687216000	−3.641793000	0.129919000
C	−3.099283000	−3.885039000	−1.130486000
H	−3.625412000	−3.554417000	−2.040479000
C	−5.066315000	−2.991179000	0.217876000
H	−5.574349000	−3.350980000	1.134166000
H	−5.670563000	−3.342969000	−0.640827000
C	−5.065071000	−1.468301000	0.204641000
C	−4.879591000	−0.720581000	1.388850000
C	−4.879898000	0.683565000	1.367979000
H	−4.744394000	1.242361000	2.307423000
C	−5.058163000	1.403374000	0.171036000
C	−5.219907000	0.656470000	−1.016891000
C	−5.230577000	−0.748659000	−0.994510000
H	−5.384548000	−1.303960000	−1.933165000
C	−5.080510000	2.926616000	0.163518000
H	−5.569035000	3.267691000	1.096416000
H	−5.715282000	3.274406000	−0.674987000
C	−3.711763000	3.596760000	0.074114000
C	−3.039844000	3.977102000	1.251908000
H	−3.479547000	3.752302000	2.234404000
C	−1.820237000	4.683036000	1.232982000
C	−1.260234000	5.053322000	−0.020227000
C	−1.906494000	4.630757000	−1.196272000
H	−1.487218000	4.893344000	−2.177852000
C	−3.109555000	3.895881000	−1.177514000
C	0.002460000	5.905236000	−0.112456000
H	−0.007056000	6.666744000	0.692486000
H	−0.022494000	6.453670000	−1.073196000

H	−4.520892000	−0.809996000	3.303298000
H	−4.332074000	2.854150000	−2.291583000
H	−4.002801000	−3.058040000	2.593406000
H	−1.820975000	−4.386864000	−3.190843000
H	−5.431258000	0.753891000	−2.952615000
H	−0.333036000	5.292512000	2.355580000
H	−1.859235000	−0.124166000	−0.269686000
H	−2.152238000	−0.833444000	−0.277184000
H	0.086042000	2.231266000	−0.018367000
H	−0.295081000	1.903839000	0.561255000
H	2.658227000	0.952841000	−2.673768000
H	2.471991000	0.209642000	−2.622673000
		PPA–H_2–4	
O	1.502309000	5.440421000	2.405454000
O	3.495275000	3.451045000	−2.408779000
O	4.965119000	−1.253093000	2.507340000
O	5.431774000	1.492543000	−2.322474000
O	3.878079000	−3.826707000	2.598139000
O	1.440760000	−4.637621000	−2.361369000
C	2.049773000	4.929499000	1.225324000
C	1.327431000	5.143158000	0.030885000
C	1.856552000	4.608641000	−1.154949000
H	1.318164000	4.738167000	−2.103917000
C	3.080252000	3.904520000	−1.186136000
C	3.826008000	3.744197000	0.013254000
C	3.277701000	4.252120000	1.210138000
H	3.842054000	4.134171000	2.149287000
C	5.198930000	3.077411000	0.031378000
H	5.788473000	3.416547000	−0.843691000
H	5.741163000	3.433685000	0.928359000
C	5.182648000	1.553116000	0.058392000
C	5.303529000	0.793186000	−1.124450000
C	5.322798000	−0.611579000	−1.086424000
H	5.445069000	−1.177578000	−2.022742000
C	5.213187000	−1.317980000	0.125608000
C	5.063123000	−0.556950000	1.308527000
C	5.053082000	0.846914000	1.271094000
H	4.953398000	1.415555000	2.209399000
C	5.250759000	−2.839255000	0.156771000
H	5.809650000	−3.185400000	−0.734196000
H	5.823556000	−3.173138000	1.044204000
C	3.885037000	−3.523166000	0.160262000
C	3.205553000	−3.744548000	−1.052386000
H	3.630408000	−3.374621000	−1.996386000
C	1.995434000	−4.463201000	−1.116767000
C	1.450628000	−5.011013000	0.076805000
C	2.107181000	−4.747782000	1.293634000
H	1.698877000	−5.145386000	2.233245000
C	3.299780000	−4.000878000	1.362756000

C	0.189905000	−5.870978000	0.068739000
H	0.201512000	−6.543082000	−0.812439000
H	0.212704000	−6.521437000	0.964079000
H	5.354182000	0.871175000	−3.072285000
H	4.354669000	−2.965340000	2.606126000
H	4.279960000	2.855529000	−2.340040000
H	2.037490000	5.137357000	3.163428000
H	4.764915000	−0.632627000	3.233827000
H	0.516995000	−4.982169000	−2.303909000
O	−1.248882000	−5.230254000	−2.308139000
O	−3.335815000	−3.545387000	2.582409000
O	−4.860745000	1.353283000	−2.304811000
O	−5.135610000	−1.439451000	2.511347000
O	−3.747686000	3.908621000	−2.405517000
O	−1.163785000	4.667718000	2.487820000
C	−1.807634000	−4.781308000	−1.107992000
C	−1.117901000	−5.085898000	0.087190000
C	−1.671119000	−4.626977000	1.293197000
H	−1.158122000	−4.828263000	2.243577000
C	−2.887204000	−3.909423000	1.345397000
C	−3.599728000	−3.658889000	0.140571000
C	−3.027240000	−4.089816000	−1.075072000
H	−3.569173000	−3.906947000	−2.016846000
C	−4.975543000	−2.998776000	0.140489000
H	−5.547618000	−3.348346000	1.022558000
H	−5.525602000	−3.353335000	−0.752492000
C	−4.973192000	−1.474932000	0.124182000
C	−5.061010000	−0.726907000	1.317287000
C	−5.096869000	0.677629000	1.293405000
H	−5.185487000	1.233953000	2.239699000
C	−5.030803000	1.395528000	0.084885000
C	−4.915212000	0.647131000	−1.109097000
C	−4.893055000	−0.756729000	−1.085190000
H	−4.823731000	−1.314190000	−2.033026000
C	−5.058628000	2.916789000	0.069256000
H	−5.586811000	3.261431000	0.979270000
H	−5.652146000	3.264612000	−0.798831000
C	−3.685880000	3.587008000	0.029720000
C	−2.969027000	3.795838000	1.223606000
H	−3.367346000	3.425737000	2.179012000
C	−1.755718000	4.512148000	1.257382000
C	−1.243082000	5.063805000	0.052334000
C	−1.935691000	4.810102000	−1.146762000
H	−1.554147000	5.212468000	−2.095657000
C	−3.133509000	4.070205000	−1.186343000
C	0.016983000	5.924005000	0.028580000
H	0.009233000	6.622792000	0.888707000
H	−0.012241000	6.546921000	−0.885709000
H	−5.123801000	−0.817970000	3.264426000

H	−4.239816000	3.055474000	−2.402689000
H	−4.031704000	−2.847582000	2.528726000
H	−1.722643000	−4.817673000	−3.055944000
H	−4.669259000	0.740487000	−3.039973000
H	−0.261710000	5.061794000	2.418200000
H	−1.981519000	0.473784000	0.563414000
H	−1.999379000	−0.283964000	0.443297000
H	0.608531000	2.026458000	2.326632000
H	0.219833000	2.659835000	2.518233000
H	2.643656000	1.191477000	−3.093981000
H	2.388554000	0.477802000	−3.220686000
H	0.157173000	−2.836216000	−3.401212000
H	−0.299630000	−2.308551000	−3.724361000
	**PPA–H_2–5**		
O	1.519604000	5.284839000	2.351005000
O	3.530822000	3.507218000	−2.538381000
O	5.083549000	−1.310037000	2.363857000
O	5.340819000	1.447165000	−2.475083000
O	3.847413000	−3.814268000	2.466771000
O	1.374213000	−4.693192000	−2.463059000
C	2.068405000	4.826166000	1.150504000
C	1.352874000	5.097132000	−0.037036000
C	1.888261000	4.615244000	−1.242650000
H	1.355779000	4.790831000	−2.187545000
C	3.109489000	3.908875000	−1.299343000
C	3.849479000	3.692794000	−0.105436000
C	3.294795000	4.146834000	1.110166000
H	3.852474000	3.984356000	2.046499000
C	5.222948000	3.025839000	−0.109292000
H	5.797817000	3.366956000	−0.993262000
H	5.778674000	3.380583000	0.779983000
C	5.205110000	1.501407000	−0.084074000
C	5.255566000	0.745206000	−1.274793000
C	5.260432000	−0.659941000	−1.242122000
H	5.325563000	−1.224690000	−2.185138000
C	5.206165000	−1.369805000	−0.027929000
C	5.131367000	−0.611605000	1.163632000
C	5.135810000	0.792720000	1.131199000
H	5.093307000	1.357732000	2.075935000
C	5.218420000	−2.892216000	−0.002976000
H	5.754415000	−3.245235000	−0.905159000
H	5.801067000	−3.238602000	0.872978000
C	3.842187000	−3.554234000	0.022416000
C	3.152576000	−3.790263000	−1.182321000
H	3.575741000	−3.444488000	−2.136353000
C	1.936225000	−4.501314000	−1.228544000
C	1.399880000	−5.025456000	−0.019482000
C	2.062909000	−4.742371000	1.189120000
H	1.660521000	−5.123519000	2.138236000

C	3.260966000	−4.002885000	1.237848000
C	0.147779000	−5.896043000	−0.009996000
H	0.174897000	−6.594672000	−0.869556000
H	0.169018000	−6.516131000	0.906687000
H	5.188829000	0.835495000	−3.221267000
H	4.362428000	−2.975117000	2.454532000
H	4.281026000	2.866922000	−2.480752000
H	2.025622000	4.909394000	3.096430000
H	4.912966000	−0.689666000	3.098073000
H	0.453162000	−5.038642000	−2.399502000
O	−1.317314000	−5.444761000	−2.404261000
O	−3.363330000	−3.415308000	2.369115000
O	−4.684901000	1.314409000	−2.487446000
O	−5.348183000	−1.510888000	2.272381000
O	−3.702709000	3.930640000	−2.541855000
O	−1.139113000	4.503065000	2.386458000
C	−1.879510000	−4.926465000	−1.235807000
C	−1.168188000	−5.126692000	−0.031723000
C	−1.711932000	−4.581428000	1.143309000
H	−1.183527000	−4.700786000	2.098961000
C	−2.936413000	−3.878837000	1.154974000
C	−3.668474000	−3.728710000	−0.054061000
C	−3.107662000	−4.249436000	−1.239421000
H	−3.661856000	−4.139757000	−2.185763000
C	−5.037493000	−3.055831000	−0.095812000
H	−5.649705000	−3.404758000	0.759642000
H	−5.559410000	−3.395354000	−1.011186000
C	−5.011284000	−1.531550000	−0.097907000
C	−5.170259000	−0.791645000	1.092514000
C	−5.173554000	0.613501000	1.080043000
H	−5.321619000	1.163586000	2.022026000
C	−5.011754000	1.340029000	−0.113744000
C	−4.828935000	0.598956000	−1.305194000
C	−4.831680000	−0.805310000	−1.292421000
H	−4.700369000	−1.357652000	−2.236692000
C	−5.029279000	2.861074000	−0.117872000
H	−5.578460000	3.199283000	0.782275000
H	−5.601301000	3.218455000	−0.996688000
C	−3.655481000	3.528783000	−0.120327000
C	−2.946700000	3.692668000	1.084811000
H	−3.348100000	3.283185000	2.022812000
C	−1.732346000	4.404201000	1.151098000
C	−1.217423000	5.010158000	−0.027915000
C	−1.902126000	4.800621000	−1.240131000
H	−1.515819000	5.239251000	−2.170812000
C	−3.095324000	4.055870000	−1.313897000
C	0.040539000	5.874090000	−0.011774000
H	0.027157000	6.539524000	0.874454000
H	0.013476000	6.533011000	−0.900840000

H	−5.289680000	−0.901694000	3.034028000
H	−4.158081000	3.058467000	−2.575427000
H	−4.161231000	−2.838153000	2.292446000
H	−1.862064000	−5.178351000	−3.169045000
H	−4.459574000	0.705339000	−3.216000000
H	−0.241883000	4.912481000	2.335337000
H	−2.404455000	−0.367976000	3.105102000
H	−2.533378000	−1.121586000	3.030039000
H	0.917408000	2.028885000	2.829873000
H	0.395913000	2.592651000	2.802586000
H	2.586824000	1.344158000	−3.425864000
H	2.405878000	0.628734000	−3.640730000
H	2.277856000	−0.854793000	0.455176000
H	1.693847000	−0.451984000	0.746780000
H	−0.557106000	−1.703574000	−0.927072000
H	−0.644361000	−2.289638000	−0.439396000
		PPA−H_2−6	
O	1.365045000	5.364405000	2.404470000
O	3.576656000	3.314200000	−2.285607000
O	4.727568000	−1.489978000	2.483102000
O	5.621954000	1.483661000	−2.146598000
O	3.789117000	−4.092751000	2.453840000
O	1.315882000	−4.411422000	−2.543132000
C	1.953496000	4.821270000	1.261111000
C	1.288108000	5.018878000	0.030185000
C	1.875844000	4.471902000	−1.121639000
H	1.389300000	4.597729000	−2.098626000
C	3.100821000	3.769871000	−1.087049000
C	3.784882000	3.617468000	0.149465000
C	3.178496000	4.139851000	1.312373000
H	3.699801000	4.035817000	2.278011000
C	5.155888000	2.953940000	0.242580000
H	5.807274000	3.333338000	−0.570075000
H	5.626556000	3.272698000	1.192586000
C	5.148327000	1.429752000	0.200100000
C	5.387708000	0.727664000	−1.000044000
C	5.409162000	−0.677199000	−1.027775000
H	5.610212000	−1.198740000	−1.976145000
C	5.187603000	−1.439185000	0.133532000
C	4.928938000	−0.736753000	1.333458000
C	4.912736000	0.666907000	1.361768000
H	4.720143000	1.189375000	2.312138000
C	5.195081000	−2.959920000	0.090391000
H	5.755101000	−3.275779000	−0.811113000
H	5.752310000	−3.353100000	0.963628000
C	3.811278000	−3.606569000	0.045509000
C	3.119946000	−3.700213000	−1.177907000
H	3.545861000	−3.255298000	−2.088328000
C	1.889670000	−4.376810000	−1.296740000

C	1.341916000	−5.025543000	−0.155630000
C	2.005828000	−4.881509000	1.077165000
H	1.592276000	−5.350225000	1.980998000
C	3.212400000	−4.167342000	1.203377000
C	0.073631000	−5.871761000	−0.236857000
H	0.059686000	−6.428145000	−1.194723000
H	0.116169000	−6.629570000	0.568877000
H	5.622649000	0.895720000	−2.926969000
H	4.288031000	−3.244519000	2.510976000
H	4.393056000	2.766976000	−2.186884000
H	1.891000000	5.117085000	3.188343000
H	4.367244000	−0.927144000	3.194608000
H	0.378421000	−4.720050000	−2.499947000
O	−1.396880000	−4.900368000	−2.475747000
O	−3.392567000	−3.869769000	2.629960000
O	−5.522784000	1.338203000	−2.102273000
O	−4.678614000	−1.422909000	2.667569000
O	−3.619414000	3.352186000	−2.299551000
O	−1.402940000	5.124585000	2.509750000
C	−1.931878000	−4.614979000	−1.216772000
C	−1.227637000	−5.091033000	−0.087603000
C	−1.755448000	−4.798215000	1.180494000
H	−1.229499000	−5.131937000	2.085426000
C	−2.957674000	−4.077616000	1.349189000
C	−3.688350000	−3.657718000	0.204684000
C	−3.141877000	−3.921064000	−1.069427000
H	−3.694139000	−3.601508000	−1.967495000
C	−5.062879000	−3.003905000	0.327270000
H	−5.544244000	−3.356729000	1.260652000
H	−5.691992000	−3.362381000	−0.510478000
C	−5.067827000	−1.480861000	0.303177000
C	−4.866571000	−0.723443000	1.479473000
C	−4.892990000	0.680413000	1.451964000
H	−4.740036000	1.246571000	2.384303000
C	−5.117163000	1.389994000	0.256334000
C	−5.296458000	0.634838000	−0.923359000
C	−5.276433000	−0.770544000	−0.894727000
H	−5.446767000	−1.332852000	−1.826045000
C	−5.151048000	2.913375000	0.241644000
H	−5.639144000	3.258559000	1.172976000
H	−5.791764000	3.254814000	−0.595292000
C	−3.780151000	3.578689000	0.152572000
C	−3.148600000	4.034072000	1.325455000
H	−3.633754000	3.891647000	2.302013000
C	−1.908985000	4.704717000	1.307117000
C	−1.287706000	4.966379000	0.055426000
C	−1.895972000	4.470612000	−1.113302000
H	−1.430642000	4.648740000	−2.093029000
C	−3.117164000	3.768502000	−1.089056000

C	−0.015948000	5.804087000	−0.046117000
H	−0.024499000	6.574984000	0.749850000
H	−0.035550000	6.340736000	−1.013567000
H	−4.465523000	−0.797591000	3.386516000
H	−4.394547000	2.754737000	−2.184340000
H	−3.933506000	−3.045167000	2.668870000
H	−1.866673000	−4.373878000	−3.150204000
H	−5.462969000	0.727469000	−2.862034000
H	−0.431986000	5.286350000	2.443978000
H	−2.793708000	1.193617000	−3.191878000
H	−2.621997000	0.479812000	−3.420433000
H	−0.831629000	1.980821000	0.007827000
H	−0.839681000	1.308385000	0.377702000
H	2.914171000	1.067925000	−3.223039000
H	2.812672000	0.339683000	−3.445121000
H	2.085205000	−1.148702000	−0.055886000
H	1.854017000	−0.421636000	0.027174000
H	2.406751000	−2.611260000	3.885102000
H	1.998408000	−2.152473000	4.347713000
H	−0.069762000	−2.396007000	−3.153352000
H	−0.583297000	−1.841672000	−3.293026000
		**PPA−H_2−7**	
O	1.309674000	4.636798000	2.763482000
O	3.686863000	4.098708000	−2.253944000
O	5.750061000	−1.434788000	2.302203000
O	4.585194000	1.510960000	−2.285083000
O	3.911976000	−3.531120000	2.592316000
O	1.175440000	−4.921913000	−2.073842000
C	1.941220000	4.484498000	1.529722000
C	1.322671000	5.077302000	0.404322000
C	1.944697000	4.908945000	−0.844022000
H	1.492388000	5.341081000	−1.746800000
C	3.155588000	4.199277000	−0.990186000
C	3.799566000	3.661567000	0.155024000
C	3.158081000	3.800091000	1.404770000
H	3.635978000	3.377736000	2.303321000
C	5.173074000	2.999059000	0.072105000
H	5.703149000	3.383819000	−0.821901000
H	5.766152000	3.318596000	0.951108000
C	5.159355000	1.476562000	0.040195000
C	4.849492000	0.766832000	−1.141757000
C	4.850266000	−0.637152000	−1.163910000
H	4.611932000	−1.166738000	−2.099386000
C	5.159873000	−1.393285000	−0.016726000
C	5.449512000	−0.684521000	1.169470000
C	5.452722000	0.720944000	1.191782000
H	5.704167000	1.247594000	2.126116000
C	5.165898000	−2.916738000	−0.054134000
H	5.570847000	−3.239897000	−1.032448000

H	5.862549000	−3.295228000	0.719683000
C	3.796672000	−3.564042000	0.130738000
C	3.038165000	−3.929912000	−0.997837000
H	3.422720000	−3.726236000	−2.007521000
C	1.803627000	−4.600226000	−0.894129000
C	1.306673000	−4.936976000	0.394290000
C	2.045000000	−4.533105000	1.522581000
H	1.679687000	−4.774924000	2.530604000
C	3.270243000	−3.842261000	1.421231000
C	0.026113000	−5.748392000	0.578446000
H	0.008208000	−6.578992000	−0.155826000
H	0.059428000	−6.214456000	1.581520000
H	4.214620000	0.938586000	−2.984279000
H	4.621561000	−2.863822000	2.445816000
H	4.171660000	3.240361000	−2.319148000
H	1.764886000	4.089616000	3.431009000
H	5.850944000	−0.841089000	3.070895000
H	0.270619000	−5.290448000	−1.926211000
O	−1.477111000	−5.641078000	−1.829623000
O	−3.441478000	−2.941011000	2.638185000
O	−4.568799000	1.345364000	−2.399496000
O	−5.633285000	−1.258240000	2.414962000
O	−3.433719000	3.918341000	−2.411775000
O	−1.471928000	4.552588000	2.775611000
C	−2.006771000	−4.936628000	−0.746701000
C	−1.275499000	−4.960836000	0.461792000
C	−1.800212000	−4.252429000	1.555553000
H	−1.255656000	−4.239935000	2.510005000
C	−3.029735000	−3.560516000	1.489524000
C	−3.775443000	−3.574948000	0.279206000
C	−3.230641000	−4.255727000	−0.830949000
H	−3.801259000	−4.286615000	−1.772463000
C	−5.143561000	−2.910217000	0.160456000
H	−5.782493000	−3.213289000	1.013912000
H	−5.636734000	−3.298786000	−0.751416000
C	−5.118877000	−1.387068000	0.082131000
C	−5.384453000	−0.592026000	1.216243000
C	−5.395264000	0.810261000	1.134568000
H	−5.617284000	1.405852000	2.033406000
C	−5.123397000	1.478542000	−0.072738000
C	−4.828889000	0.682975000	−1.205415000
C	−4.832078000	−0.719212000	−1.125378000
H	−4.608165000	−1.315790000	−2.023656000
C	−5.107336000	2.997269000	−0.145307000
H	−5.756743000	3.388630000	0.661679000
H	−5.548096000	3.330165000	−1.104811000
C	−3.720351000	3.617243000	0.008387000
C	−3.159287000	3.787054000	1.288016000
H	−3.687771000	3.416925000	2.178193000

C	−1.926864000	4.440242000	1.487086000
C	−1.248948000	4.994996000	0.367129000
C	−1.781064000	4.772661000	−0.917145000
H	−1.264002000	5.165949000	−1.803831000
C	−2.986226000	4.072429000	−1.119809000
C	0.011308000	5.841661000	0.534056000
H	−0.021523000	6.353549000	1.515693000
H	−0.000990000	6.634061000	−0.239271000
H	−5.697075000	−0.606235000	3.139504000
H	−3.930508000	3.070369000	−2.472105000
H	−4.294252000	−2.457177000	2.528083000
H	−2.014961000	−5.473844000	−2.628246000
H	−4.277903000	0.702652000	−3.074097000
H	−0.493087000	4.678234000	2.778851000
H	−2.543697000	0.196430000	2.817624000
H	−2.670858000	−0.557143000	2.894524000
H	0.139874000	2.084989000	1.179300000
H	−0.024191000	1.956159000	1.917707000
H	2.465320000	2.857097000	−4.017483000
H	2.214101000	2.362195000	−4.549234000
H	2.123348000	−1.136163000	0.794954000
H	1.913247000	−0.437358000	1.032010000
H	−0.096192000	−2.967726000	−2.691683000
H	−0.568114000	−2.363724000	−2.747731000
H	−2.529968000	−4.786156000	−4.640905000
H	−2.135373000	−4.286641000	−4.212619000
H	1.608193000	0.907816000	−1.199391000
H	1.839282000	1.538246000	−1.568254000
2. **Li^+@PPA, inside the hollow**			
**Li^+@PPA–H_2–1**			
O	1.701042000	5.630620000	2.217748000
O	3.505220000	3.196356000	−2.447643000
O	4.799054000	−1.197167000	2.403737000
O	5.684384000	1.509680000	−2.383276000
O	3.761863000	−3.907700000	2.505944000
O	1.138058000	−4.171899000	−2.405167000
C	2.228356000	5.050084000	1.097324000
C	1.421274000	5.081731000	−0.082779000
C	1.924096000	4.452075000	−1.240429000
H	1.321826000	4.448172000	−2.160005000
C	3.178552000	3.776774000	−1.276950000
C	4.008207000	3.800722000	−0.108052000
C	3.507782000	4.445246000	1.058110000
H	4.133779000	4.463987000	1.963183000
C	5.385953000	3.127549000	−0.076708000
H	5.980464000	3.436504000	−0.958543000
H	5.929506000	3.495393000	0.813566000
C	5.339884000	1.596822000	−0.026763000

C	5.494847000	0.820246000	−1.209963000
C	5.466964000	−0.596649000	−1.146116000
H	5.620228000	−1.181760000	−2.065381000
C	5.215964000	−1.292316000	0.063128000
C	5.024771000	−0.511305000	1.248181000
C	5.123034000	0.901675000	1.194591000
H	5.007297000	1.483546000	2.121324000
C	5.126663000	−2.809168000	0.094727000
H	5.648991000	−3.207632000	−0.795760000
H	5.668461000	−3.194493000	0.980592000
C	3.697247000	−3.364645000	0.106874000
C	2.965317000	−3.448447000	−1.094041000
H	3.361827000	−3.018659000	−2.024709000
C	1.747695000	−4.155813000	−1.183682000
C	1.264023000	−4.840346000	−0.031176000
C	1.959011000	−4.695109000	1.183332000
H	1.612358000	−5.208740000	2.090696000
C	3.150666000	−3.946905000	1.283983000
C	0.066388000	−5.791285000	−0.101002000
H	0.141691000	−6.417224000	−1.011325000
H	0.144445000	−6.480121000	0.762745000
H	5.811786000	0.914867000	−3.152944000
H	4.323247000	−3.109711000	2.570736000
H	4.378003000	2.732950000	−2.445443000
H	2.304774000	5.580190000	2.987363000
H	4.631853000	−0.606480000	3.166765000
H	0.344518000	−4.747333000	−2.422768000
O	−1.495831000	−5.329014000	−2.432497000
O	−3.478905000	−3.547273000	2.456074000
O	−4.840925000	1.341603000	−2.205146000
O	−5.470789000	−1.646648000	2.454717000
O	−4.014948000	4.134529000	−2.258316000
O	−0.635789000	3.944659000	2.172415000
C	−2.058803000	−4.898485000	−1.259806000
C	−1.306457000	−5.117557000	−0.065583000
C	−1.860050000	−4.657599000	1.155938000
H	−1.286066000	−4.767083000	2.087682000
C	−3.102574000	−3.962975000	1.230206000
C	−3.874413000	−3.799589000	0.032682000
C	−3.329665000	−4.277427000	−1.186078000
H	−3.915889000	−4.161241000	−2.110828000
C	−5.254637000	−3.128613000	0.040989000
H	−5.844010000	−3.498535000	0.902712000
H	−5.796457000	−3.445131000	−0.869824000
C	−5.224741000	−1.597595000	0.082820000
C	−5.321312000	−0.893238000	1.316197000
C	−5.285775000	0.525309000	1.335937000
H	−5.384385000	1.054918000	2.295774000
C	−5.099813000	1.292447000	0.158799000

C	−4.988618000	0.585490000	−1.081023000
C	−5.080710000	−0.828904000	−1.104389000
H	−5.020393000	−1.354063000	−2.069764000
C	−5.002616000	2.807897000	0.209982000
H	−5.400944000	3.150834000	1.183837000
H	−5.656489000	3.247364000	−0.569012000
C	−3.586858000	3.370016000	0.036762000
C	−2.676685000	3.345862000	1.113827000
H	−2.923885000	2.820493000	2.047074000
C	−1.462643000	4.056601000	1.076020000
C	−1.150526000	4.847212000	−0.065420000
C	−2.027445000	4.818476000	−1.163485000
H	−1.815787000	5.409216000	−2.065467000
C	−3.225064000	4.071090000	−1.146092000
C	0.063406000	5.777924000	−0.096317000
H	0.014264000	6.488597000	0.752285000
H	−0.005314000	6.386698000	−1.017467000
H	−5.549923000	−1.095192000	3.261950000
H	−4.558728000	3.324244000	−2.321052000
H	−4.299321000	−2.997265000	2.458753000
H	−2.071180000	−5.164346000	−3.207392000
H	−4.726456000	0.798804000	−3.012076000
H	−0.056399000	4.730002000	2.275707000
Li	−7.071216000	−0.040746000	−0.083498000
Li	3.459150000	6.085548000	−0.635160000
Li	7.168252000	0.061298000	0.329100000
Li	−3.028795000	−6.577941000	0.591696000
H	1.624435000	1.813195000	1.433335000
H	1.067391000	2.275476000	1.695354000
	**Li^{+}@PPA−H_2−2**		
O	1.676370000	5.581156000	2.239020000
O	3.511576000	3.249960000	−2.466233000
O	4.814720000	−1.213162000	2.357048000
O	5.664444000	1.539348000	−2.410516000
O	3.801420000	−3.928920000	2.450971000
O	1.088000000	−4.137492000	−2.414314000
C	2.212155000	5.028540000	1.108742000
C	1.413722000	5.087844000	−0.075968000
C	1.925271000	4.485084000	−1.244146000
H	1.329980000	4.501682000	−2.168195000
C	3.178581000	3.807544000	−1.286509000
C	4.001273000	3.807311000	−0.112515000
C	3.492026000	4.425399000	1.064428000
H	4.111132000	4.422977000	1.974383000
C	5.379517000	3.134925000	−0.085997000
H	5.974391000	3.453526000	−0.964157000
H	5.921621000	3.494554000	0.808491000
C	5.335052000	1.603940000	−0.050932000
C	5.481327000	0.839022000	−1.242823000

C	5.453147000	−0.578474000	−1.192366000
H	5.598850000	−1.154971000	−2.118315000
C	5.211788000	−1.285789000	0.012074000
C	5.030526000	−0.516380000	1.206258000
C	5.127792000	0.897062000	1.165286000
H	5.019778000	1.469819000	2.098649000
C	5.122585000	−2.802828000	0.030319000
H	5.631404000	−3.192859000	−0.871680000
H	5.677871000	−3.196617000	0.904043000
C	3.693615000	−3.358622000	0.060021000
C	2.940967000	−3.429485000	−1.129005000
H	3.321382000	−2.989689000	−2.061696000
C	1.722285000	−4.136344000	−1.204691000
C	1.258015000	−4.831750000	−0.050886000
C	1.974432000	−4.700809000	1.152527000
H	1.642432000	−5.223110000	2.060405000
C	3.168181000	−3.954310000	1.239983000
C	0.054757000	−5.775618000	−0.108286000
H	0.125731000	−6.416016000	−1.008945000
H	0.127102000	−6.452048000	0.765718000
H	5.786654000	0.952209000	−3.186860000
H	4.359530000	−3.128695000	2.516289000
H	4.378215000	2.774975000	−2.463357000
H	2.273248000	5.510117000	3.012275000
H	4.655144000	−0.630025000	3.127471000
H	0.317238000	−4.742863000	−2.435141000
O	−1.517108000	−5.366206000	−2.442964000
O	−3.456422000	−3.430485000	2.404091000
O	−4.832936000	1.353478000	−2.294254000
O	−5.498682000	−1.591387000	2.386550000
O	−3.962497000	4.133390000	−2.348582000
O	−0.712857000	3.965788000	2.179155000
C	−2.072529000	−4.903227000	−1.279624000
C	−1.313363000	−5.091643000	−0.084381000
C	−1.856095000	−4.592361000	1.126274000
H	−1.275711000	−4.676810000	2.056647000
C	−3.095963000	−3.893316000	1.187933000
C	−3.877963000	−3.764507000	−0.006489000
C	−3.342515000	−4.278991000	−1.214777000
H	−3.935065000	−4.188429000	−2.138374000
C	−5.259973000	−3.096375000	−0.009461000
H	−5.852531000	−3.459470000	0.852989000
H	−5.796879000	−3.424835000	−0.918916000
C	−5.237760000	−1.565067000	0.016385000
C	−5.351102000	−0.849006000	1.240618000
C	−5.323565000	0.569087000	1.247512000
H	−5.433930000	1.106696000	2.201265000
C	−5.125705000	1.324710000	0.065710000
C	−4.995449000	0.606742000	−1.165882000

C	−5.083413000	−0.807880000	−1.177117000
H	−5.007870000	−1.342314000	−2.136259000
C	−5.027976000	2.839831000	0.105418000
H	−5.450480000	3.192867000	1.065398000
H	−5.658738000	3.274324000	−0.694805000
C	−3.604844000	3.392165000	−0.033864000
C	−2.724806000	3.370651000	1.068004000
H	−3.003208000	2.856312000	1.998765000
C	−1.504766000	4.073278000	1.058295000
C	−1.159029000	4.855082000	−0.079689000
C	−2.004403000	4.819897000	−1.202409000
H	−1.765337000	5.403440000	−2.102191000
C	−3.205339000	4.077861000	−1.213447000
C	0.056158000	5.784828000	−0.083850000
H	0.001936000	6.479215000	0.777666000
H	−0.005725000	6.411484000	−0.993530000
H	−5.572078000	−1.030651000	3.187931000
H	−4.512412000	3.327547000	−2.416333000
H	−4.292133000	−2.902703000	2.401666000
H	−2.095104000	−5.218944000	−3.219487000
H	−4.708967000	0.804035000	−3.095260000
H	−0.102886000	4.728532000	2.273872000
Li	−7.088195000	−0.021922000	−0.179044000
Li	3.458124000	6.102281000	−0.590175000
Li	7.168454000	0.066677000	0.278705000
Li	−3.039479000	−6.526179000	0.611468000
H	1.602375000	1.872801000	1.578508000
H	1.011427000	2.301546000	1.822473000
H	−2.396798000	−0.241872000	2.249760000
H	−2.517055000	−0.950006000	2.517716000
	**Li^+@PPA−H_2−3**		
O	1.689828000	5.560576000	2.236599000
O	3.481005000	3.214804000	−2.478085000
O	4.846759000	−1.220375000	2.364294000
O	5.633642000	1.496475000	−2.432161000
O	3.767645000	−3.899843000	2.468572000
O	1.150850000	−4.198191000	−2.444189000
C	2.216247000	5.006503000	1.102991000
C	1.410898000	5.069728000	−0.076702000
C	1.910606000	4.461966000	−1.247395000
H	1.308488000	4.480316000	−2.166981000
C	3.159918000	3.777706000	−1.297677000
C	3.992653000	3.778957000	−0.130777000
C	3.494142000	4.399702000	1.049528000
H	4.120741000	4.397151000	1.954355000
C	5.372423000	3.109037000	−0.116106000
H	5.955757000	3.423538000	−1.003517000
H	5.924258000	3.476527000	0.769202000
C	5.335181000	1.578236000	−0.069288000

C	5.470406000	0.804314000	−1.256583000
C	5.453985000	−0.612875000	−1.194274000
H	5.593998000	−1.195611000	−2.117134000
C	5.230929000	−1.311191000	0.018195000
C	5.056657000	−0.533029000	1.206730000
C	5.148295000	0.879994000	1.154524000
H	5.046125000	1.459422000	2.084412000
C	5.143233000	−2.828971000	0.047594000
H	5.663138000	−3.225483000	−0.845138000
H	5.689207000	−3.215119000	0.930441000
C	3.713788000	−3.383940000	0.062391000
C	2.984547000	−3.477997000	−1.139086000
H	3.388402000	−3.065807000	−2.074512000
C	1.758958000	−4.172057000	−1.222077000
C	1.264695000	−4.833924000	−0.061057000
C	1.959052000	−4.680860000	1.152390000
H	1.606126000	−5.179503000	2.065667000
C	3.159455000	−3.945228000	1.245814000
C	0.057174000	−5.772428000	−0.119972000
H	0.127099000	−6.411700000	−1.021477000
H	0.127450000	−6.450026000	0.753408000
H	5.752865000	0.904189000	−3.205031000
H	4.347693000	−3.114584000	2.522968000
H	4.344885000	2.734735000	−2.480063000
H	2.290165000	5.484606000	3.006673000
H	4.700952000	−0.631263000	3.132885000
H	0.351203000	−4.765186000	−2.454962000
O	−1.501857000	−5.334569000	−2.457629000
O	−3.465365000	−3.451329000	2.400296000
O	−4.853697000	1.357125000	−2.296265000
O	−5.491665000	−1.596399000	2.382774000
O	−3.955163000	4.123138000	−2.347971000
O	−0.735684000	3.985129000	2.202279000
C	−2.062909000	−4.884409000	−1.291858000
C	−1.309778000	−5.086103000	−0.095214000
C	−1.858222000	−4.599036000	1.118101000
H	−1.282135000	−4.692172000	2.050332000
C	−3.098463000	−3.900764000	1.180929000
C	−3.875304000	−3.761035000	−0.015384000
C	−3.333747000	−4.262280000	−1.226296000
H	−3.922534000	−4.163719000	−2.151462000
C	−5.259003000	−3.096477000	−0.017201000
H	−5.850388000	−3.463250000	0.844497000
H	−5.794923000	−3.424797000	−0.927295000
C	−5.241099000	−1.565334000	0.011628000
C	−5.350398000	−0.851745000	1.237705000
C	−5.324433000	0.566352000	1.246943000
H	−5.427977000	1.101718000	2.202710000
C	−5.133055000	1.324821000	0.065821000

C	−5.008763000	0.608981000	−1.167656000
C	−5.094052000	−0.805741000	−1.181083000
H	−5.023178000	−1.338144000	−2.141710000
C	−5.034736000	2.840224000	0.108366000
H	−5.459080000	3.191662000	1.068062000
H	−5.663911000	3.276304000	−0.692293000
C	−3.610981000	3.392720000	−0.026789000
C	−2.738035000	3.380292000	1.080926000
H	−3.022778000	2.874938000	2.014591000
C	−1.516990000	4.081420000	1.073609000
C	−1.162143000	4.851360000	−0.069602000
C	−2.001397000	4.809264000	−1.196524000
H	−1.756119000	5.385791000	−2.099122000
C	−3.204492000	4.070723000	−1.208642000
C	0.057397000	5.774987000	−0.076126000
H	0.011120000	6.467245000	0.787461000
H	−0.005000000	6.404551000	−0.983811000
H	−5.564735000	−1.037424000	3.185388000
H	−4.514896000	3.323782000	−2.410996000
H	−4.298070000	−2.918799000	2.397906000
H	−2.077795000	−5.182224000	−3.234659000
H	−4.732140000	0.808846000	−3.098418000
H	−0.110461000	4.736856000	2.283578000
Li	−7.095637000	−0.022072000	−0.178699000
Li	3.455428000	6.074048000	−0.604476000
Li	7.181735000	0.051159000	0.251864000
Li	−3.023513000	−6.536787000	0.598279000
H	1.581826000	1.921550000	1.615663000
H	0.980839000	2.320561000	1.882920000
H	−2.392997000	−0.264635000	2.214466000
H	−2.514204000	−0.970831000	2.486405000
H	2.152819000	−0.345240000	−0.402032000
H	2.139547000	−0.346018000	0.363541000
	**Li^+@PPA−H_2−4**		
O	1.673026000	5.570345000	2.291423000
O	3.497769000	3.156966000	−2.375758000
O	4.808284000	−1.254079000	2.444289000
O	5.692090000	1.497845000	−2.315175000
O	3.845478000	−4.003121000	2.536025000
O	1.029561000	−4.059854000	−2.275224000
C	2.208348000	5.001118000	1.169801000
C	1.411202000	5.047216000	−0.016452000
C	1.920138000	4.422748000	−1.174221000
H	1.324871000	4.425699000	−2.098390000
C	3.171821000	3.743035000	−1.205090000
C	3.996624000	3.760167000	−0.033518000
C	3.488193000	4.396783000	1.134096000
H	4.107631000	4.407982000	2.043747000
C	5.378828000	3.095313000	0.002364000

H	5.976985000	3.416002000	−0.872828000
H	5.913103000	3.462058000	0.898656000
C	5.347225000	1.564380000	0.041499000
C	5.513529000	0.798830000	−1.146208000
C	5.491955000	−0.617679000	−1.095313000
H	5.645004000	−1.193741000	−2.020021000
C	5.235407000	−1.323718000	0.105409000
C	5.037269000	−0.555202000	1.297356000
C	5.129572000	0.858316000	1.256503000
H	5.006390000	1.431833000	2.187478000
C	5.135581000	−2.839025000	0.120856000
H	5.632976000	−3.232106000	−0.786240000
H	5.694558000	−3.241285000	0.988233000
C	3.701210000	−3.379030000	0.162441000
C	2.923279000	−3.411196000	−1.012809000
H	3.288169000	−2.949785000	−1.941256000
C	1.699972000	−4.108250000	−1.079895000
C	1.253617000	−4.835730000	0.059606000
C	1.994700000	−4.741270000	1.251326000
H	1.676736000	−5.284699000	2.151815000
C	3.192770000	−4.000646000	1.335821000
C	0.047463000	−5.776605000	0.001640000
H	0.120545000	−6.422126000	−0.895381000
H	0.119144000	−6.450236000	0.877774000
H	5.817955000	0.909192000	−3.089805000
H	4.400908000	−3.201705000	2.610974000
H	4.373501000	2.697669000	−2.373621000
H	2.268875000	5.509148000	3.066354000
H	4.633415000	−0.672140000	3.212262000
H	0.304881000	−4.719510000	−2.326382000
O	−1.520551000	−5.381444000	−2.337543000
O	−3.471451000	−3.421892000	2.494087000
O	−4.812548000	1.313650000	−2.234114000
O	−5.527027000	−1.596263000	2.461355000
O	−3.959259000	4.099788000	−2.302474000
O	−0.722815000	3.952593000	2.235353000
C	−2.082984000	−4.923729000	−1.175690000
C	−1.323039000	−5.097581000	0.020017000
C	−1.866640000	−4.590052000	1.226930000
H	−1.284492000	−4.662577000	2.157258000
C	−3.109725000	−3.896828000	1.282730000
C	−3.893185000	−3.784492000	0.087544000
C	−3.356947000	−4.308264000	−1.115578000
H	−3.948103000	−4.225298000	−2.040586000
C	−5.274920000	−3.117142000	0.077770000
H	−5.869821000	−3.473496000	0.941336000
H	−5.810099000	−3.450978000	−0.830713000
C	−5.248931000	−1.586010000	0.093224000
C	−5.372168000	−0.861495000	1.311508000

C	−5.343651000	0.556433000	1.307998000
H	−5.460529000	1.101072000	2.256936000
C	−5.132821000	1.303171000	0.122872000
C	−4.989062000	0.576005000	−1.101958000
C	−5.078851000	−0.838356000	−1.104055000
H	−4.993669000	−1.380041000	−2.058233000
C	−5.033331000	2.818538000	0.153076000
H	−5.460158000	3.178237000	1.108632000
H	−5.659539000	3.248825000	−0.653057000
C	−3.609074000	3.368766000	0.016696000
C	−2.733206000	3.353639000	1.122064000
H	−3.014366000	2.845348000	2.055245000
C	−1.512394000	4.054698000	1.111980000
C	−1.161346000	4.828137000	−0.029852000
C	−2.003289000	4.788452000	−1.154821000
H	−1.759994000	5.366254000	−2.057122000
C	−3.205358000	4.048433000	−1.165008000
C	0.057816000	5.751964000	−0.036208000
H	0.003845000	6.453619000	0.819345000
H	0.003340000	6.371307000	−0.951388000
H	−5.615526000	−1.030563000	3.257680000
H	−4.506696000	3.292250000	−2.369959000
H	−4.312872000	−2.903361000	2.488602000
H	−2.097714000	−5.235833000	−3.115020000
H	−4.672472000	0.757917000	−3.028118000
H	−0.116073000	4.717878000	2.329727000
Li	−7.092013000	−0.047846000	−0.138694000
Li	3.456496000	6.049843000	−0.545293000
Li	7.185626000	0.037049000	0.389110000
Li	−3.045084000	−6.530103000	0.738393000
H	1.554461000	1.831177000	1.602028000
H	0.967939000	2.271080000	1.836401000
H	−2.370998000	−0.248346000	2.253280000
H	−2.537575000	−0.950976000	2.509637000
H	2.572382000	0.659594000	−2.113694000
H	2.532213000	−0.064525000	−1.864325000
H	−0.683427000	−2.349645000	−2.138659000
H	−1.191433000	−1.816838000	−1.916105000
	**Li^+@PPA−H_2−5**		
O	1.683793000	5.574124000	2.287126000
O	3.502346000	3.174015000	−2.389190000
O	4.793035000	−1.233686000	2.438023000
O	5.686745000	1.501645000	−2.328894000
O	3.824073000	−3.972715000	2.539024000
O	1.033316000	−4.099815000	−2.285377000
C	2.218110000	5.009830000	1.162587000
C	1.418517000	5.057581000	−0.021851000
C	1.925918000	4.437305000	−1.182742000
H	1.328363000	4.441027000	−2.105428000

C	3.178107000	3.758971000	−1.217543000
C	4.005002000	3.774036000	−0.047328000
C	3.498455000	4.407170000	1.122714000
H	4.119453000	4.416270000	2.031312000
C	5.385180000	3.105655000	−0.014206000
H	5.982412000	3.422291000	−0.891427000
H	5.922791000	3.471763000	0.880353000
C	5.346080000	1.575107000	0.028020000
C	5.506317000	0.806132000	−1.158386000
C	5.478505000	−0.610361000	−1.103885000
H	5.629765000	−1.189471000	−2.026976000
C	5.220004000	−1.312096000	0.099009000
C	5.024972000	−0.539486000	1.288779000
C	5.124423000	0.873338000	1.244670000
H	5.004825000	1.449626000	2.174407000
C	5.119258000	−2.827596000	0.118786000
H	5.619294000	−3.222363000	−0.786128000
H	5.676754000	−3.226769000	0.988623000
C	3.686637000	−3.371903000	0.159046000
C	2.914790000	−3.421431000	−1.019663000
H	3.282767000	−2.969865000	−1.951745000
C	1.696464000	−4.127249000	−1.085069000
C	1.248389000	−4.844135000	0.060344000
C	1.982391000	−4.732071000	1.254615000
H	1.663478000	−5.267596000	2.159461000
C	3.176102000	−3.984217000	1.336467000
C	0.046178000	−5.790015000	0.006919000
H	0.121342000	−6.440518000	−0.886281000
H	0.118722000	−6.458131000	0.887155000
H	5.808221000	0.910743000	−3.102493000
H	4.375669000	−3.168206000	2.609497000
H	4.377835000	2.714512000	−2.388532000
H	2.280490000	5.509057000	3.061094000
H	4.621216000	−0.648735000	3.204393000
H	0.308550000	−4.759969000	−2.329996000
O	−1.520290000	−5.407282000	−2.335846000
O	−3.473594000	−3.424538000	2.485971000
O	−4.791335000	1.315793000	−2.233395000
O	−5.534769000	−1.605882000	2.450300000
O	−3.958249000	4.107210000	−2.295998000
O	−0.700150000	3.941260000	2.225751000
C	−2.081613000	−4.939740000	−1.177069000
C	−1.324470000	−5.111557000	0.020564000
C	−1.869051000	−4.598953000	1.224965000
H	−1.288821000	−4.670455000	2.156529000
C	−3.110099000	−3.902091000	1.276340000
C	−3.890170000	−3.790124000	0.078844000
C	−3.353081000	−4.318772000	−1.121705000
H	−3.942285000	−4.237008000	−2.048038000

C	−5.270846000	−3.120521000	0.064483000
H	−5.869855000	−3.478284000	0.924645000
H	−5.802595000	−3.451659000	−0.846986000
C	−5.243045000	−1.589576000	0.083914000
C	−5.372570000	−0.868311000	1.303339000
C	−5.340563000	0.549517000	1.303992000
H	−5.462080000	1.092081000	2.253539000
C	−5.121873000	1.299108000	0.122144000
C	−4.973892000	0.575186000	−1.104151000
C	−5.066273000	−0.838898000	−1.110504000
H	−4.976903000	−1.378451000	−2.065518000
C	−5.019859000	2.813966000	0.156993000
H	−5.443312000	3.171484000	1.114897000
H	−5.648414000	3.247769000	−0.645520000
C	−3.595873000	3.364039000	0.017203000
C	−2.714163000	3.342803000	1.117784000
H	−2.989099000	2.827583000	2.049020000
C	−1.495107000	4.047124000	1.106313000
C	−1.152308000	4.829872000	−0.031464000
C	−1.999043000	4.794908000	−1.153020000
H	−1.761810000	5.378962000	−2.052932000
C	−3.198901000	4.051599000	−1.162318000
C	0.063298000	5.758228000	−0.037110000
H	0.008223000	6.457131000	0.820681000
H	0.005144000	6.380370000	−0.950165000
H	−5.626647000	−1.042071000	3.247578000
H	−4.501047000	3.296813000	−2.366697000
H	−4.316349000	−2.908318000	2.478762000
H	−2.093818000	−5.258145000	−3.115333000
H	−4.647202000	0.761906000	−3.027954000
H	−0.100380000	4.711444000	2.324978000
Li	−7.081943000	−0.049135000	−0.153398000
Li	3.461233000	6.063729000	−0.552815000
Li	7.174689000	0.040302000	0.376979000
Li	−3.044290000	−6.542316000	0.733949000
H	1.676888000	1.857349000	1.839071000
H	1.070415000	2.298851000	2.010758000
H	−2.415186000	−0.226070000	2.249255000
H	−2.555113000	−0.918803000	2.545399000
H	2.593532000	0.676004000	−2.208724000
H	2.550173000	−0.053866000	−1.977879000
H	−0.665643000	−2.407474000	−2.265951000
H	−1.211567000	−1.883047000	−2.128746000
H	0.235122000	−0.532316000	−0.077379000
H	0.078333000	−0.318124000	0.641625000
**Li^+@PPA−H_2−6**			
O	1.714167000	5.527211000	2.295621000
O	3.515087000	3.112790000	−2.380024000
O	4.839343000	−1.303027000	2.427196000

O	5.710313000	1.454249000	−2.331101000
O	3.888353000	−4.054980000	2.514521000
O	1.096055000	−4.113130000	−2.309738000
C	2.244888000	4.959790000	1.170846000
C	1.443372000	5.009168000	−0.012233000
C	1.944985000	4.383059000	−1.172560000
H	1.345281000	4.387152000	−2.093848000
C	3.194708000	3.699893000	−1.208552000
C	4.024734000	3.715914000	−0.040547000
C	3.522814000	4.352159000	1.129807000
H	4.145637000	4.360190000	2.037143000
C	5.405594000	3.048454000	−0.011111000
H	6.000895000	3.369233000	−0.888193000
H	5.943704000	3.413343000	0.883690000
C	5.372502000	1.517614000	0.026598000
C	5.537835000	0.753425000	−1.162206000
C	5.524280000	−0.663145000	−1.111644000
H	5.680266000	−1.238239000	−2.036440000
C	5.274053000	−1.370364000	0.089620000
C	5.069104000	−0.603241000	1.281194000
C	5.156653000	0.810534000	1.241089000
H	5.032045000	1.382962000	2.172577000
C	5.186158000	−2.886325000	0.105514000
H	5.690138000	−3.276305000	−0.799293000
H	5.743261000	−3.284549000	0.975832000
C	3.754743000	−3.432584000	0.140377000
C	2.980705000	−3.461615000	−1.037059000
H	3.351231000	−3.002083000	−1.964223000
C	1.755841000	−4.155869000	−1.108426000
C	1.301934000	−4.878905000	0.030998000
C	2.039936000	−4.787507000	1.224162000
H	1.715959000	−5.327161000	2.124674000
C	3.240504000	−4.051703000	1.311922000
C	0.088977000	−5.810184000	−0.028186000
H	0.154405000	−6.452588000	−0.928028000
H	0.156929000	−6.487661000	0.845264000
H	5.834888000	0.866236000	−3.106460000
H	4.443529000	−3.253683000	2.592172000
H	4.389534000	2.651094000	−2.381393000
H	2.312716000	5.463461000	3.068299000
H	4.661694000	−0.721561000	3.194863000
H	0.352937000	−4.752544000	−2.354661000
O	−1.476055000	−5.385781000	−2.363334000
O	−3.410402000	−3.446008000	2.482439000
O	−4.777061000	1.327353000	−2.204839000
O	−5.469913000	−1.623004000	2.467121000
O	−3.962458000	4.120801000	−2.260869000
O	−0.667797000	3.896961000	2.231277000
C	−2.034700000	−4.931449000	−1.197793000

C	−1.275686000	−5.119568000	−0.003858000
C	−1.814049000	−4.615213000	1.206603000
H	−1.231132000	−4.696718000	2.135604000
C	−3.052817000	−3.914809000	1.268217000
C	−3.836829000	−3.790629000	0.074560000
C	−3.304435000	−4.308176000	−1.132923000
H	−3.896426000	−4.215702000	−2.056482000
C	−5.218478000	−3.122972000	0.072118000
H	−5.810663000	−3.486778000	0.934454000
H	−5.754832000	−3.451189000	−0.837749000
C	−5.197419000	−1.592028000	0.098864000
C	−5.324256000	−0.877857000	1.322749000
C	−5.307671000	0.539975000	1.329944000
H	−5.427064000	1.077526000	2.282638000
C	−5.104582000	1.296221000	0.149710000
C	−4.953078000	0.579945000	−1.079820000
C	−5.033318000	−0.834412000	−1.092428000
H	−4.941809000	−1.368167000	−2.050479000
C	−5.011231000	2.811648000	0.190432000
H	−5.430612000	3.163591000	1.152151000
H	−5.644957000	3.246379000	−0.607207000
C	−3.589221000	3.363790000	0.045756000
C	−2.698154000	3.328017000	1.139074000
H	−2.968678000	2.806040000	2.067682000
C	−1.472662000	4.020643000	1.121363000
C	−1.131641000	4.805686000	−0.015708000
C	−1.988271000	4.785272000	−1.129763000
H	−1.752168000	5.370529000	−2.029153000
C	−3.195885000	4.054150000	−1.133245000
C	0.093714000	5.721251000	−0.026693000
H	0.047134000	6.422569000	0.829660000
H	0.039519000	6.341365000	−0.941333000
H	−5.565325000	−1.064482000	3.267666000
H	−4.507675000	3.312088000	−2.333963000
H	−4.247432000	−2.920241000	2.480974000
H	−2.052848000	−5.231450000	−3.139385000
H	−4.614469000	0.778654000	−2.999333000
H	−0.063616000	4.663263000	2.335215000
Li	−7.051171000	−0.065807000	−0.122438000
Li	3.487534000	6.003466000	−0.551912000
Li	7.213294000	−0.002044000	0.371318000
Li	−3.001730000	−6.547762000	0.699902000
H	1.689313000	1.796993000	1.792828000
H	1.063415000	2.226826000	1.918311000
H	−2.277322000	−0.352754000	1.978374000
H	−2.396443000	−1.011419000	2.351452000
H	2.576214000	0.598572000	−2.062959000
H	2.503199000	−0.120216000	−1.806052000
H	−0.555852000	−2.379360000	−2.116261000

H	−1.065660000	−1.841949000	−1.910043000
H	−1.691624000	0.443039000	−0.934207000
H	−1.950617000	1.081463000	−0.595242000
H	1.057773000	−1.721653000	0.536999000
H	1.082073000	−1.483449000	1.265879000
	Li^+@PPA–H_2–7		
O	1.811550000	5.459692000	2.345592000
O	3.643550000	3.020716000	−2.304139000
O	4.884133000	−1.379511000	2.545758000
O	5.822843000	1.337703000	−2.222092000
O	3.911785000	−4.119428000	2.646638000
O	1.184798000	−4.210341000	−2.214426000
C	2.351392000	4.887500000	1.227517000
C	1.564107000	4.941444000	0.035224000
C	2.072598000	4.307961000	−1.117796000
H	1.481432000	4.310399000	−2.043982000
C	3.317096000	3.615456000	−1.138287000
C	4.134687000	3.627449000	0.038154000
C	3.625457000	4.270867000	1.201580000
H	4.238563000	4.276808000	2.115544000
C	5.510166000	2.950105000	0.083894000
H	6.115260000	3.260268000	−0.790229000
H	6.043472000	3.316942000	0.980784000
C	5.465721000	1.419800000	0.131740000
C	5.635877000	0.645876000	−1.050095000
C	5.612157000	−0.770200000	−0.989708000
H	5.774688000	−1.352714000	−1.908825000
C	5.344516000	−1.467191000	0.213982000
C	5.131851000	−0.689791000	1.397366000
C	5.231294000	0.722918000	1.348672000
H	5.100106000	1.302749000	2.274572000
C	5.249212000	−2.982934000	0.241140000
H	5.762312000	−3.382051000	−0.654476000
H	5.794914000	−3.375739000	1.121175000
C	3.815430000	−3.523556000	0.263487000
C	3.058191000	−3.562481000	−0.924162000
H	3.442307000	−3.113149000	−1.850709000
C	1.828549000	−4.246847000	−1.004356000
C	1.353172000	−4.952803000	0.137078000
C	2.075722000	−4.852371000	1.339377000
H	1.735300000	−5.379243000	2.241384000
C	3.281096000	−4.125473000	1.435073000
C	0.136111000	−5.878655000	0.071019000
H	0.213356000	−6.533091000	−0.819155000
H	0.187250000	−6.544571000	0.954415000
H	5.961182000	0.745233000	−2.991606000
H	4.473303000	−3.322359000	2.722222000
H	4.513182000	2.550355000	−2.293685000
H	2.402093000	5.396084000	3.124372000

H	4.703245000	−0.791492000	3.307625000
H	0.437823000	−4.845066000	−2.264266000
O	−1.386915000	−5.472896000	−2.294754000
O	−3.397084000	−3.477080000	2.497888000
O	−4.614207000	1.270823000	−2.241608000
O	−5.444823000	−1.642755000	2.430839000
O	−3.792188000	4.059813000	−2.302635000
O	−0.590987000	3.854265000	2.256992000
C	−1.962497000	−5.002266000	−1.144223000
C	−1.225264000	−5.181672000	0.064570000
C	−1.781958000	−4.662821000	1.260576000
H	−1.216500000	−4.738834000	2.200843000
C	−3.018456000	−3.956554000	1.293680000
C	−3.779726000	−3.838277000	0.084828000
C	−3.229141000	−4.370690000	−1.107816000
H	−3.803751000	−4.283566000	−2.042815000
C	−5.155267000	−3.159922000	0.050984000
H	−5.767886000	−3.512930000	0.903453000
H	−5.676434000	−3.488966000	−0.867338000
C	−5.119639000	−1.629162000	0.069059000
C	−5.265732000	−0.906105000	1.285787000
C	−5.235791000	0.511483000	1.284324000
H	−5.372059000	1.055854000	2.230813000
C	−4.999824000	1.258103000	0.104127000
C	−4.825946000	0.532415000	−1.116629000
C	−4.920473000	−0.880999000	−1.122970000
H	−4.812831000	−1.422242000	−2.075014000
C	−4.897811000	2.773120000	0.137126000
H	−5.334589000	3.133259000	1.088077000
H	−5.513897000	3.206208000	−0.675224000
C	−3.471020000	3.318948000	0.017588000
C	−2.602416000	3.287761000	1.128972000
H	−2.893301000	2.770953000	2.054280000
C	−1.374144000	3.976033000	1.131864000
C	−1.009800000	4.756155000	−0.001101000
C	−1.844807000	4.732848000	−1.131721000
H	−1.590924000	5.317067000	−2.026822000
C	−3.052627000	4.002370000	−1.156779000
C	0.220601000	5.664120000	0.004590000
H	0.167967000	6.365191000	0.860677000
H	0.182109000	6.285319000	−0.910132000
H	−5.548097000	−1.078680000	3.226513000
H	−4.337782000	3.251845000	−2.381364000
H	−4.231972000	−2.948325000	2.476647000
H	−1.949085000	−5.323366000	−3.082401000
H	−4.433707000	0.716172000	−3.028069000
H	0.021177000	4.613802000	2.363506000
Li	−6.951380000	−0.091350000	−0.202068000
Li	3.621193000	5.919297000	−0.483919000

Li	7.291068000	−0.108326000	0.505857000
Li	−2.967673000	−6.598382000	0.765916000
H	1.686712000	1.710287000	1.613822000
H	1.110439000	2.156058000	1.861758000
H	−2.254330000	−0.356191000	2.136703000
H	−2.389840000	−1.037721000	2.459357000
H	−0.117321000	2.347128000	−3.263164000
H	−0.603040000	2.731870000	−2.811070000
H	−0.455877000	−2.450931000	−2.084796000
H	−1.023907000	−1.969959000	−1.890849000
H	−1.630394000	0.282761000	−0.762772000
H	−1.843669000	0.987339000	−0.545204000
H	1.250224000	−1.738606000	0.562584000
H	1.249372000	−1.304906000	1.195408000
H	2.631453000	0.654732000	−1.697545000
H	2.491088000	0.044552000	−1.255391000
3. Li^+@PPA, H_2 is on Li^+			
Li^+@PPA–H_2–1			
O	1.712973000	5.385097000	2.469658000
O	3.639010000	3.364026000	−2.349156000
O	4.887475000	−1.389404000	2.430158000
O	5.641759000	1.461967000	−2.296042000
O	3.824880000	−4.084965000	2.484784000
O	1.023391000	−4.194491000	−2.333885000
C	2.257636000	4.886930000	1.314254000
C	1.498068000	5.060242000	0.118578000
C	2.031778000	4.532187000	−1.084094000
H	1.448759000	4.603185000	−2.014202000
C	3.267885000	3.826915000	−1.136210000
C	4.042092000	3.699939000	0.062331000
C	3.514819000	4.238630000	1.261320000
H	4.105537000	4.153938000	2.186629000
C	5.411743000	3.009627000	0.074076000
H	6.008538000	3.348535000	−0.795259000
H	5.956333000	3.341297000	0.977781000
C	5.360131000	1.478863000	0.071783000
C	5.470975000	0.740070000	−1.140173000
C	5.430502000	−0.678243000	−1.120918000
H	5.549006000	−1.234850000	−2.062899000
C	5.214138000	−1.411690000	0.072691000
C	5.074337000	−0.668569000	1.288340000
C	5.181276000	0.744797000	1.275903000
H	5.103396000	1.297287000	2.224539000
C	5.112017000	−2.928280000	0.058854000
H	5.599249000	−3.301872000	−0.861791000
H	5.682167000	−3.345838000	0.911665000
C	3.680041000	−3.475405000	0.105508000
C	2.906065000	−3.523144000	−1.071062000

H	3.274286000	−3.072632000	−2.003510000
C	1.680518000	−4.218765000	−1.135920000
C	1.229206000	−4.926055000	0.015673000
C	1.968316000	−4.819912000	1.207708000
H	1.646645000	−5.351692000	2.113808000
C	3.170679000	−4.085785000	1.284962000
C	0.014033000	−5.855258000	−0.032246000
H	0.074231000	−6.503410000	−0.928355000
H	0.079787000	−6.526167000	0.846389000
H	5.727603000	0.888022000	−3.086651000
H	4.396684000	−3.294352000	2.550323000
H	4.457954000	2.812695000	−2.333715000
H	2.294341000	5.248969000	3.245601000
H	4.762211000	−0.821725000	3.218159000
H	0.268234000	−4.818919000	−2.361813000
O	−1.562921000	−5.454385000	−2.364957000
O	−3.464758000	−3.449616000	2.469556000
O	−4.759395000	1.303111000	−2.288983000
O	−5.474015000	−1.561204000	2.435444000
O	−3.700202000	3.998258000	−2.347532000
O	−0.850190000	4.095131000	2.443020000
C	−2.106640000	−4.969340000	−1.204562000
C	−1.345183000	−5.153702000	−0.010140000
C	−1.877619000	−4.637148000	1.197871000
H	−1.295109000	−4.721360000	2.126975000
C	−3.108635000	−3.921036000	1.258045000
C	−3.891404000	−3.793638000	0.063077000
C	−3.367124000	−4.325829000	−1.142258000
H	−3.961712000	−4.236291000	−2.064701000
C	−5.261150000	−3.102292000	0.060170000
H	−5.853163000	−3.442761000	0.932171000
H	−5.810641000	−3.432122000	−0.841299000
C	−5.210190000	−1.571351000	0.065733000
C	−5.312597000	−0.835833000	1.280359000
C	−5.272546000	0.582580000	1.264671000
H	−5.383320000	1.136611000	2.209116000
C	−5.065832000	1.319299000	0.071259000
C	−4.936047000	0.579327000	−1.147567000
C	−5.041979000	−0.834102000	−1.138010000
H	−4.972294000	−1.383708000	−2.088973000
C	−4.963174000	2.835763000	0.088220000
H	−5.441150000	3.207083000	1.014607000
H	−5.541519000	3.255785000	−0.757883000
C	−3.531300000	3.381952000	0.028597000
C	−2.745553000	3.426659000	1.197634000
H	−3.104874000	2.974486000	2.132739000
C	−1.519348000	4.122048000	1.252058000
C	−1.078837000	4.830977000	0.097041000
C	−1.829819000	4.727657000	−1.087555000

H	−1.516038000	5.260187000	−1.995999000
C	−3.033691000	3.994977000	−1.154350000
C	0.136951000	5.759613000	0.134169000
H	0.077032000	6.417107000	1.023612000
H	0.067089000	6.421655000	−0.751239000
H	−5.557325000	−0.990307000	3.228620000
H	−4.272889000	3.208095000	−2.409573000
H	−4.288101000	−2.903918000	2.463347000
H	−2.142387000	−5.307217000	−3.140356000
H	−4.640111000	0.737793000	−3.079598000
H	−0.100503000	4.726768000	2.469447000
Li	−7.037559000	−0.011697000	−0.163479000
Li	3.151067000	6.523083000	−0.685697000
Li	7.181545000	−0.075802000	0.320602000
Li	−3.093454000	−6.549949000	0.684802000
H	4.040430000	6.995755000	−2.406261000
H	4.100610000	7.677624000	−2.054606000
	**Li^+@PPA–H_2–2**		
O	1.720781000	5.275975000	2.630797000
O	3.728307000	3.422266000	−2.221339000
O	4.882264000	−1.528710000	2.470477000
O	5.651517000	1.434830000	−2.184177000
O	3.799523000	−4.215397000	2.456968000
O	1.000744000	−4.197945000	−2.364657000
C	2.270244000	4.786441000	1.470762000
C	1.530111000	5.001727000	0.271786000
C	2.081924000	4.511939000	−0.937963000
H	1.517102000	4.617391000	−1.876215000
C	3.318260000	3.809555000	−0.991456000
C	4.062061000	3.621421000	0.215563000
C	3.514759000	4.119077000	1.422668000
H	4.085981000	3.996643000	2.355834000
C	5.428057000	2.924508000	0.226316000
H	6.035778000	3.283990000	−0.627111000
H	5.963573000	3.229993000	1.144594000
C	5.369944000	1.394913000	0.184064000
C	5.474834000	0.686453000	−1.046302000
C	5.424666000	−0.731602000	−1.062813000
H	5.538129000	−1.265354000	−2.018594000
C	5.205050000	−1.494007000	0.112070000
C	5.071965000	−0.781187000	1.346271000
C	5.188096000	0.631349000	1.369013000
H	5.114995000	1.160360000	2.331364000
C	5.094303000	−3.009196000	0.059977000
H	5.580444000	−3.362165000	−0.869396000
H	5.661841000	−3.450921000	0.902296000
C	3.659808000	−3.550349000	0.092454000
C	2.886570000	−3.567155000	−1.085526000
H	3.257783000	−3.097681000	−2.007431000

C	1.657288000	−4.254292000	−1.167230000
C	1.201097000	−4.985336000	−0.032643000
C	1.939920000	−4.910815000	1.162037000
H	1.614495000	−5.461209000	2.055601000
C	3.146135000	−4.185168000	1.257023000
C	−0.019973000	−5.905457000	−0.102368000
H	0.039305000	−6.537130000	−1.010175000
H	0.037878000	−6.593344000	0.763654000
H	5.730740000	0.879231000	−2.988345000
H	4.374308000	−3.428643000	2.541355000
H	4.521292000	2.834382000	−2.208312000
H	2.285439000	5.099903000	3.410987000
H	4.762727000	−0.979288000	3.272163000
H	0.247328000	−4.823320000	−2.410805000
O	−1.587500000	−5.454000000	−2.432459000
O	−3.488322000	−3.517533000	2.430236000
O	−4.745393000	1.319707000	−2.257112000
O	−5.483294000	−1.612935000	2.421689000
O	−3.639609000	3.995167000	−2.263575000
O	−0.860482000	4.032152000	2.569238000
C	−2.131103000	−4.985029000	−1.265468000
C	−1.374207000	−5.194865000	−0.072219000
C	−1.906260000	−4.694936000	1.142812000
H	−1.326943000	−4.799316000	2.071872000
C	−3.132335000	−3.971253000	1.211894000
C	−3.911144000	−3.818542000	0.017278000
C	−3.387350000	−4.333991000	−1.195575000
H	−3.978793000	−4.224750000	−2.117879000
C	−5.276441000	−3.118314000	0.022851000
H	−5.872091000	−3.468632000	0.888475000
H	−5.826590000	−3.430549000	−0.884520000
C	−5.215974000	−1.587984000	0.052410000
C	−5.315480000	−0.870853000	1.278173000
C	−5.266784000	0.547340000	1.284680000
H	−5.375116000	1.087377000	2.237497000
C	−5.054489000	1.301542000	0.103134000
C	−4.927284000	0.579767000	−1.126895000
C	−5.041765000	−0.832990000	−1.139309000
H	−4.974271000	−1.368203000	−2.098639000
C	−4.944189000	2.817123000	0.143912000
H	−5.431086000	3.177038000	1.070190000
H	−5.510653000	3.252408000	−0.702465000
C	−3.508910000	3.356289000	0.109212000
C	−2.739630000	3.386041000	1.289631000
H	−3.115078000	2.928766000	2.215946000
C	−1.509544000	4.072384000	1.368020000
C	−1.047724000	4.788200000	0.225564000
C	−1.782598000	4.700061000	−0.970364000
H	−1.452490000	5.238359000	−1.869567000

C	−2.990316000	3.976378000	−1.061011000
C	0.173380000	5.708603000	0.287867000
H	0.108001000	6.350680000	1.188103000
H	0.114404000	6.386513000	−0.586368000
H	−5.562895000	−1.053654000	3.223453000
H	−4.220561000	3.212240000	−2.339535000
H	−4.307093000	−2.965019000	2.430702000
H	−2.163315000	−5.288156000	−3.206796000
H	−4.628039000	0.765575000	−3.055897000
H	−0.093571000	4.642594000	2.605945000
Li	−7.033719000	−0.010719000	−0.151996000
Li	3.150990000	6.539924000	−0.545217000
Li	7.180024000	−0.178465000	0.389792000
Li	−3.137835000	−6.586928000	0.591227000
H	4.097290000	6.445843000	−2.327116000
H	4.304910000	7.160703000	−2.129477000
H	2.792772000	7.839376000	0.948476000
H	3.232232000	8.293902000	0.510091000
	Li^+@PPA–H_2–3		
O	1.512382000	4.893068000	2.793917000
O	3.861436000	3.910763000	−2.156650000
O	5.091674000	−1.530712000	2.409435000
O	5.380029000	1.580532000	−2.205080000
O	3.923715000	−4.177828000	2.395452000
O	0.859894000	−4.162802000	−2.262399000
C	2.130285000	4.603976000	1.601638000
C	1.453717000	5.006143000	0.417424000
C	2.091713000	4.735351000	−0.817665000
H	1.560044000	4.941073000	−1.761910000
C	3.342098000	4.060390000	−0.903333000
C	4.008738000	3.668315000	0.292432000
C	3.376138000	3.948512000	1.525576000
H	3.886231000	3.671621000	2.460877000
C	5.385875000	2.995017000	0.270002000
H	5.976536000	3.393266000	−0.577554000
H	5.926060000	3.276834000	1.193232000
C	5.343434000	1.468636000	0.180531000
C	5.322368000	0.800288000	−1.075979000
C	5.275952000	−0.616600000	−1.132897000
H	5.294494000	−1.118783000	−2.112149000
C	5.177294000	−1.418861000	0.032430000
C	5.168405000	−0.747714000	1.295560000
C	5.289083000	0.664459000	1.350880000
H	5.321275000	1.160681000	2.332802000
C	5.069721000	−2.933315000	−0.059502000
H	5.495320000	−3.253235000	−1.029641000
H	5.696162000	−3.395494000	0.728343000
C	3.646122000	−3.493431000	0.048866000
C	2.809572000	−3.513696000	−1.085548000

H	3.123652000	−3.032169000	−2.022155000
C	1.589992000	−4.220473000	−1.105690000
C	1.205496000	−4.964939000	0.046440000
C	2.007184000	−4.889327000	1.199027000
H	1.737402000	−5.448368000	2.105750000
C	3.207297000	−4.146864000	1.233320000
C	−0.006597000	−5.898845000	0.035329000
H	0.052403000	−6.579128000	−0.837230000
H	0.063556000	−6.537994000	0.936758000
H	5.368231000	1.044227000	−3.025542000
H	4.512729000	−3.398499000	2.447034000
H	4.517252000	3.173977000	−2.202302000
H	2.055311000	4.632497000	3.564938000
H	5.065337000	−1.006942000	3.236492000
H	0.187344000	−4.874171000	−2.315204000
O	−1.608100000	−5.625167000	−2.299579000
O	−3.477337000	−3.392056000	2.447474000
O	−4.725871000	1.197849000	−2.374067000
O	−5.563707000	−1.585184000	2.378107000
O	−3.442370000	3.795897000	−2.364966000
O	−1.205547000	4.130071000	2.728462000
C	−2.142517000	−5.083705000	−1.160014000
C	−1.367867000	−5.201504000	0.034432000
C	−1.892446000	−4.628708000	1.220003000
H	−1.301254000	−4.665667000	2.146691000
C	−3.128632000	−3.920094000	1.257175000
C	−3.921681000	−3.853901000	0.063841000
C	−3.405109000	−4.442015000	−1.118878000
H	−4.007384000	−4.399120000	−2.039744000
C	−5.292614000	−3.166210000	0.037598000
H	−5.892100000	−3.491524000	0.910257000
H	−5.833792000	−3.513508000	−0.862314000
C	−5.243588000	−1.635357000	0.016448000
C	−5.381067000	−0.878374000	1.214652000
C	−5.350522000	0.539444000	1.174047000
H	−5.490715000	1.109898000	2.104476000
C	−5.112062000	1.255025000	−0.026321000
C	−4.939591000	0.493723000	−1.226385000
C	−5.042279000	−0.919668000	−1.195222000
H	−4.941611000	−1.486410000	−2.133173000
C	−5.011803000	2.772193000	−0.031857000
H	−5.577434000	3.162077000	0.835640000
H	−5.503173000	3.173432000	−0.939272000
C	−3.577651000	3.309760000	0.046256000
C	−2.935303000	3.406982000	1.296019000
H	−3.410783000	3.011828000	2.204992000
C	−1.708481000	4.084433000	1.463671000
C	−1.120662000	4.728286000	0.335204000
C	−1.726305000	4.566072000	−0.924377000

H	−1.301942000	5.049347000	−1.815640000
C	−2.927820000	3.847566000	−1.099296000
C	0.080815000	5.670171000	0.475700000
H	−0.007873000	6.240046000	1.420835000
H	0.008971000	6.409717000	−0.346263000
H	−5.671616000	−1.001401000	3.158876000
H	−4.063236000	3.044760000	−2.445827000
H	−4.322593000	−2.881234000	2.431747000
H	−2.195053000	−5.516982000	−3.075836000
H	−4.580550000	0.618744000	−3.150227000
H	−0.317716000	4.547674000	2.762233000
Li	−7.066211000	−0.092271000	−0.292470000
Li	3.094737000	6.826454000	−1.001865000
Li	7.167676000	−0.141031000	0.156638000
Li	−3.110381000	−6.563974000	0.772878000
H	4.279050000	6.269539000	−2.563175000
H	4.357375000	7.035356000	−2.571957000
H	4.119434000	6.750864000	0.722711000
H	4.392566000	7.417405000	0.453968000
H	2.360417000	8.713477000	−1.376820000
H	1.730366000	8.287956000	−1.260380000
	**Li^+@PPA−H_2−4**		
O	1.583048000	4.975019000	2.800774000
O	3.936222000	3.775286000	−2.098438000
O	5.012533000	−1.572095000	2.539511000
O	5.520477000	1.488355000	−2.089170000
O	3.820092000	−4.209725000	2.500175000
O	0.966509000	−4.203805000	−2.289537000
C	2.201501000	4.627487000	1.623445000
C	1.532756000	4.986381000	0.422219000
C	2.169356000	4.658608000	−0.798263000
H	1.639745000	4.826266000	−1.750015000
C	3.414711000	3.974654000	−0.852765000
C	4.070746000	3.617683000	0.359003000
C	3.437647000	3.951918000	1.578144000
H	3.940349000	3.705123000	2.525809000
C	5.439876000	2.928214000	0.368640000
H	6.051432000	3.310361000	−0.471549000
H	5.965970000	3.213376000	1.298914000
C	5.383188000	1.401315000	0.293446000
C	5.409218000	0.719766000	−0.955906000
C	5.353948000	−0.697174000	−1.000821000
H	5.410166000	−1.209713000	−1.973239000
C	5.200772000	−1.486276000	0.167412000
C	5.142622000	−0.801293000	1.421946000
C	5.270788000	0.610341000	1.468568000
H	5.262019000	1.117076000	2.445584000
C	5.085721000	−3.000581000	0.086446000
H	5.551442000	−3.334140000	−0.860407000

H	5.671711000	−3.459257000	0.906645000
C	3.653411000	−3.545993000	0.136865000
C	2.867259000	−3.567475000	−1.032653000
H	3.227753000	−3.099425000	−1.959421000
C	1.639800000	−4.258464000	−1.099994000
C	1.197264000	−4.988134000	0.040576000
C	1.948878000	−4.910165000	1.226825000
H	1.633792000	−5.459253000	2.124890000
C	3.154836000	−4.181506000	1.307509000
C	−0.023055000	−5.909922000	−0.014543000
H	0.045138000	−6.569038000	−0.902192000
H	0.024974000	−6.571315000	0.872327000
H	5.542633000	0.943832000	−2.904004000
H	4.411893000	−3.434083000	2.572291000
H	4.606634000	3.050793000	−2.114799000
H	2.122255000	4.743247000	3.583541000
H	4.949271000	−1.039156000	3.358640000
H	0.247782000	−4.868043000	−2.344862000
O	−1.572977000	−5.546950000	−2.371647000
O	−3.514295000	−3.436879000	2.402336000
O	−4.680202000	1.264137000	−2.391006000
O	−5.544826000	−1.568952000	2.327073000
O	−3.416980000	3.871624000	−2.366120000
O	−1.095706000	4.095837000	2.696008000
C	−2.125539000	−5.035099000	−1.227073000
C	−1.377211000	−5.198999000	−0.021185000
C	−1.919338000	−4.655991000	1.170504000
H	−1.347201000	−4.726123000	2.107207000
C	−3.147478000	−3.933348000	1.204024000
C	−3.916449000	−3.824175000	−0.001807000
C	−3.382391000	−4.382207000	−1.191249000
H	−3.966085000	−4.306213000	−2.121865000
C	−5.281151000	−3.124154000	−0.032882000
H	−5.891146000	−3.455462000	0.830149000
H	−5.816587000	−3.455596000	−0.942197000
C	−5.219899000	−1.593535000	−0.034749000
C	−5.351915000	−0.850319000	1.172593000
C	−5.308799000	0.567642000	1.149344000
H	−5.444697000	1.127981000	2.086596000
C	−5.064530000	1.296145000	−0.042169000
C	−4.898697000	0.548228000	−1.251547000
C	−5.012351000	−0.864569000	−1.237434000
H	−4.916357000	−1.420522000	−2.182322000
C	−4.952765000	2.812312000	−0.029428000
H	−5.505434000	3.194961000	0.849600000
H	−5.451768000	3.228634000	−0.925899000
C	−3.514285000	3.339547000	0.036911000
C	−2.851797000	3.411175000	1.277920000
H	−3.314647000	3.001784000	2.187169000

C	−1.619590000	4.080718000	1.438915000
C	−1.045913000	4.741332000	0.313035000
C	−1.673014000	4.605767000	−0.939173000
H	−1.258675000	5.101757000	−1.828147000
C	−2.880653000	3.896506000	−1.108345000
C	0.167140000	5.668430000	0.446691000
H	0.074948000	6.262435000	1.376883000
H	0.114501000	6.387284000	−0.394486000
H	−5.645860000	−0.993310000	3.114754000
H	−4.031688000	3.116174000	−2.453701000
H	−4.342743000	−2.899406000	2.380309000
H	−2.143485000	−5.411142000	−3.155704000
H	−4.540739000	0.693004000	−3.174079000
H	−0.228442000	4.554695000	2.738805000
Li	−7.029620000	−0.032971000	−0.323493000
Li	3.101665000	6.816543000	−1.142548000
Li	7.191231000	−0.208111000	0.358755000
Li	−3.149508000	−6.563078000	0.658461000
H	2.107236000	7.151962000	−3.062516000
H	1.689442000	7.625639000	−2.626777000
H	4.365116000	6.490849000	0.478705000
H	4.611687000	7.195366000	0.302170000
H	2.384048000	8.701132000	−0.390357000
H	1.982135000	8.189174000	0.017069000
H	4.526755000	6.071333000	−2.500526000
H	4.678908000	6.825510000	−2.521784000
	**Li^+@PPA–H_2–5**		
O	1.517255000	4.868456000	2.767378000
O	3.972632000	3.916118000	−2.135694000
O	5.086257000	−1.549227000	2.496652000
O	5.393149000	1.508943000	−2.151736000
O	3.863032000	−4.169613000	2.500620000
O	0.893511000	−4.243410000	−2.217474000
C	2.156316000	4.573908000	1.584358000
C	1.509452000	4.989370000	0.388870000
C	2.169620000	4.728547000	−0.832222000
H	1.674322000	4.967893000	−1.785744000
C	3.417654000	4.054474000	−0.892793000
C	4.051065000	3.635319000	0.310328000
C	3.394409000	3.905301000	1.533340000
H	3.878423000	3.611125000	2.477166000
C	5.421737000	2.949727000	0.308548000
H	6.023803000	3.332602000	−0.537997000
H	5.956756000	3.234150000	1.234189000
C	5.364197000	1.423526000	0.235084000
C	5.331970000	0.742156000	−1.014503000
C	5.275049000	−0.674838000	−1.055836000
H	5.288685000	−1.187708000	−2.029599000
C	5.174042000	−1.463824000	0.118502000

C	5.169982000	−0.779063000	1.373882000
C	5.303710000	0.632571000	1.413776000
H	5.341246000	1.139305000	2.390085000
C	5.061521000	−2.979151000	0.042555000
H	5.500643000	−3.311497000	−0.917305000
H	5.674334000	−3.433934000	0.845173000
C	3.634989000	−3.534115000	0.134809000
C	2.821306000	−3.576261000	−1.015434000
H	3.156114000	−3.115950000	−1.955499000
C	1.599537000	−4.278769000	−1.044974000
C	1.189593000	−4.997166000	0.114726000
C	1.968417000	−4.899755000	1.281323000
H	1.678804000	−5.439392000	2.193605000
C	3.170453000	−4.160759000	1.323902000
C	−0.026220000	−5.926051000	0.099151000
H	0.041321000	−6.618477000	−0.763053000
H	0.026932000	−6.552757000	1.010331000
H	5.366159000	0.963493000	−2.965569000
H	4.461285000	−3.396751000	2.544959000
H	4.592648000	3.150251000	−2.176558000
H	2.042829000	4.593663000	3.545190000
H	5.064056000	−1.015880000	3.317653000
H	0.213533000	−4.947886000	−2.264985000
O	−1.592724000	−5.676093000	−2.261943000
O	−3.513049000	−3.362546000	2.425701000
O	−4.645356000	1.181807000	−2.462171000
O	−5.586231000	−1.542878000	2.304573000
O	−3.359411000	3.784999000	−2.458389000
O	−1.190729000	4.125982000	2.663971000
C	−2.138869000	−5.113251000	−1.138089000
C	−1.382479000	−5.220218000	0.069088000
C	−1.920369000	−4.627955000	1.238771000
H	−1.344171000	−4.658097000	2.175021000
C	−3.150745000	−3.908479000	1.247845000
C	−3.925148000	−3.850946000	0.041585000
C	−3.396053000	−4.459424000	−1.125412000
H	−3.983825000	−4.424042000	−2.055935000
C	−5.289157000	−3.151572000	−0.014013000
H	−5.905069000	−3.462138000	0.852505000
H	−5.819044000	−3.503895000	−0.918666000
C	−5.226265000	−1.621455000	−0.050692000
C	−5.378712000	−0.850335000	1.136545000
C	−5.339347000	0.566666000	1.080771000
H	−5.492942000	1.148222000	2.002123000
C	−5.075992000	1.267499000	−0.123087000
C	−4.885517000	0.491727000	−1.311172000
C	−4.998250000	−0.920434000	−1.266537000
H	−4.884494000	−1.498075000	−2.196250000
C	−4.967029000	2.783470000	−0.144043000

H	−5.544827000	3.186549000	0.709366000
H	−5.439563000	3.177438000	−1.064478000
C	−3.530392000	3.310615000	−0.047397000
C	−2.904342000	3.407481000	1.210653000
H	−3.394193000	3.018546000	2.114780000
C	−1.675644000	4.077631000	1.392531000
C	−1.068074000	4.714829000	0.270452000
C	−1.657610000	4.551233000	−0.996472000
H	−1.215988000	5.026617000	−1.883539000
C	−2.860876000	3.839350000	−1.185505000
C	0.134811000	5.652818000	0.423764000
H	0.029962000	6.224762000	1.366379000
H	0.077179000	6.388273000	−0.401770000
H	−5.699949000	−0.949525000	3.077250000
H	−3.974561000	3.029920000	−2.545409000
H	−4.353770000	−2.845100000	2.390909000
H	−2.166085000	−5.571171000	−3.048678000
H	−4.488563000	0.592830000	−3.228561000
H	−0.295579000	4.528496000	2.706857000
Li	−7.029923000	−0.072287000	−0.405910000
Li	3.312343000	7.191983000	−1.056207000
Li	7.165714000	−0.200577000	0.223343000
Li	−3.156644000	−6.550236000	0.771660000
H	2.000002000	7.653481000	−2.701678000
H	1.671967000	8.056603000	−2.137347000
H	4.578278000	6.328572000	0.374393000
H	4.902069000	7.023039000	0.321816000
H	2.357652000	8.462471000	0.416055000
H	2.088337000	7.762865000	0.580714000
H	4.391367000	6.128971000	−2.570980000
H	4.527158000	6.873389000	−2.712324000
H	4.090346000	9.170464000	−1.641455000
H	4.464442000	9.058236000	−0.980033000

4. **Li^+@PPA. Seven H_2 molecules inside the hollow and five H_2 molecules are on Li^+**

O	1.706891000	5.441561000	2.269031000
O	3.617487000	2.943318000	−2.317874000
O	4.988080000	−1.322610000	2.599895000
O	5.866014000	1.352649000	−2.203000000
O	4.071545000	−4.088424000	2.714836000
O	1.423388000	−4.272081000	−2.187161000
C	2.263376000	4.854255000	1.166598000
C	1.476855000	4.856766000	−0.027516000
C	2.005213000	4.208981000	−1.163616000
H	1.415034000	4.170536000	−2.089510000
C	3.270022000	3.553554000	−1.165838000
C	4.085047000	3.618331000	0.010864000
C	3.554518000	4.274025000	1.157729000
H	4.166044000	4.321493000	2.071524000

C	5.481920000	2.987984000	0.074376000
H	6.080702000	3.304281000	−0.801965000
H	5.997469000	3.387990000	0.967299000
C	5.490142000	1.458451000	0.146932000
C	5.697633000	0.672928000	−1.020875000
C	5.726413000	−0.741588000	−0.938261000
H	5.915586000	−1.332262000	−1.846824000
C	5.473259000	−1.428698000	0.274113000
C	5.221936000	−0.641633000	1.443512000
C	5.271370000	0.772655000	1.373239000
H	5.111987000	1.361840000	2.288719000
C	5.421630000	−2.945236000	0.321621000
H	5.959801000	−3.343410000	−0.559709000
H	5.960346000	−3.312271000	1.216760000
C	3.999419000	−3.515003000	0.325714000
C	3.261016000	−3.576071000	−0.873254000
H	3.650952000	−3.127400000	−1.797688000
C	2.046922000	−4.286129000	−0.967576000
C	1.569328000	−4.994641000	0.171852000
C	2.270698000	−4.870466000	1.384312000
H	1.925602000	−5.397176000	2.284609000
C	3.459058000	−4.118079000	1.493537000
C	0.371324000	−5.943146000	0.095277000
H	0.470861000	−6.596794000	−0.793114000
H	0.423217000	−6.606743000	0.979920000
H	6.028545000	0.752367000	−2.961630000
H	4.609256000	−3.275443000	2.793361000
H	4.503457000	2.505016000	−2.297343000
H	2.294478000	5.404916000	3.051680000
H	4.770939000	−0.729997000	3.348576000
H	0.670832000	−4.901086000	−2.235234000
O	−1.123088000	−5.559853000	−2.291139000
O	−3.252916000	−3.625091000	2.473613000
O	−4.806293000	1.085516000	−2.292219000
O	−5.341496000	−1.871024000	2.398231000
O	−3.864205000	3.781911000	−2.340330000
O	−0.673036000	3.797083000	2.232192000
C	−1.727421000	−5.108034000	−1.145240000
C	−1.002283000	−5.272430000	0.072381000
C	−1.587841000	−4.769228000	1.260963000
H	−1.037669000	−4.840749000	2.210264000
C	−2.841596000	−4.092649000	1.277932000
C	−3.592226000	−3.993485000	0.058639000
C	−3.010398000	−4.507161000	−1.128019000
H	−3.573510000	−4.429401000	−2.070818000
C	−4.985210000	−3.357449000	0.012560000
H	−5.592368000	−3.735821000	0.858373000
H	−5.487539000	−3.702805000	−0.910185000
C	−5.009415000	−1.827823000	0.035023000

C	−5.197192000	−1.118577000	1.245883000
C	−5.233379000	0.291740000	1.261139000
H	−5.327530000	0.819985000	2.223030000
C	−5.066283000	1.052353000	0.077443000
C	−4.906016000	0.334795000	−1.140744000
C	−4.874672000	−1.073785000	−1.150903000
H	−4.748389000	−1.600633000	−2.109075000
C	−4.950550000	2.571950000	0.128848000
H	−5.359916000	2.921656000	1.097007000
H	−5.567145000	3.033406000	−0.667596000
C	−3.530378000	3.132865000	0.000890000
C	−2.667256000	3.154201000	1.115081000
H	−2.953633000	2.662904000	2.055453000
C	−1.450413000	3.863675000	1.098062000
C	−1.094357000	4.609592000	−0.059105000
C	−1.923955000	4.531957000	−1.192709000
H	−1.675409000	5.088932000	−2.106590000
C	−3.118323000	3.781173000	−1.195037000
C	0.115836000	5.544441000	−0.078369000
H	0.046783000	6.268311000	0.757355000
H	0.063733000	6.138089000	−1.010495000
H	−5.402416000	−1.304974000	3.195351000
H	−4.346846000	2.930726000	−2.408530000
H	−4.094874000	−3.101600000	2.434817000
H	−1.673658000	−5.403900000	−3.085497000
H	−4.612991000	0.528910000	−3.073611000
H	−0.065036000	4.563348000	2.306365000
Li	−7.894206000	0.865505000	0.028323000
Li	3.505318000	5.874716000	−0.566684000
Li	7.364483000	−0.008044000	0.559053000
Li	−2.790553000	−6.650285000	0.634456000
H	1.648734000	1.690884000	1.604669000
H	1.065871000	2.124843000	1.857757000
H	−2.185499000	−0.521777000	2.029231000
H	−2.284173000	−1.188879000	2.392991000
H	−0.092116000	2.202752000	−3.314745000
H	−0.595428000	2.557741000	−2.857337000
H	−0.292404000	−2.536241000	−2.122115000
H	−0.882215000	−2.074911000	−1.948387000
H	−1.631812000	0.175168000	−0.822206000
H	−1.885512000	0.864121000	−0.599070000
H	1.414084000	−1.797562000	0.542270000
H	1.157998000	−1.356895000	1.116388000
H	2.726388000	0.495635000	−1.769552000
H	2.618835000	−0.130158000	−1.339931000
H	−8.008481000	−0.204629000	1.862862000
H	−8.730382000	0.045812000	1.935620000
H	−7.737676000	2.612090000	1.131651000
H	−8.414399000	2.777197000	0.810520000

H	−7.322629000	1.601669000	−1.873083000
H	−8.005728000	1.946572000	−1.814983000
H	−8.342711000	−0.995194000	−0.851333000
H	−7.576239000	−1.011782000	−0.803589000
H	−9.976688000	0.955954000	−0.677921000
H	−10.099476000	0.964818000	0.080424000
**Li^+@PPA. Seven H_2 molecules inside the hollow and ten H_2 molecules are on Li^+**			
O	1.684963000	5.517186000	2.263939000
O	3.577699000	3.054766000	−2.350870000
O	4.954001000	−1.280380000	2.491061000
O	5.830783000	1.474294000	−2.266185000
O	3.968750000	−4.030990000	2.520553000
O	1.505545000	−4.150325000	−2.477766000
C	2.233012000	4.932352000	1.155679000
C	1.438730000	4.937088000	−0.033132000
C	1.964326000	4.301240000	−1.177179000
H	1.372740000	4.273223000	−2.102621000
C	3.231218000	3.650118000	−1.191162000
C	4.049726000	3.704524000	−0.016226000
C	3.524153000	4.352455000	1.137043000
H	4.139622000	4.393740000	2.048460000
C	5.445586000	3.071688000	0.037690000
H	6.045105000	3.401534000	−0.833200000
H	5.961122000	3.457121000	0.937059000
C	5.453186000	1.541101000	0.085282000
C	5.664135000	0.775313000	−1.094728000
C	5.702237000	−0.639899000	−1.034392000
H	5.896112000	−1.215498000	−1.951421000
C	5.453320000	−1.347212000	0.167397000
C	5.190971000	−0.580405000	1.347183000
C	5.233867000	0.834751000	1.299951000
H	5.069829000	1.408585000	2.224278000
C	5.407669000	−2.864386000	0.189347000
H	5.975066000	−3.246830000	−0.680633000
H	5.917218000	−3.243831000	1.095895000
C	3.986324000	−3.433303000	0.134908000
C	3.290918000	−3.477378000	−1.090208000
H	3.712895000	−3.016477000	−1.994594000
C	2.077767000	−4.180663000	−1.238706000
C	1.560073000	−4.907068000	−0.126776000
C	2.216078000	−4.794271000	1.112356000
H	1.839316000	−5.331457000	1.993772000
C	3.400761000	−4.046106000	1.275997000
C	0.378958000	−5.872318000	−0.262420000
H	0.490422000	−6.454233000	−1.198134000
H	0.459928000	−6.600802000	0.566306000
H	5.985833000	0.884855000	−3.034811000
H	4.509654000	−3.222919000	2.624881000
H	4.466184000	2.620566000	−2.339242000

H	2.276730000	5.472825000	3.043041000
H	4.712226000	−0.700917000	3.242283000
H	0.680268000	−4.684522000	−2.531211000
O	−1.074955000	−5.233443000	−2.606091000
O	−3.425844000	−4.084648000	2.305491000
O	−5.028416000	1.159869000	−2.294358000
O	−5.140979000	−1.972460000	2.315074000
O	−3.914957000	3.778733000	−2.275798000
O	−0.684111000	3.880519000	2.268102000
C	−1.703061000	−4.916626000	−1.420817000
C	−1.012180000	−5.244570000	−0.226637000
C	−1.649968000	−4.938789000	0.998921000
H	−1.110974000	−5.088824000	1.947427000
C	−2.916885000	−4.299217000	1.058298000
C	−3.605221000	−3.987014000	−0.149797000
C	−2.969180000	−4.301892000	−1.370643000
H	−3.488534000	−4.077430000	−2.314660000
C	−5.004389000	−3.364665000	−0.154256000
H	−5.599560000	−3.787923000	0.678599000
H	−5.512734000	−3.670167000	−1.087733000
C	−5.035206000	−1.839747000	−0.065848000
C	−5.089338000	−1.177632000	1.185809000
C	−5.101957000	0.227751000	1.259537000
H	−5.111496000	0.718531000	2.244996000
C	−5.057530000	1.032520000	0.096480000
C	−5.025318000	0.365552000	−1.155467000
C	−5.015722000	−1.040947000	−1.227437000
H	−4.996268000	−1.530750000	−2.212971000
C	−4.945988000	2.551625000	0.199721000
H	−5.344233000	2.865780000	1.184551000
H	−5.568632000	3.036242000	−0.581304000
C	−3.537606000	3.140120000	0.060238000
C	−2.668520000	3.187080000	1.167765000
H	−2.938869000	2.700293000	2.114861000
C	−1.465430000	3.919584000	1.136044000
C	−1.127731000	4.658333000	−0.030990000
C	−1.963540000	4.554234000	−1.158804000
H	−1.728608000	5.103275000	−2.081123000
C	−3.146476000	3.786227000	−1.143575000
C	0.069502000	5.609791000	−0.069146000
H	0.002324000	6.335650000	0.765016000
H	−0.001883000	6.198817000	−1.002756000
H	−5.138870000	−1.435110000	3.133138000
H	−4.377896000	2.915574000	−2.347679000
H	−4.154728000	−3.411088000	2.307118000
H	−1.620469000	−4.991563000	−3.381283000
H	−4.885244000	0.623865000	−3.101012000
H	−0.081596000	4.652492000	2.326652000
Li	−7.920098000	1.536536000	−0.268981000

Li	3.463422000	5.961908000	−0.572071000
Li	7.326477000	0.075448000	0.474712000
Li	−2.641226000	−7.218112000	1.229664000
H	1.679729000	1.817301000	1.806084000
H	1.082743000	2.251695000	2.024078000
H	−2.095609000	−1.066239000	3.079709000
H	−2.374615000	−1.778557000	3.129734000
H	0.149279000	1.526935000	−1.381456000
H	−0.349528000	2.096189000	−1.259616000
H	−0.160671000	−2.313908000	−2.287816000
H	−0.697995000	−1.843262000	−2.005296000
H	−2.161015000	−0.533430000	0.024760000
H	−2.082091000	0.229522000	0.032383000
H	2.224658000	−1.339357000	0.900728000
H	1.973841000	−0.642810000	1.104512000
H	2.761591000	0.521576000	−2.134575000
H	2.686221000	−0.177763000	−1.829278000
H	−7.792161000	1.128291000	1.765562000
H	−8.396706000	1.599682000	1.812162000
H	−7.721736000	3.634546000	−0.010807000
H	−8.477708000	3.621244000	−0.143783000
H	−7.323358000	1.702331000	−2.287311000
H	−8.069602000	1.885775000	−2.312007000
H	−8.371978000	−0.530010000	−0.290708000
H	−7.643056000	−0.517166000	−0.532543000
H	−10.019945000	1.381021000	−0.841014000
H	−10.103071000	1.662622000	−0.131348000
H	−4.204002000	−6.608015000	−0.009660000
H	−4.482913000	−7.295184000	0.189335000
H	−1.791771000	−8.566264000	−0.292524000
H	−1.778989000	−7.871051000	−0.615922000
H	−1.008490000	−7.634643000	2.540674000
H	−1.110980000	−8.354405000	2.296512000
H	−3.606185000	−6.313586000	2.916770000
H	−3.627737000	−7.066757000	3.070649000
H	−3.480707000	−9.419983000	1.256534000
H	−3.633929000	−9.216342000	1.981764000
**Li^+@PPA. Seven H_2 molecules inside the hollow and fifteen H_2 molecules are on Li^+**			
O	1.431972000	5.224922000	2.583553000
O	3.704683000	3.564082000	−2.216910000
O	5.030564000	−1.329321000	2.585125000
O	5.621299000	1.610335000	−2.115047000
O	3.847733000	−3.947589000	2.527165000
O	1.451761000	−4.259444000	−2.497712000
C	2.042159000	4.786487000	1.429021000
C	1.335020000	4.992522000	0.216388000
C	1.956675000	4.562192000	−0.979538000
H	1.403179000	4.610158000	−1.930279000
C	3.220859000	3.913929000	−0.990731000

C	3.924953000	3.725867000	0.233068000
C	3.306161000	4.165642000	1.423969000
H	3.838280000	4.039209000	2.379184000
C	5.325288000	3.107000000	0.283244000
H	5.930430000	3.496635000	−0.558950000
H	5.817670000	3.455715000	1.210212000
C	5.361339000	1.579219000	0.260663000
C	5.500889000	0.865109000	−0.956934000
C	5.525272000	−0.548791000	−0.966902000
H	5.626628000	−1.083829000	−1.923489000
C	5.360721000	−1.306232000	0.221705000
C	5.200436000	−0.586413000	1.442801000
C	5.229568000	0.826687000	1.451706000
H	5.113263000	1.360312000	2.407562000
C	5.294139000	−2.825683000	0.181944000
H	5.842602000	−3.175393000	−0.714331000
H	5.816270000	−3.245566000	1.063838000
C	3.881114000	−3.414180000	0.128261000
C	3.203797000	−3.508473000	−1.103066000
H	3.631304000	−3.066110000	−2.013986000
C	2.004538000	−4.236670000	−1.246732000
C	1.485019000	−4.938594000	−0.122031000
C	2.121557000	−4.777660000	1.122404000
H	1.741396000	−5.295871000	2.013698000
C	3.290819000	−4.005096000	1.278510000
C	0.313921000	−5.917190000	−0.244239000
H	0.420557000	−6.502803000	−1.178003000
H	0.404708000	−6.640370000	0.588199000
H	5.612209000	1.036698000	−2.909162000
H	4.378755000	−3.128286000	2.613813000
H	4.484400000	2.953872000	−2.167097000
H	1.979360000	5.043140000	3.373752000
H	4.837465000	−0.764449000	3.360268000
H	0.621311000	−4.785802000	−2.533502000
O	−1.156721000	−5.273307000	−2.579408000
O	−3.465347000	−4.110027000	2.348511000
O	−5.145454000	1.100650000	−2.338417000
O	−5.120232000	−1.921782000	2.345459000
O	−3.727061000	3.579101000	−2.324004000
O	−1.130048000	4.088994000	2.584612000
C	−1.767868000	−4.944443000	−1.388439000
C	−1.076135000	−5.289099000	−0.199179000
C	−1.704553000	−4.982411000	1.030636000
H	−1.159705000	−5.137578000	1.974902000
C	−2.960150000	−4.321814000	1.100495000
C	−3.649468000	−3.994531000	−0.102929000
C	−3.023378000	−4.309378000	−1.328775000
H	−3.545163000	−4.075374000	−2.269142000
C	−5.047204000	−3.370803000	−0.092961000

H	−5.620278000	−3.774733000	0.764407000
H	−5.575640000	−3.698831000	−1.007545000
C	−5.084588000	−1.844806000	−0.040071000
C	−5.121470000	−1.152937000	1.196849000
C	−5.172297000	0.253032000	1.236522000
H	−5.172364000	0.768117000	2.209647000
C	−5.166343000	1.030955000	0.053077000
C	−5.128611000	0.333566000	−1.182383000
C	−5.097777000	−1.074369000	−1.220243000
H	−5.086750000	−1.587269000	−2.194166000
C	−5.075326000	2.554647000	0.116686000
H	−5.558974000	2.898337000	1.053452000
H	−5.628283000	3.005678000	−0.732894000
C	−3.659879000	3.142097000	0.094171000
C	−2.936753000	3.288695000	1.293035000
H	−3.333186000	2.892080000	2.237928000
C	−1.726218000	4.010580000	1.356796000
C	−1.237761000	4.644528000	0.179378000
C	−1.926127000	4.433058000	−1.031080000
H	−1.574574000	4.903584000	−1.959729000
C	−3.112117000	3.673807000	−1.104505000
C	−0.057140000	5.619514000	0.206811000
H	−0.156664000	6.291945000	1.081348000
H	−0.149244000	6.260957000	−0.690222000
H	−5.145615000	−1.366325000	3.150778000
H	−4.269642000	2.762365000	−2.368588000
H	−4.145550000	−3.389194000	2.360555000
H	−1.690513000	−4.988285000	−3.347827000
H	−5.016787000	0.545021000	−3.134209000
H	−0.315666000	4.641085000	2.577467000
Li	−7.983531000	1.386822000	−0.198185000
Li	2.877265000	6.792774000	−1.432414000
Li	8.190836000	−0.376345000	0.482185000
Li	−2.695575000	−7.229685000	1.237776000
H	1.167930000	1.831247000	2.532293000
H	0.580941000	2.296799000	2.703075000
H	−0.086088000	−2.325391000	0.164556000
H	−0.355541000	−2.074710000	0.836248000
H	0.478382000	1.584519000	−0.938965000
H	−0.038176000	2.133288000	−0.797393000
H	−0.260753000	−2.473188000	−2.718829000
H	−0.840170000	−1.973910000	−2.650941000
H	−2.193181000	−0.344204000	−0.062513000
H	−2.244013000	0.416413000	0.025179000
H	2.338813000	−0.763717000	0.317627000
H	2.308683000	0.000048000	0.386229000
H	2.839918000	1.039445000	−2.700807000
H	2.744121000	0.298221000	−2.529638000
H	−7.870576000	0.985728000	1.842389000

H	−8.488542000	1.439566000	1.886649000
H	−7.835060000	3.492164000	0.067951000
H	−8.591060000	3.452615000	−0.058162000
H	−7.443920000	1.588259000	−2.243803000
H	−8.202078000	1.716289000	−2.247541000
H	−8.453500000	−0.676807000	−0.251020000
H	−7.711155000	−0.671658000	−0.448149000
H	−10.103413000	1.289006000	−0.777140000
H	−10.181924000	1.425754000	−0.025620000
H	−4.252197000	−6.616085000	−0.001337000
H	−4.529682000	−7.309592000	0.176139000
H	−1.847368000	−8.601019000	−0.278719000
H	−1.831043000	−7.908284000	−0.607015000
H	−1.072541000	−7.666572000	2.551689000
H	−1.175921000	−8.384713000	2.303252000
H	−3.680105000	−6.334098000	2.925477000
H	−3.693369000	−7.088136000	3.076181000
H	−3.547800000	−9.442382000	1.257614000
H	−3.709863000	−9.234693000	1.979673000
H	1.485214000	7.794210000	−2.773061000
H	1.201553000	7.085686000	−2.697230000
H	1.967071000	8.375644000	−0.185989000
H	2.042639000	7.772267000	0.280737000
H	4.013428000	8.679222000	−2.409044000
H	3.763247000	9.012468000	−1.762973000
H	3.935538000	6.461976000	−3.221089000
H	3.893965000	5.723001000	−3.011697000
H	4.426759000	6.337580000	−0.104638000
H	4.644312000	7.046966000	−0.301818000
**Li^+@PPA. Seven H_2 molecules inside the hollow and twenty H_2 molecules are on Li^+**			
O	1.381978000	5.241987000	2.575198000
O	3.692431000	3.541637000	−2.193840000
O	5.148345000	−1.364955000	2.580617000
O	5.659213000	1.649489000	−2.078515000
O	3.927712000	−3.953056000	2.484602000
O	1.455856000	−4.215362000	−2.506819000
C	1.999211000	4.791575000	1.428043000
C	1.301854000	4.987824000	0.208745000
C	1.933192000	4.547424000	−0.978828000
H	1.386886000	4.588879000	−1.934130000
C	3.197711000	3.899014000	−0.974689000
C	3.890710000	3.718914000	0.257189000
C	3.262102000	4.168846000	1.438618000
H	3.786698000	4.049364000	2.398852000
C	5.290139000	3.102265000	0.325579000
H	5.910644000	3.502763000	−0.500154000
H	5.765430000	3.441413000	1.264682000
C	5.338432000	1.575585000	0.285255000
C	5.526509000	0.881867000	−0.935579000

C	5.583082000	−0.525118000	−0.966819000
H	5.697470000	−1.041949000	−1.931997000
C	5.434712000	−1.301338000	0.208957000
C	5.257317000	−0.600240000	1.432196000
C	5.215245000	0.807648000	1.461986000
H	5.081156000	1.322622000	2.425456000
C	5.340256000	−2.823349000	0.135771000
H	5.862839000	−3.161153000	−0.781496000
H	5.856802000	−3.283475000	1.002918000
C	3.921195000	−3.398915000	0.092032000
C	3.228368000	−3.480780000	−1.131030000
H	3.646331000	−3.033661000	−2.043936000
C	2.023023000	−4.202257000	−1.263058000
C	1.512726000	−4.907151000	−0.135724000
C	2.166664000	−4.756904000	1.101560000
H	1.794361000	−5.277680000	1.994673000
C	3.343297000	−3.994337000	1.245563000
C	0.337436000	−5.882478000	−0.248938000
H	0.439808000	−6.470695000	−1.181700000
H	0.431418000	−6.603859000	0.584747000
H	5.724976000	1.089794000	−2.879195000
H	4.422902000	−3.112208000	2.585388000
H	4.484399000	2.946217000	−2.136074000
H	1.925736000	5.066663000	3.369355000
H	4.938121000	−0.810150000	3.359232000
H	0.624145000	−4.740421000	−2.538036000
O	−1.141998000	−5.242604000	−2.579630000
O	−3.441768000	−4.086932000	2.354286000
O	−5.188030000	1.097597000	−2.340934000
O	−5.111515000	−1.911068000	2.351342000
O	−3.763284000	3.573795000	−2.336916000
O	−1.171167000	4.105306000	2.572156000
C	−1.750920000	−4.916085000	−1.386491000
C	−1.053413000	−5.256213000	−0.199545000
C	−1.679561000	−4.950624000	1.031949000
H	−1.132218000	−5.105215000	1.974877000
C	−2.938715000	−4.297356000	1.105236000
C	−3.634588000	−3.976937000	−0.096590000
C	−3.010211000	−4.288892000	−1.323837000
H	−3.536586000	−4.059431000	−2.262744000
C	−5.037893000	−3.365883000	−0.083045000
H	−5.603891000	−3.772746000	0.777618000
H	−5.566367000	−3.701708000	−0.994744000
C	−5.089662000	−1.840373000	−0.034495000
C	−5.126346000	−1.145198000	1.200428000
C	−5.188853000	0.260282000	1.236617000
H	−5.186402000	0.778211000	2.208218000
C	−5.195660000	1.034405000	0.050738000
C	−5.158946000	0.333783000	−1.182943000

C	−5.115751000	−1.073749000	−1.216948000
H	−5.104715000	−1.589490000	−2.189361000
C	−5.112368000	2.558650000	0.108910000
H	−5.596458000	2.904269000	1.044819000
H	−5.667029000	3.004249000	−0.742405000
C	−3.698291000	3.148814000	0.083857000
C	−2.976139000	3.301047000	1.282431000
H	−3.373614000	2.910529000	2.229425000
C	−1.764330000	4.021027000	1.343728000
C	−1.272385000	4.645746000	0.162632000
C	−1.960839000	4.429260000	−1.046822000
H	−1.606751000	4.893308000	−1.977763000
C	−3.149059000	3.673275000	−1.117183000
C	−0.088783000	5.617416000	0.184090000
H	−0.191093000	6.300863000	1.049837000
H	−0.175506000	6.247364000	−0.721589000
H	−5.138917000	−1.353342000	3.155041000
H	−4.309158000	2.759282000	−2.377490000
H	−4.125345000	−3.368901000	2.367315000
H	−1.680256000	−4.960958000	−3.346233000
H	−5.057949000	0.540861000	−3.135727000
H	−0.355575000	4.656268000	2.564861000
Li	−8.012538000	1.394871000	−0.193737000
Li	2.880243000	6.756313000	−1.425947000
Li	8.216438000	−1.374458000	0.610582000
Li	−2.672801000	−7.199166000	1.246038000
H	1.117352000	1.838061000	2.535052000
H	0.531316000	2.304119000	2.707672000
H	−0.076254000	−2.302571000	0.164682000
H	−0.342736000	−2.056933000	0.839286000
H	0.470600000	1.572762000	−0.947982000
H	−0.051739000	2.118660000	−0.817162000
H	−0.279512000	−2.425536000	−2.711792000
H	−0.860526000	−1.928786000	−2.640667000
H	−2.214951000	−0.329363000	−0.069336000
H	−2.267681000	0.432084000	0.010077000
H	2.361471000	−0.751780000	0.322023000
H	2.325128000	0.010312000	0.402454000
H	2.863519000	1.028852000	−2.699158000
H	2.775326000	0.285282000	−2.534424000
H	−7.888368000	0.997379000	1.845162000
H	−8.509865000	1.446190000	1.891047000
H	−7.867496000	3.497978000	0.068193000
H	−8.623965000	3.459211000	−0.055266000
H	−7.481804000	1.588209000	−2.239900000
H	−8.239929000	1.716690000	−2.241102000
H	−8.473632000	−0.675587000	−0.239800000
H	−7.732385000	−0.666677000	−0.440990000
H	−10.136793000	1.292644000	−0.763464000

H	−10.212168000	1.432937000	−0.012308000
H	−4.230962000	−6.590716000	0.008754000
H	−4.507827000	−7.284150000	0.187526000
H	−1.824480000	−8.569120000	−0.270533000
H	−1.808401000	−7.877478000	−0.601145000
H	−1.046731000	−7.630358000	2.557399000
H	−1.151198000	−8.348698000	2.309994000
H	−3.656163000	−6.306463000	2.934988000
H	−3.667867000	−7.060502000	3.086078000
H	−3.518340000	−9.413026000	1.272797000
H	−3.679302000	−9.204219000	1.994771000
H	1.495445000	7.766195000	−2.786059000
H	1.209676000	7.058681000	−2.708865000
H	1.957337000	8.348810000	−0.210225000
H	2.038743000	7.754411000	0.266900000
H	4.030749000	8.650961000	−2.411233000
H	3.774485000	8.988337000	−1.769645000
H	3.942447000	6.430296000	−3.207359000
H	3.898777000	5.690033000	−3.002661000
H	4.420530000	6.310423000	−0.098243000
H	4.640193000	7.020480000	−0.290696000
H	9.085524000	−0.997338000	−1.325523000
H	8.414416000	−0.625369000	−1.333472000
H	10.447211000	−1.143213000	0.822706000
H	10.325162000	−1.680009000	1.358923000
H	8.119509000	−3.407679000	0.082429000
H	8.875064000	−3.315785000	−0.009645000
H	8.664761000	0.582524000	1.188451000
H	7.901097000	0.640149000	1.130381000
H	7.493795000	−1.894617000	2.538007000
H	8.237566000	−2.078929000	2.588557000
